# Supplementary material for: Genotype by environment interaction and yield stability analysis of durum wheat (Triticum turgidum var. durum) varieties using AMMI and GGE biplot analysis in Amahara Region, Northwest Ethiopia
Source: PLoS One. 2026 Mar 13;21(3):e0342401. doi: 10.1371/journal.pone.0342401 (PMC12987450; doi:10.1371/journal.pone.0342401)
Supplement: Attachment — Submitted filename: PONE-D-25-30070_R1_reviewer.pdf [file pone.0342401.s007.pdf]

PLOS ONE

Genotype by Environment Interaction and Yield Stability Analysis of Durum Wheat (Triticum turgidum var. durum) Varieties Using AMMI and GGE biplot Analysis in Amahara Region, Northwest Ethiopia

--Manuscript Draft--

**Manuscript Number:**	PONE-D-25-30070R1
**Article Type:**	Research Article
**Full Title:**	Genotype by Environment Interaction and Yield Stability Analysis of Durum Wheat (Triticum turgidum var. durum) Varieties Using AMMI and GGE biplot Analysis in Amahara Region, Northwest Ethiopia
**Short Title:**	Genotype by Environment Interaction and Yield Stability Analysis of Durum Wheat
**Corresponding Author:**	Alemnesh Eskezia<br>Debre Markos University College of Agriculture and Natural Resource<br>debre markos, Ethiopia ETHIOPIA
**Keywords:**	AMMI; GGE; GIE; PCA; stability; wheat
**Abstract:**	Durum wheat is a staple crop for over one- third of the global population; however, its production in Ethiopia, particularly in the Amhara Region, faces major challenges due to limited adaptable varieties, poor management practices, and environmental constraints. Newly released wheat varieties often fail in unfamiliar environments, leading to yield losses. To address this issue, a study was conducted to evaluate the adaptability, performance, and yield stability of recently released high-performing durum wheat cultivars in the Amahara region. The field trials were conducted over two consecutive years (2022/2023 and 2023/2024) at three districts ; Simada, Bichena, and Dabat, using a Randomized Complete Block Design with three replications. Data were collected on main agronomic traits, including growth, phenological, and yield traits such as grain yield. SAS 9.4 and R software were used, showing significant differences in crop phenological stages, growth, and yield parameters across years and locations for tested traits. Additive Main Effect and Multiplicative Interaction(AMMI) and Genotype and Genotype × Environment (GGE) biplot analysis were used to study genotype by environmental interaction(GEI). Genotype, location, and year interactions significantly influenced all durum wheat genotypes. The combined analysis of variance for all tested traits including grain yield, showed highly significant effects (p< 0.001). Grain yield varied from 4.5 t ha-1in Dabat to 2.29 t ha-1in Simada. Among the genotypes, Tesfaye had the highest mean yield (3.74 t ha-1), while Wehabit had the lowest (2.75 t ha-1). AMMI analysis of result revealed significant interactions and differences between genotypes and environments, with genotype (16.6%), environment (29.78%), and genotype x environment interaction (16.84%).The GGE biplot and AMMI analysis identified Tesfaye and Don metteo as stable genotypes, while Simada and Bichena as representative environments. The GGE biplot analysis grouped the six test environments and nine genotypes into two mega-environments and six genotype groups. This study identified that Tesfaye most productive and stable wheat variety in the study area and suggested farmers should adopt it to increase wheat yield per unit area of land.
**Order of Authors:**	Alemnesh Eskezia
	Alamir Ayenew
	Yohannes Azene
	Mulugeta Ayitenew
	Kelemu Nakachew
**Response to Reviewers:**	Manuscript. Number: PONE-D-27-30070<br>Journal: Plos one<br>Title: Genotype by Environment Interaction and Yield Stability Analysis of Durum Wheat (Triticum turgidum var. durum) Varieties Using AMMI and GGE biplot Analysis

Powered by Editorial Manager® and ProduXion Manager® from Aries Systems Corporation

in Amahara Region, Northwest Ethiopia
Dear Editor and Reviewers
We would like to thank both the editor and the reviewers for your contribution to this manuscript by filtering out the technical and editorial errors in the scientific research writing that could increase the reliability and scientific exchange of the research output. We also admire your effort to see the details of our manuscript taking your golden time and we value all the comments. Further, we would be very glad to publish our manuscript in the Plos one journal if accepted. We believe that we have addressed all comments as suggested in the text and point-by-point response. Hence, kindly consider our revisions accordingly.
Response to the comments
Title section reviewer comment.
Title: needs minor modification and I suggest it to be;
"Genotype by Environment Interaction and Yield Stability Analysis of Durum Wheat (Triticum turgidum var. durum) Varieties Using AMMI and GGE biplot Analysis in Amahara state or Region, Northwest Ethiopia".
Response_ Thank you for your valuable comments. We have modified our manuscript title based on the reviewer's feedback and suggestions.
Genotype by Environment Interaction and Yield Stability Analysis of Durum Wheat (Triticum turgidum var. durum) Varieties Using AMMI and GGE biplot Analysis in Amahara Region, Northwest Ethiopia
Abstract section reviewer comment.
Abstract: this section needs major modification;
1. Abstract poorly composed though the study has good results.
Response_ We have updated the section by incorporating the main results into the abstract.
2.It should be written again because some data presented there has mistakes
Response: We have rewritten and updated the entire abstract based on the study results.
3. Some important result information not presented
Response_ We have updated it
4.There are lots of grammatical & typographical mistakes
Response_ All grammatical and typographical errors have been corrected as per the reviewer's comment.
5.I tried to correct some of them using the MSWord track review method in the attached file
Response_Thank you for your valuable comments. We have reviewed them and made the necessary corrections.
6.The AMMI analysis Anova output was reported as the result of combined anova result
Response_ We have corrected the AMMI analysis ANOVA output to accurately present it separately from the combined ANOVA results.
Introduction section reviewer comment.
1.It has good information but presented poorly, it is highly disorganized, paragraphs should be reshuffled.
Response_ the necessary corrections have been made, and the paragraphs have been reorganized to improve clarity and structure.
2.In text citations didn't follow the journal's guideline
Response_ In-text citations have been revised in accordance with the journal's guidelines.
3.Lots of sentences not clearly written. Several of them need to be rewritten
Response_ We have rewritten unclear sentences to improve clarity and readability
4.Some topics like AMMI & GGE described repeatedly about their use and assumptions in detail, but no previous literatures on durum wheat presented that show their practical applications
Response_ We have revised the sections on AMMI and GGE to reduce repetition and included relevant previous literature on their applications in related crops.
5.There is no statement or justifications why the authors selected the AMMI & GGE biplot models
Response_ We have added a clear justification for selecting the AMMI and GGE biplot models, highlighting their suitability for analyzing genotype by environment interactions in our study
6.Problem statement or the need to do this study not described sufficiently
Response_ The problem statement has been strengthened to provide a more

comprehensive justification for conducting this study.
7.Objectives of the research not stated clearly
Response _We have revised the objectives section to clearly and explicitly state the research goals
Materials & methods section reviewer comment.
1.the sub-title 2.1 should be modified; here you are giving the detail account of the study (experimental) area
Response _ we update it
2.Table 1.Geographical description of the study area; It would be more informative if you can incorporate air temperature, and rainfall data of each year for the specific locations for the specific years. Specific geographic coordinates (including altitude) of the trial sites & if possible soil physic-chemical properties.
Response _ We have included air temperature and rainfall data for each year and the specific geographic coordinates, including altitude, of the trial sites. However, soil physicochemical properties were not available and thus could not be included
3.2.2. Experimental material and design; first 4-5 line needs to be rewritten
Response-We have rewritten the first 4–5 lines of section 2.2 to improve clarity and coherence
4.Table 3; Can you include their pedigree for each variety? You better include their maturity period, yielding ability, areas of adaptation (recommendation), level of disease resistance, etc.
Response- We have included the pedigree, maturity period, yielding ability, recommended areas of adaptation, and disease resistance levels for each variety in Table 3 as suggested.
5.2.3. Data Collected; you have not described how you collected the data. At least you can insert citations of a published material that explains how you collected the data. So that readers get knowledge of how to do similar experiments using same or different materials.
Response- We have included a detailed description of the data collection methods along with relevant citations to guide readers on how to conduct similar experiments.
6.GGE biplot analysis- Which method or formula did you use? You need to show the formula you have used to compute the analysis
Response- The manuscript now includes a detailed description of the GGE biplot analysis method along with the corresponding formula, as requested
Results and discussions section reviewer comment
•Same problem was observed in the in text citation as above
1.3.2. Performance of genotypes - What about the performance of these durum wheat genotypes in previous studies by other researchers? Please cite previous works that either agree or contradict with your findings to make your discussion complete
Response- Relevant literature discussing the performance of durum wheat genotypes in previous studies has been cited to contextualize and strengthen the discussion of our results.
2.What is this MSE? Is it the std of the mean values?
Response-Its MSE (Mean Square Error) is not the standard deviation.
3.3.3. Genotype performance at each tested location- this sub-topic also lacks supporting literatures for the findings presented similar to sub-topic 3.2
Response- We have added relevant supporting literature to the sub-topic on genotype performance at each tested location.
4.3.4. Additive Main Effects and Multiplicative Interaction (AMMI) Analysis- One table is missing from AMMI analysis; the significant IPCAs (usually 1&2) give vector values for each genotype that indicates how much each genotype contributes to the GEI. Absolute value of the IPCA score determines its stability. Low value close to zero indicates the genotype being stable. You didn’t indicate which genotypes are stable using the AMMI analysis either using the table or the AMMI biplot 1 graph.
Response - We have included the AMMI analysis results by adding Table 7, which presents YSI, ASV, IPCA1, and IPCA2 values. Based on these, we have identified and indicated the stable genotypes as per the reviewer’s suggestion.
5.Please refer the following publication for better understanding of the results and interpretation of AMMI analysis; Kassie MM, Abebe TD, Desta EA, Tadesse W. Multi-Environment Evaluation of Bread Wheat Genotypes for Yield Stability in Ethiopia Using AMMI and GGE-Biplot Analyses. Crop Breed Genet Genom. 2025;7(2):e250004.
Response - Thank you for the helpful reference. We have reviewed the suggested publication by Kassie et al. (2025) and incorporated relevant insights to enhance the interpretation and discussion of the AMMI analysis results in our manuscript.

	6.It appears that there is error in the table numbering, check it, see the comment in the manuscript file<br>Response - We have carefully reviewed the manuscript and corrected the table numbering errors as pointed out.<br>7.On page 17, the values you presented from table 7 about the proportions of the sum of squares of the AMMI anova seems to be incorrect. In addition, another table that lists IPCA scores for each genotype & even environments is missing. The IPCA scores help in identifying the relative contribution of genotypes for the GEI determines which genotype is stable/unstable. Please read the paper Kassie, et al (2025) and some recently published papers in durum wheat by Liyew et al. (2025) too.<br>Response -We have corrected the values from Table 7 regarding the sum of squares proportions in the AMMI ANOVA and added the IPCA scores for each genotype and environment as subcomponents within Table 7. This addition facilitates identification of genotype stability and contribution to GEI. We also reviewed and incorporated insights from Kassie et al. (2025) and Liyew et al. (2025) to strengthen our analysis.<br>8.Figure 3, the mega environment grouping not clearly displayed<br>Response – We have updated Figure 3 to clearly display the mega-environment groupings, making them easier to identify<br>9.Figure 4, concentric circles not visible<br>Response – We have enhanced Figure 4 to improve the visibility of the concentric circles as per your comment<br>10.Page 26, there is unfinished sentence; either correct it or delete it.<br>Response – he unfinished sentence on page 26 has been corrected to ensure clarity and completeness<br>11.3.7 stability parameter; here, you have produced a beautiful table (the last table) for the stability analysis, but it was not exploited fully. You explained only one of the stability models that favors high grain yield. Please use the most recent stability models too like ASV, YSI<br>Response –Thank you for the valuable suggestion. We have expanded the stability analysis section to include and discuss additional recent stability models such as ASV and YSI, providing a more comprehensive interpretation of the results<br>12.References; Please use the journal's guideline for in text citations & style of referencing<br>Response – We have carefully revised the in-text citations and reference list to fully comply with the journal's guidelines
**Additional Information:**	
**Question**	**Response**
**Financial Disclosure**<br><br>Enter a financial disclosure statement that describes the sources of funding for the work included in this submission. Review the submission guidelines for detailed requirements. View published research articles from *PLOS ONE* for specific examples.<br><br>This statement is required for submission and **will appear in the published article** if the submission is accepted. Please make sure it is accurate.	The author(s) received no specific funding for this work.

Powered by Editorial Manager® and ProduXion Manager® from Aries Systems Corporation

**Funded studies**<br>Enter a statement with the following details:<br>• Initials of the authors who received each award<br>• Grant numbers awarded to each author<br>• The full name of each funder<br>• URL of each funder website<br>• Did the sponsors or funders play any role in the study design, data collection and analysis, decision to publish, or preparation of the manuscript?<br><br>Did you receive funding for this work?	
**Competing Interests**<br><br>Use the instructions below to enter a competing interest statement for this submission. On behalf of all authors, disclose any competing interests that could be perceived to bias this work—acknowledging all financial support and any other relevant financial or non-financial competing interests.<br><br>This statement is required for submission and **will appear in the published article** if the submission is accepted. Please make sure it is accurate and that any funding sources listed in your Funding Information later in the submission form are also declared in your Financial Disclosure statement.<br><br>View published research articles from *PLOS ONE* for specific examples.	no- The authors have not declared any conflict of interest.

Powered by Editorial Manager® and ProduXion Manager® from Aries Systems Corporation

**NO authors have competing interests**<br><br>Enter: *The authors have declared that no competing interests exist.*<br><br>**Authors with competing interests**<br><br>Enter competing interest details beginning with this statement:<br><br>*I have read the journal's policy and the authors of this manuscript have the following competing interests: [insert competing interests here]*<br><br>* typeset	
**Ethics Statement**<br><br>Enter an ethics statement for this submission. This statement is required if the study involved:<br><br>• Human participants<br>• Human specimens or tissue<br>• Vertebrate animals or cephalopods<br>• Vertebrate embryos or tissues<br>• Field research<br><br>Write "N/A" if the submission does not require an ethics statement.<br><br>General guidance is provided below. Consult the submission guidelines for detailed instructions. **Make sure that all information entered here is included in the Methods section of the manuscript.**	NA

**Format for specific study types**<br><br>**Human Subject Research (involving human participants and/or tissue)**<br>• Give the name of the institutional review board or ethics committee that approved the study<br>• Include the approval number and/or a statement indicating approval of this research<br>• Indicate the form of consent obtained (written/oral) or the reason that consent was not obtained (e.g. the data were analyzed anonymously)<br><br>**Animal Research (involving vertebrate animals, embryos or tissues)**<br>• Provide the name of the Institutional Animal Care and Use Committee (IACUC) or other relevant ethics board that reviewed the study protocol, and indicate whether they approved this research or granted a formal waiver of ethical approval<br>• Include an approval number if one was obtained<br>• If the study involved *non-human primates*, add *additional details* about animal welfare and steps taken to ameliorate suffering<br>• If anesthesia, euthanasia, or any kind of animal sacrifice is part of the study, include briefly which substances and/or methods were applied<br><br>**Field Research**<br><br>Include the following details if this study involves the collection of plant, animal, or other materials from a natural setting:<br>• Field permit number<br>• Name of the institution or relevant body that granted permission	
**Data Availability**<br><br>Authors are required to make all data underlying the findings described fully available, without restriction, and from the time of publication. PLOS allows rare exceptions to address legal and ethical concerns. See the PLOS Data Policy and FAQ for detailed information.	No - some restrictions will apply

A Data Availability Statement describing where the data can be found is required at submission. Your answers to this question constitute the Data Availability Statement and **will be published in the article**, if accepted.<br><br>**Important:** Stating 'data available on request from the author' is not sufficient. If your data are only available upon request, select 'No' for the first question and explain your exceptional situation in the text box.<br><br>Do the authors confirm that all data underlying the findings described in their manuscript are fully available without restriction?	
**Describe where the data may be found in full sentences. If you are copying our sample text, replace any instances of XXX with the appropriate details.**<br><br>• If the data are **held or will be held in a public repository**, include URLs, accession numbers or DOIs. If this information will only be available after acceptance, indicate this by ticking the box below. For example: *All XXX files are available from the XXX database (accession number(s) XXX, XXX.*).<br>• If the data are all contained **within the manuscript and/or Supporting Information files**, enter the following: *All relevant data are within the manuscript and its Supporting Information files.*<br>• If neither of these applies but you are able to provide **details of access elsewhere**, with or without limitations, please do so. For example:<br><br>*Data cannot be shared publicly because of [XXX]. Data are available from the XXX Institutional Data Access / Ethics Committee (contact via XXX) for researchers who meet the criteria for access to confidential data.*<br><br>*The data underlying the results presented in the study are available from (include the name of the third party*	Datasets generated in this study are available from the corresponding author upon reasonable request.

and contact information or URL).<br>• This text is appropriate if the data are owned by a third party and authors do not have permission to share the data.<br><br>* typeset	
Additional data availability information:	

Powered by Editorial Manager® and ProduXion Manager® from Aries Systems Corporation

Manuscript

Click here to access/download;Manuscript;Durum wheat manuscrip Revise main article file 1.docx

Genotype by Environment Interaction and Yield Stability Analysis of Durum Wheat (*Triticum turgidum* var. *durum*) Varieties Using AMMI and GGE biplot Analysis in Amahara Region, Northwest Ethiopia

Alemnesh Eskezia*[1], Alamir Ayenew,[2] Yohannes Azene[3], Mulugeta Aytenew[1], Kelemu Nakachew [1]

[1]Department of Plant Science, College of Agriculture and Natural Resources, Debre Markos University, P.O.Box 269. Ethiopia.

[2]Fogera National Rice Research and Training Center, Ethiopian Institute of Agricultural Research, Ethiopia.

[3]Gonder Agricultural Research Center, P.O.Box 1337, Gondar, Ethiopia

Corresponding author contact: alemnesh_eskezia@dmu.edu.et. Phone number: +251918314065

Abstract

Durum wheat is a staple crop for over one- third of the global population; however, its production in Ethiopia, particularly in the Amhara Region, faces major challenges due to limited adaptable varieties, poor management practices, and environmental constraints. Newly released wheat varieties often fail in unfamiliar environments, leading to yield losses. To address this issue, a study was conducted to evaluate the adaptability, performance, and yield stability of recently released high-performing durum wheat cultivars in the Amahara region. The field trials were conducted over two consecutive years (2022/2023 and 2023/2024) at three districts ; Simada, Bichena, and Dabat, using a Randomized Complete Block Design with three replications. Data were collected on main agronomic traits, including growth, phenological, and yield traits such as grain yield. SAS 9.4 and R software were used, showing significant differences in crop phenological stages, growth, and yield parameters across years and locations for tested traits. Additive Main Effect and Multiplicative Interaction(AMMI) and Genotype and Genotype × Environment (GGE) biplot analysis were used to study genotype by environmental interaction(GEI). Genotype, location, and year interactions significantly influenced all durum wheat genotypes. The combined analysis of variance for all tested traits including grain yield, showed highly significant effects ($p< 0.001$). Grain yield varied from 4.5 t ha^{-1}in Dabat to 2.29 t ha^{-1}in Simada. Among the genotypes, Tesfaye had the highest mean yield (3.74 t ha^{-1}), while Wehabit had the lowest (2.75 t ha^{-1}). AMMI analysis ~~of result~~ revealed significant interactions and differences between genotypes and environments, with genotype (16.6%),

environment (29.78%), and genotype x environment interaction (16.84%).The GGE biplot and AMMI analysis identified Tesfaye and Don metteo as stable genotypes, while Simada and Bichena as representative environments. ~~The GGE biplot analysis grouped the six test environments and nine genotypes into two mega environments and six genotype groups.~~ This study identified that Tesfaye most productive and stable wheat variety in the study area and suggested farmers should adopt it to increase wheat yield per unit area of land.

Keywords; ASV, PCA, Stability, Wheat, YSI

INTRODUCTION

Durum wheat (*Triticum turgidum var. durum*) is an indigenous tetraploid wheat species (2n = 4x = 28) that has been cultivated in Ethiopia since ancient times [1]. The country is considered one of the centers of diversity for tetraploid wheat [2, 3]. Traditionally, this crop grown is in heavy black clay soils (vertisols) under rain-fed conditions at altitudes ranging from 1800 to 2800 meters above sea level [4]. The proportion of durum wheat in the total wheat-growing area has declined significantly due to the introduction of semi-dwarf, fertilizer-responsive bread wheat varieties, resulting in a shift where bread wheat now occupies the largest share of production [5, 6]. However, the area dedicated to durum wheat is expected to increase because of rising urban demand for pasta products and the expansion of irrigated wheat production in Ethiopia [7, 8].

Wheat is essential for Ethiopia's agricultural development and food security, as it is a significant source of protein and energy [9-11]. Although wheat is a staple for one-third of the global population, Ethiopia struggles to achieve self-sufficiency due to variable environments. Studying GEI helps identify stable, high-yielding varieties adapted to these diverse conditions [12, 13]. Wheat is crucial to the country's agricultural plans and sustainable development initiatives because of its nutritional value and versatility[14].

In Ethiopia, wheat covered approximately 1.87 million hectares, yielding approximately 5.8 million tons of grain during the 2021/22 cropping season [15]. In the Amhara region, wheat cultivation spanned 689,614.06 hectares, yielding 1.92 million tons with productivity of 2.8 t ha^{-1} [15]. Overall, wheat is a staple crop in Ethiopia and is vital for food security and economic

growth [16]. However, its productivity is low in many regions, including the Amhara Region. This is because of different yield-limiting factors such as diseases, pests, climate variability, overuse of local varieties, lack of high-yielding and stable varieties, and poor communication between farmers and researchers. Nowadays, Ethiopia's regional and national agricultural research institutes have released several durum wheat varieties [17, 18]. Conversely, many of these varieties have not undergone comprehensive evaluation across the country, particularly in the northwestern regions [19]. To fill this gap, it is essential to generate evidence on genotypic performance and adaptability under diverse environmental conditions. Such information is crucial for devising effective intervention strategies to enhance crop productivity and promote dietary self-sufficiency [20].

Durum wheat breeding programs in Ethiopia rely on elite lines from various sources, but their performance often varies across environments due to genotype-by-environment interaction (GEI)[21, 22]. To ensure stable and high-yielding varieties, multi-environment trials (METs) are essential for assessing performance and stability [23, 24]. GEI complicates selection decisions because genotypes may perform inconsistently across locations, exhibiting either non-crossover (differences in magnitude) or crossover (changes in rank) interactions [25]. Therefore, relying solely on mean yield is therefore inadequate, highlighting the need for robust GEI analysis to guide breeding and variety release [26, 27].

The AMMI model integrates ANOVA for additive effects and PCA for multiplicative interactions, enabling clear stability assessment through metrics such as the AMMI Stability Value. AMMI and GGE biplot were chosen due to strong GEI in the data. AMMI analyzes stability, while GGE helps visualize ideal genotypes and environments. Together, they offer a comprehensive view for genotype evaluation. Although these methods have been extensively applied in crops such as ~~like~~ maize, rice, and bread wheat, their use in durum wheat multi-environment trials remains limited in Ethiopia[28, 29]. Previous studies in Ethiopia and elsewhere have demonstrated the effectiveness of AMMI and GGE in identifying stable and high-yielding durum wheat varieties, such as the works of [18, 29, 30] and [31], which reported the identification of genotypes with broad adaptability and mega-environment delineation. Our study builds on these findings by applying both methods to durum wheat in the Amhara region under rainfed conditions, generating practical insights for stability, adaptability, and variety

recommendation across diverse agro-ecologies. This dual-method approach supports robust decision-making in durum wheat breeding and variety recommendation [32, 33].

Given the significant influence of environmental variability on genotype performance, stability assessment is crucial in plant breeding to identify genotypes with consistent yield across environments [34]. Stability measures, such as Shukla's stability variance, which quantifies each genotype's contribution to GEI with lower values indicating greater stability [35], and the regression-based method developed by [36],which assesses genotype sensitivity and adaptability, have been foundational in this field. Genotypic stability is commonly categorized into two forms: static stability, where genotype performance remains relatively constant across environments, and dynamic stability, where performance changes in a consistent and predictable manner in response to environmental variation [37]. These complementary concepts are critical for identifying genotypes that combine high productivity with reliable performance across diverse environmental conditions. Therefore, the current study was conducted to evaluate the adaptability, performance, and yield stability of recently released high-performing durum wheat cultivars in the Amahara Region, northwestern Ethiopia.

2. MATERIALS AND METHODS

2.1. Descriptions of the Study area

The study was conducted across three wheat-producing districts: Simada, Bichena, and Dabat, situated within the Aamara region, Ethiopia, with permission from the Amhara Regional Agricultural Research Institute (ARARI) and Debre Markos university (DMU) (Table 1). Specifically, it was conducted at the Farmers Training Centers (FTC) in each district during the main crop or rainy seasons between June and December 2022-2024 for two consecutive years. Soils in the tested location are Vertisols, with a pH of 5.5–7.0, moderate organic matter, and clayey texture. They contain moderate organic matter but face challenges such as phosphorus deficiency, erosion, and waterlogging in lowland areas. The locations were chosen purposively based on wheat production potential and soil type.

Table 1.Geographical description of the study area

Location	Code Year 2021/2022	Code Year 2022/2023	Alt. (masl)	Temp. min & max values (°C) Each year		RF. Ave.(mm)		Soil type	Geographic location	
				2022	2023	2022	2023		Lat	Lon
Simada	E1	E4	300-2220	19-27	19-25	1012	1320	Vertisol	10 °22' N	37° 47' E
Bichena	E2	E5	2250	15.19	13-21	882	1256	Vertisol	10 38' N	10° 37' E
Dabat	E3	E6	2446	17-23	12-19	1320	1470	Vertisol	10°21` N	37° 43`E

Where: Alt: Altitude, Temp: temperature, and RF: Rainfall, Lat: latitude, Lon: longitude

Source:Districta dministrative office

2.2. Experimental material and design

Seeds of eight improved, high-yielding durum wheat varieties were obtained from the Kulumsa Agricultural Research Center, and one variety was collected from the Adet Agricultural Research Center in Amhara, Ethiopia (Table 2). All varieties were evaluated under rainfed conditions during the 2022 and 2023 main cropping seasons and were sown on well-prepared experimental plots. These nine varieties were tested across three environments (locations). Each experimental unit (plot) consisted of nine rows with 20 cm spacing between rows, resulting in a plot area of 3.6 m^2 (1.8 m × 2 m). The field was plowed three times before sowing, and planting rows were prepared using a hand-pulled row marker. Seeds were manually planted using the hand drilling technique. A spacing of 0.5 m was maintained between plots, and 1 m was left between blocks. Since the two years (2022 and 2023) were also considered as blocks, the six combinations of the field blocks and years were used as blocks in the multilocation trial model. For each plot, a standard fertilizer application of 100 kg/ha NPS and 150 kg/ha urea was applied, following recommendations for durum wheat. Half of the urea and the full amount of NPS were applied during planting, while the remaining half of the urea was applied at the mid-tillering stage. A seed rate of 150 kg/ha was used. Weeding was carried out manually across all experimental fields at the three locations to manage weeds. No herbicides or fungicides were applied for weed or disease control.

Table 2. Variety used in the study

S. N	Variety code	Variety name	Pedigree	Year of release	Center	Maturity period	Disease resistance levels	Yied on station	Area of daptation
1	G1	Fetan	CDSS02B00643 S-0Y-0M-1Y4M-04-Y-0B-2Y	2018	DZARC	95-110	Moderately resistance	46-50	500-2600
2	G2	Don Matteo	43IDSNMeh 2011-82	2018	CGS Italian	115-130	Moderately resistance	50-55	1500-2750
3	G3	Alemtena	43IDSNMeh 2011-130	2015	DZARC	95-110	Moderately resistance	44-50	500-2600
4	G4	Tesfaye	ARMENT//SRN-NIGRIS-4/3/CANED-9.1/4/TOSKA-26RASCON-37//SNITSN /5/PLAYERO	2017	DZARC	110-130	Moderately resistance	50-55	1600-2800
5	G5	Wehabit	Waha (plc/Ruff//Gta/Rtte)	2017	Mekelle University	NA	Moderately resistance	NA	NA
6	G6	Rigeat	Acc#208304	2017	Mekelle University	NA	Moderately resistance	NA	NA
7	G7	Utuba	(= Icajihan42) Omruf1/Stojocri2/3/1718/BeadWheat 24//Karimï¿½	2015	DZARC	105-118	Moderately resistance	53-57	1880-2700
8	G8	Mangudo	Mangudo/Mekuye/DZ 2013 meh DW F1 P#20/DZ 2014 meh DW F2 P#19-8	2012	SARC/ORARI	110-124	Moderately resistance	45-50	1880-2700
9	G9	Toltu	4/B/R9096#21001(980SN Patho)	2010	SARC/ORARI	NA	Moderately resistance	NA	NA

2.3. Data Collected

The data for the eleven traits were collected on a plant-and-plot basis. Data were obtained on a plot basis based on all plants in the central rows of each plot, leaving two border row from each side of the plots. The ten representative plants per plot were randomly selected from the central rows for recording data on plant basis. Agronomic traits such as crop phenology, growth, yield, and yield components were assessed during the study period. Crop phenology included maturity day and days to heading, whereas growth parameters, such as plant height, number of productive tillers, number of grains per spike, number of spikelets per spike, spike length, and yield-related traits, including biomass yield, thousand seed weight, number of seed per spikelet, grain yield, and harvest index, were recorded.

Days to heading (days): The number of days from sowing to when the tips of the spike first emerged from the main shoot in 50% of the plant population within a plot was recorded.

Days to 90% maturity (days): The number of days from sowing to physiological maturity was recorded. Physiological maturity was determined when 90% of the crop stands in a plot showed a light yellow (straw) color in their stems, leaves, and floral bracts.

Number of productive tillers (no): The number of productive tillers was determined at maturity by counting all spike-producing tillers of 10 plants per plot.

Number of spikelets per spike (no): The number of spikelets per spike was counted from ten representative spikes per plant, and the average was calculated.

Plant height (cm) was measured from the base of the main stem to the tip of the spike from an average of ten randomly selected plants in the central four rows in each plot.

Spike length (cm): This was recorded at the physiological maturity stage by measuring the middle rows of 10 randomly tagged plants from the base of the spike to the tip of the spike (excluding the awns), and the average length of the plant was calculated.

Biomass yield (ton ha^{-1}): Aboveground total biomass was recorded after sun-drying, attained constant weight, and then converted to t ha^{-1}.

Grain yield (ton ha^{-1}): The weight of air-dried seeds harvested from each plot was recorded. Then, the yield from the net plot area was converted into t ha^{-1}

Harvest index (%): The ratio of grain yield per plot to biological yield per plot is expressed as a percentage.

$$HI (\%) = \frac{\text{Grain Yield}}{\text{Biomass Yield}} \times 100$$

2.4. Data Analysis

Data were analyzed using R statistical software version 4.4.1 (R Core Team, 2021). Analysis of variance (ANOVA) was conducted on the multi-environment data using the aov function from the car package [38] to evaluate the significance of genotype-by-environment interactions. The GGE model function from the GGE Biplots package was applied to the genotype-by-environment data matrix [39]. Subsequently, the "Which-Won" function was used to generate the "which-won-where" view of the GGE biplot. The Env relationship function in the same package was utilized to plot environmental relationships, the Disc Rep function was used to assess discrimination ability and representativeness, and the Mean Stability function was applied to create the discrimination ability and representativeness view and stability plots.

AMMI analysis was carried out using the AMMI function in the agricolae package [40].Genotype stability was further assessed using additional stability parameters, including the coefficient of variation (CV) [41] Shukla's stability variance [35], AMMI Stability Value (ASV) [42], ecovalence (Ecoval) [43], and deviation from joint regression analysis (Sij)[44].These parameters were estimated using the metan package in R [45].

The ASV measures the stability of genotypes based on their interactions with the environment. Lower ASV values indicate more stable genotypes.
AMMI stability value was calculated as follows:

$$ASV = \sqrt{[(\text{SSIPCA1} / \text{SSIPCA2}) (\text{IPCA1 score})]2 + (\text{IPCA2 score})2}$$

Where; SSIPCA1 and SSIPCA2 are the sums of the squares of the first and second principal component axes (IPCA), respectively. The IPCA 1 and IPCA2 scores are the interaction scores for the first and second IPCAs, respectively.

GGE biplot analysis is a graphical method that evaluates genotype performance and stability across environments by combining genotype and genotype-environment interaction effects, helping to identify the best and most adaptable genotypes.

$Yij - \mu - \beta j = \sum_{k=1}^{n} \lambda k \xi ik \eta jk + \varepsilon ij$

Where:

- Yij is the grain yield of genotype i in environment j,
- μ is the grand mean,
- βjis the main effect of environmentj,
- λk is the singular value for principal component k,
- ξik andηjk are the genotype and environment scores for PCk, respectively,
- εij is the residual.

3. Results and Discussion

3.1. Combined analysis of Variance (ANOVA)

The analysis of variance revealed highly significant differences ($P < 0.01$) among varieties for the traits considered in the experiment (Table 3). Locations also exhibited highly significant difference ($p<0.01$) for days to heading, tiller number, spike length, number of seeds per spikelets, plant height, thousand seed weight, biomass yield, grain yield, and harvest index. Additionally, varieties demonstrated highly significant variation ($P < 0.01$) among all evaluated traits. The year showed a highly significant effect ($p<0.01$) on plant height, spike length, biomass yield, grain yield, and thousand seed weight.

The interaction between variety and location (V x L) was highly significant ($P < 0.01$) for all traits tested in the durum wheat varieties. Similarly, the interaction between variety and year (V x Y) was highly significant ($p < 0.01$) for plant height, number of grains per spike, grain yield, and harvest index. The interaction between location and year (L x Y) was also highly significant ($p < 0.01$) for all tested traits except for the number of seeds per spikelet and biomass yield.

The three-way interaction effect of variety, location, and year (V x L x Y) was highly significant ($p < 0.01$) for days to heading, plant height, tiller number, spike length, number of seeds per spikelets, thousand seed weight, biomass yield, grain yield, and harvest index. The main effect of

location by year (LxY) had highly significant ($p < 0.01$) effect on days to heading, maturity day, plant height, and grain yield. However, non significant effect was observed for the number of seeds per spikelet and biomass yield. The interaction effect of location by year had a highly significant ($P < 0.01$) effect on days to heading, maturity day, plant height, and grain yield, however, non significant effect was observed for the number of seeds per spikelet and biomass yield. As shown in Table 3, the interaction effect of location and variety had a significant ($P < 0.05$) effect on all evaluated traits. The interaction effect of variety by year was highly significant ($P < 0.01$) for plant height, number of seeds per spikelet, biomass yield, grain yield, and spike length. Conversely, a non-significant effect was observed for maturity date and days to heading. Overall, the combined analysis revealed significant variations among the nine durum wheat varieties for most traits. These findings are consistent with previous studies by [46], which reported significant differences ($p < 0.05$) among durum wheat genotypes across all individual test locations over multiple years.

Days to heading, plant height, tiller number, biomass yield, grain yield, and harvest index were all highly significant ($p \leq 0.01$), as shown in Table 3. However, maturity day and the number of seeds per spike were not influenced by the interaction effects of year, variety, and location. In line with the present results, previous studies have reported highly significant differences ($p \leq 0.01$) [47]. [48] also identified significant genotype-by- location interactions, with highly significant differences ($p \leq 0.01$) among food barely varieties for days to maturity and grain yield. The observed significant GEI interaction indicate inconsistent performance across the tested environments and highlight the varying discriminative abilities of these environments. This suggests the possibility to identify high-yielding and stable genotypes. A particular crop variety may perform well in one environment but poorly in another due to variations in climate, soil conditions, or management practices.

The results of this study are consistent with previous research by [49], which reported significant difference ($P < 0.05$) among bread wheat genotypes for grain yield at all individual test locations in multi-environment trials.

Table 3. Mean squares ofthe two years (cropping season) combined ANOVA results of Eleven quantitative traits from three locations

**SOV**	Mean squares									
	**DH**	**DM**	**PH**	**TN**	**SL**	**NSPS**	**TSW**	**BY**	**GY**	**HI**
Genotypes (G)	345.28**	789.84**	585.36**	1.36**	11.475**	34.85**	226.28**	5.99**	2.07**	86.20**
Location (L)	6.968^{ns}	194.93^{ns}	41.61**	47.51**	43.41**	24.83**	234.07**	25.21**	4.319**	165.47**
Year (Y)	0.964^{ns}	89.92^{ns}	164.36**	0.03^{ns}	0.30^{ns}	2.45^{ns}	27.16**	3.43**	4.200**	1.05ns
V x L	42.69*	187.61*	222.18**	2.02**	11.54**	23.51**	120.06**	2.49**	0.511**	54.12**
V x Y	39.36^{ns}	95.88^{ns}	37.53**	0.59**	1.29**	7.30**	22.20**	0.42ns	0.98**	13.19ns
L x Y	289.05**	496.186*	173.19**	1.49**	2.57**	3.25^{ns}	95.82**	0.750ns	2.003**	59.46**
L x rep	33.73^{ns}	188.16^{ns}	6.47^{ns}	0.22^{ns}	1.36**	5.10^{ns}	12.34**	0.63ns	0.037ns	27.26ns
V x L x Y	53.39**	152.547^{ns}	33.32**	0.33**	1.35**	3.43^{ns}	18.86**	1.015**	0.859**	53.28**
Residuals	22.59	103.58	10.45	0.12	0.33	2.74	2.53	0.41	0.02	14.35

Where SOV: source of variation; **: highly significant at $P < 0.01$; *: significant at $P < 0.05$; ns: non-significant; DF: Degrees of freedom; DH: days to heading; DM: days to maturity; PH: plant height; TN: tiller number; SL: Spike length; NSPS: number of spikelets per spike; BY: biomass yield; NSS: number of seed per spike, TSW: thawed seed weight, GY: grain yield; and HI: harvest index.

3.2. Performance of the Genotype

The grain yield performance of the durum wheat varieties showed significant variability across the tested locations. Tesfaye (G4) recorded the highest grain yield 3.74 t ha^{-1}, indicating its superior adaptability and productivity under the test conditions. It was followed by Don Matteo (G2) which achieved a yield of 3.62 t ha^{-1}, also demonstating strong performance. In contrast, Wehabit (G5) and Rigeat (G6) exhibited the lowest grain yields, at 2.75 t ha^{-1} and 2.78 t ha^{-1}, respectively, suggesting their limited potential for high productivity in these environments.

The differences in grain yield among the genotypes can be attributed to variations in key yield components such as tiller number, spike length, and thousand seed weight. These findings underscore the importance of selecting high-yielding genotypes, such as Tesfaye (G4) to enhance durum wheat production in the study area. Our results are consistent with those of [50] and [51], who found that durum wheat genotypes varied significantly in terms of adaptation and grain yield across Ethiopian highlands. Tesfaye and Don Matteo, in particular were found to be promising genotypes that consistently performed well in a variety of environmental settings. Similar to the current study, [52] observed that cultivars like Wehabit showed reduced grain yield stability. While Wehabit and Rigeat's subpar performance highlights the necessity for location-specific recommendations, Tesfaye's improved performance across locales demonstrates its appropriateness for wider adaption.

The observed variability in grain yield reflects the influenced GEI interaction. High-yielding genotype, such as Tesfaye (G4) 3.74 t ha^{-1} demonstrate better adaptability and efficient resource utilization , whereas low-yielding genotype like Wehabit (G5) 2.75 t ha^{-1} show poor adaptability to the tested environments (Table 4). This result further supports the importance of evaluating durum wheat genotypes across multiple environments to identify stable and high-yielding varieties. Selecting adaptable varieties like Tesfaye (G4) will be vital to enhance durum wheat production and support food security goals in Ethiopia.

Table 4. Mean grain yield and yield-related traits of durum wheat genotypes tested at three locations for two years

Genotypes	DH	DM	PH	TN	SL	NSPS	TSW	BY	GY	HI
1	62.00^{b}	104.44^{bcd}	87.17^{cd}	3.81^{cd}	7.62^{d}	19.43^{a}	35.47^{c}	10.47^{b}	3.3^{c}	26.74^{e}
2	61.22^{b}	110.72^{ab}	98.01^{a}	4.01^{abc}	9.83^{a}	19.39^{a}	36.99^{b}	11.08^{a}	3.62^{b}	30.81^{abc}
3	53.44^{c}	93.33^{f}	88.29^{c}	3.45^{e}	7.69^{d}	16.36^{c}	34.46^{cd}	9.64^{c}	3.09^{d}	31.25^{ab}
4	70.72^{a}	113.39^{a}	94.23^{b}	4.18^{a}	9.06^{b}	19.36^{a}	41.23^{a}	10.94^{a}	3.74^{a}	33.17^{a}
5	62.11^{b}	106.13^{bc}	83.55^{e}	4.11^{ab}	8.47^{c}	19.16^{a}	31.44^{f}	9.40^{c}	2.75^{f}	28.37^{cde}
6	61.44^{b}	101.78^{cde}	78.53^{f}	3.9^{bc}	8.1^{c}	16.64^{bc}	31.12^{f}	9.64^{c}	2.78^{f}	27.77^{de}
7	63.39^{b}	95.23^{ef}	87.02^{cd}	3.55^{e}	7.47^{d}	16.42^{c}	29.5^{g}	10.08^{b}	2.95^{e}	28.76^{bcde}
8	62.89^{b}	102.61^{cd}	85.80^{d}	4.19^{a}	8.29^{c}	17.66^{b}	33.68^{de}	10.15^{b}	3.12^{d}	29.65^{bcd}
9	63.06^{b}	98.97^{def}	85.36^{de}	3.66^{de}	7.5^{d}	16.89^{bc}	32.63^{e}	10.18^{b}	3.15^{d}	26.63^{e}
MSE	22.59	103.58	10.45	0.12	0.33	2.74	2.53	0.41	0.02	14.35
Mean	62.25	102.96	87.55	3.87	8.22	17.92	34.06	10.18	3.17	29.24
CV	7.64	9.88	3.69	9.04	7.03	9.23	4.67	6.27	4.88	12.95
LSD	1.81	3.88	1.23	0.13	0.22	0.63	0.61	0.24	0.06	1.45

Where: MSE= Mean sequar of error LSD= List Significant Deference and CV = Cofficent of varation.

3.3. Genotype performance at each tested location.

The mean grain yield of the nine durum wheat genotypes across six environments (E1–E6) revealed notable GEI interaction, highlighting the differential performance of the genotypes under varying conditions (Table 5). Tesfaye (G4) consistently exhibited superior performance, achieving the highest mean grain yield (3.74 t ha^{-1}), driven by its exceptional adaptability in environments E1 (3.97 t ha^{-1}), E4 (3.97 t ha^{-1}), and E6 (4.5 t ha^{-1}). This indicates that Tesfaye is particularly well-suited to a range of environmental conditions. Don Matteo (G2) ranked second in overall performance with a grain yield of (3.62 t ha^{-1}), excelling in E2 (4.7 t ha^{-1}) and E4 (4.13 t ha$^{-1)}$, suggesting its suitability for high-yielding environments, especially in Simada and Bichena. In contrast, Wehabit (G5) and Rigeat (G6) recorded the lowest mean grain yield, at 2.75 t ha^{-1} and 2.78 t ha$^{-1,}$ respectively. Their consistently poor performance across most environments suggests limited adaptability or a higher sensitivity to environmental variations, making them less reliable for achieving high yields. Similarly, Alemtena (G3)**,** despite excelling in E3 (4.82 t ha^{-1}), had a lower overall mean yield (3.08 t ha^{-1}) due to inconsistent performance in other environments, indicating that its high yield potential is not consistent across all conditions. Previous studies in Ethiopia support these findings. For instance, Tesfaye and Don Matteo were identified as stable and high-yielding durum wheat varieties across several northern Ethiopian locations [22, 53]. Similarly, [54] reported that Tesfaye demonstrated broad adaptability and consistent yield performance in both highland and moisture-stressed areas. Conversely, [54] found that Wehabit exhibited poor stability across diverse environments, aligning with our results on its limited performance.

Environmental effects were evident, with Dabat 2023 (E6) producing the highest mean grain yield (3.767 t ha^{-1}), followed by Bichena 2022 (E1) and Bichena 2023 (E4), which recorded mean grain yielda of 3.256 t ha $^{-1}$and 3.191 t ha^{-1}, respectively. Conversely, Simada 2022 (E2) had the lowest mean yield (2.695 t ha^{-1}), likely due to less favorable growing conditions in that region. These results highlight the importance for evaluating genotypes across diverse environments to identify high-yielding and stable performers. Genotypes such as Tesfaye (G4) and Don Matteo (G2) should be prioritized for large-scale production in the Amhara region and similar agro-ecologies, given their demonstrated adaptability and productivity. These findings

contribute to the growing body of evidence supporting the use of multi-environment trials and stability analysis to guide durum wheat variety recommendations in Ethiopia.

Table 5. Mean grain yield (t ha^{-1}) of nine durum wheat genotypes across six environments (location and year combinations)

Genotypes	(E1)	(E4)	(E3)	(E6)	(E2)	(E5)	Mean
**1**	3.53b	3.66b	2.57d	3.67b	3.13a	3.2b	3.29
**2**	3.84a	4.13a	2.6d	4.7a	3.17a	3.26ab	3.62
3	2.74c	2.91c	4.82a	2.82c	2.41cd	2.8c	3.08
**4**	3.97a	3.97a	3.23b	4.5a	3.23a	3.53a	3.74
5	2.87c	2.79cd	2.75cd	2.83c	2.5bc	2.77c	2.75
6	2.7c	2.63d	2.92c	3.63b	2.29d	2.53c	2.78
7	2.85c	2.85cd	3.18b	3.41b	2.61b	2.8c	2.95
8	3.97a	2.91c	2.92c	3.63b	2.5bc	2.77c	3.12
9	2.85c	2.85cd	2.57d	4.7a	2.41cd	3.53a	3.15
MSE	0.03	0.023	0.015	0.031	0.007	0.03	
Mean	3.256	3.191	3.062	3.767	2.695	3.02	3.16
CV	5.314	4.773	4.035	4.642	3.125	5.62	
LSD	0.299	0.264	0.214	0.303	0.146	0.29	

Where: E1=Bichena 2022, E2=Simada 2022,E3=Dabat 2022=E4=Bichena 2023=E5=Simada 2023, E6=Dabat 2023, MSE= Mean sequar of error and CV = Cofficent of varation.

3.4. Additive Main Effects and Multiplicative Interaction (AMMI) Analysis

Additive Main effect and Multiplicative Interaction (AMMI) analysis of variance for grain yield of the nine durum wheat genotypes evaluated across six environments is presented in (Table 6). The AMMI analysis revealed genetic variation and potential for selecting stable genotypes. The results demonstrated that the variation among E, G, and GEI was highly significant ($P < 0.01$). Sum squares (SS) partitioning revealed that the environmental effect was the largest contributor to variation followed by the GEI interaction and genotype main effect. Since changes in G and G × E are ordinarily smaller, the environment often explains most of the differences in genotype performance [55].

Furthermore, the AMMI model's first two principal component axes (IPCAs) were found to be highly significant ($P < 0.01$) when used for GEI partitioning. The AMMI, which includes IPCA1 and IPCA2, is the best prediction model for cross-validating yield variation explained by the

genotype-environment interaction [56, 57]. Variations in the grain yield components from the AMMI indicated that genotype, environment, and genotype by environment interaction (GEI) all had significant ($P < 0.01$) effects, explaining variability in both environments and genotypes, and that there are possibilities for the selection of genotypes that are high-yielding, stable, and well-performing. G8 (Mangudo) was the most stable but only modestly productive, according to the AMMI1 biplot, while G4 (Tesfaye) and G2 (Don Matteo) combined high yield with good stability. Although they demonstrated strong interactions with particular surroundings, G3 (Alemtena) and G6 (Rigeat) were less stable. In contrast to E3 (Dabat 2022), which was highly interactive and showed significant environmental influence, E6 (Dabat 2023) was both stable and high yielding.

Grain yield was significantly impacted by environments, genotypes, and their interactions, according to AMMI analysis ($P < 0.001$). The sum of squares resulting from the GEI is further separated into five important Interaction Principal Components (PC-1 to PC-5) in consideration of the multiplicative component of AMMI (Table 6). The highest proportion of the total sum of squares was accounted for by the environment (45.96%), followed by the genotype by environment interaction (25.86%) and then genotype (25.2) (Table 6). According to Gauch (2006), this is consistent with the outcome anticipated from GEI analysis. The environment-related sum of squares' maximum magnitude indicates that the test conditions vary and have an impact on how the grain yield characteristic manifests itself. In line with findings from barley [58] and durum wheat grain yield [59] the GEI and the largest percentage of environmental variance are in accord. The higher sum of squares due to the genotype-by-environment interaction compared to the genotype effect indicates the presence of mega-environments (**Fig. 1**). Based on IPCA1 and IPCA2, Tesfaye, Fetan, and Don Matteo showed small interaction values with high yields, indicating wide adaptability and stability, while Alemtena had large IPCA scores, reflecting poor stability. Mangudo and Rigeat showed moderate adaptability. The findings reveal that the various genotypes reacted differently to the environmental conditions, highlighting the phenotypic diversity among the evaluated genotypes. This also implies the potential presence of distinct mega-environments, each with genotypes that exhibit varying performance levels.

Table 6. AMMI analysis of variance for grain yield of nine durum wheat genotypes

Source	DF	Sum square	Mean square	Proportion	Accumulated
ENV	5	29.78	3.36**	45.22%	NA
REP(ENV)	12	0.49	0.04**	0.74%	NA
GEN	9	16.6	2.08**	25.2%	NA
GEN: ENV	40	16.84	0.75**	25.86%	NA
PC1	12	20.72	1.72**	69.6	69.6
PC2	10	5.62	0.56**	18.9	88.5
PC2	8	2.11	0.26**	7.1	95.6
PC4	6	1.11	0.19**	3.7	99.3
PC5	4	0.22	0.06**	0.7	100
Residuals	96	2.15	0.02	NA	NA
Total	201	65.86	0.47	NA	NA

Genotype rank based on AMMI stability value

Number	Genotype	Mean yield	Rank	ASTAB	ASTAB	Shukula	bij	ASI	IPCA 1	IPCA 2	ASV	Rank
1	Fetan	3.295	3	3.89	5	1.23	8.36	2.76	-0.415	0.2910	1.38	7
2	Don Matteo	3.51	2	5.78	6	2.22	1.41	4.00	-0.609	0.2569	1.99	8
3	Alemtena	3.01	6	1.43	9	7.42	-1.65	7.56	0.161	0.1107	3.77	9
4	Tesfaye	3.69	1	1.41	1	2.49	1.20	1.74	-0.264	0.1547	8.73	5

5	Wehabit	2.75	9	2.93	3	3.37	3.46	1.07	0.1521	0.2138	5.38	2
6	Rigeat	2.78	8	3.64	4	5.04	1.31	1.11	0.1366	0.3365	5.57	3
7	Utuba	2.94	7	1.76	2	2.60	7.32	1.74	0.2651	-0.1175	8.69	4
8	Mangudo	3.11	4	7.53	7	1.50	1.42	9.67	0.1166	0.2995	4.83	1
9	Toltu	3.07	5	9.41	8	2.74	1.73	2.66	-0.3087	-0.8727	1.32	6

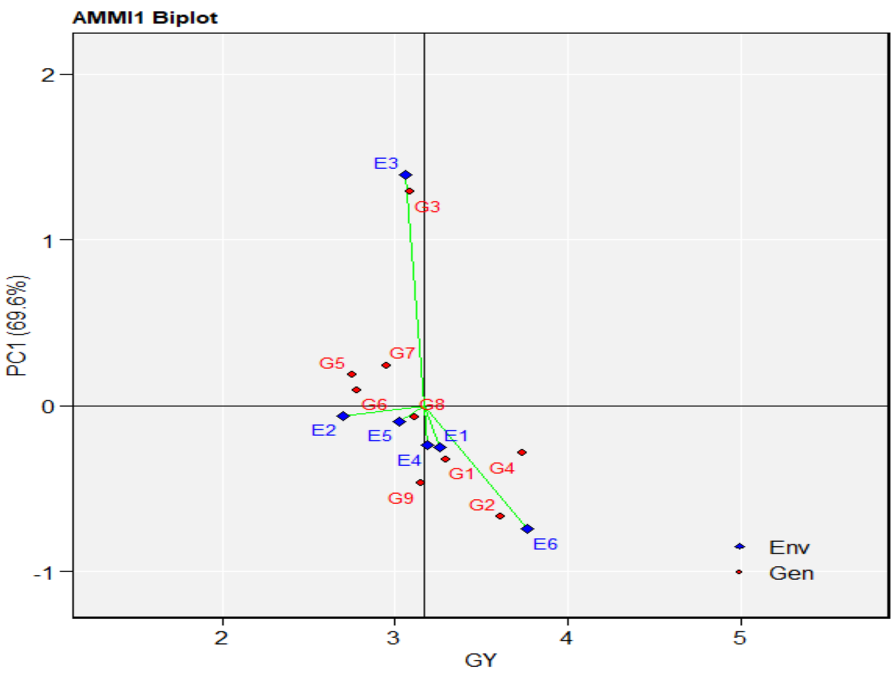

Figure 1. AMMI Analysis model biplot for grain yield of durum wheat genotypes evaluated at six environments. Where: G1: Fetan,G2: Don Matteo, G3: Alemtena, G4: Tesfaye, G5: Wehabit, G6: Rigeat, G7: Utuba, G8:Mangudo, and G9: Toltu genotype (E1:

Simada, E2: Bichena, E3: Dabat 2022 cropping season and E4: Simada, E5: Bichena, E6: Dabat in 2023 cropping season. The red numbers connected to the polygon represent the genotypes, whereas the blue numbers represent the locations/test environments.

3.5. Stability Analysis and Mega-Environment Classification Using GGE Biplot.

3.5.1. GEI analysis for which-won-where patterns using GGE biplot

The GGE results were similar to those obtained from AMMI and combined ANOVA. The polygon view of the GGE biplot highlights the top-performing genotype in each environment, visually representing key concepts such as crossover interaction, mega-environment differentiation, and specific adaptation [60]. Mega environment analysis refers to dividing a crop-growing region into distinct target zones [61].

In this analysis, six vertex genotypes were identified: G3 (Alemtena), G4 (Tesfaye), G2 (Don Matteo), G5 (Wehabit), G6 (Rigeat), and G9 (Toltu). The biplot was divided into six sections by using six rays, with environments distributed across three of these sections while genotypes appeared in all. Each sector's vertex genotype exhibited the highest grain yield for the environments within that sector, indicating specific adaptation to particular mega-environments.

The GGE biplot analysis identified two distinct wheat-growing mega-environments.The first mega-environment comprised environments E1 (Bichena 2022), E2 (Simada 2022), E4 (Bichena 2023), E5 (Simada 2023), and E6 (Dabat 2023), with G2 identified as the winning genotype. The second mega-environment consisted solely of E3 (Dabat 2022), where G3 was the top-performing genotype. No mega-environments were associated with sectors where genotypes G5, G6, and G9 were vertex genotypes, indicating that these genotypes were not the best performers in any tested environment. G8, located near the origin, exhibited average performance with wide adaptability but moderat grain yield. The GGE biplot explained 82.67% of the total G×E variation (Axis 1: 61.48%, Axis 2: 21.19%). Previous studies by [62] have reported similar findings, suggesting that an ideal genotype should have high mean performance and stability. The genotypes that performed the best or worst in specific environments were the furthest from the biplot's origin (Bekana, 2023), which were more sensitive to environmental changes, and regarded as specifically adapted genotypes. The GGE biplot polygon view helps identify the most ideal test environments within each sector, which are crucial for selecting superior genotypes [63]. Therefore, G4 (Tesfaye) had the highest yield in Bichena and Simada 2022, G9

(Toltu) had the highest yield in Simada and Dabat, and G3 (Alemtena) had the highest yield in Dabat 2022. The most effective method for identifying superior genotypes and visualizing the patterns of interaction between genotypes and environments in data analysis is the polygon view of the GGE biplot, which helps to estimate the potential existence of various mega-environments [64, 65]. Vertex genotype G4 falls in the first and second quadrants, G9 falls in the third quadrant whereas G3 is located in the second and fourth quadrants. Therefore, the six testing environments were categorized into three mega environments, while the nine genotypes were divided into six genotypic groups (**Fig. 2**).

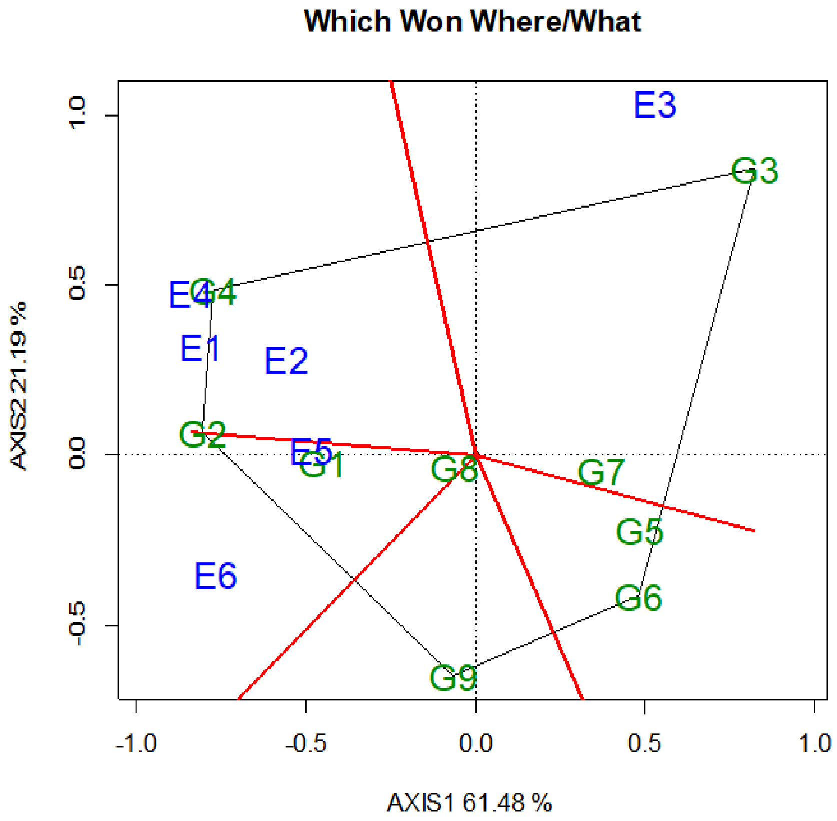

Figure 2**.** Which-won-where view of GGE biplot for grain yield of durum wheat genotype Where: G1: Fetan, G2: Don Matteo, G3: Alemtena, G4: Tesfaye, G5: Wehabit, G6: Rigeat, G7: Utuba, G8:Mangudo, and G9: Toltu genotype (E1: Simada, E2: Bichena, E3: Dabat 2022 cropping season and E4: Simada, E5: Bichena, E6: Dabat in 2023 cropping season. The blue numbers connected to the polygon represent the genotypes, whereas the green numbers represent the locations/test environments.

3.5.2. Evaluation of environments relative to the ideal environment

Through the representation of the center of the concentric circles in a polyg on perspective, the discrimination and representativeness of the GGE biplot often illustrate the ranking of an ideal test environment for a particular genotype or for all genotypes (**Fig. 3**). A point located on the average environment axis in the positive direction,with the longest vector from the biplot origin is considered the most representative and thus the optimal test environment in the GGE biplot [60]. The test environment that is both most representational of the target environment and most discriminating (informative) is deemed optimal. The ideal test environment is represented by the center of the concentric circles (**Fig. 3**). E5 and E2 performed the worst in selecting genotypes adapted to the entire region, whereas E1, E4, and E6 were closest to the ideal environment point and, therefore, best represented the Durum Wheat production environments.

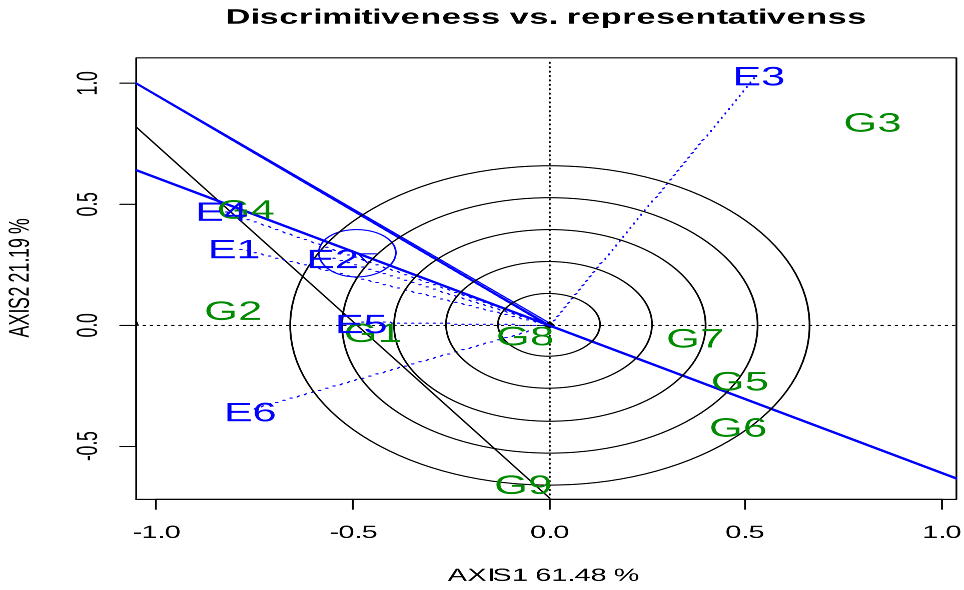

Figure 3. Discrimination vs. representativeness of genotypes. Where: E1: Simada, E2: Bichena, E3: Dabat 2022 cropping season and E4: Simada, E5: Bichena, E6: Dabat in 2023 cropping season.

3.5.3. Evaluation of genotypes relative to the ideal genotype

The ideal genotype for durum wheat production is positioned at the center of the concentric circles, in the GGE biplot symbolizing optimal traits characterized by high mean yield and stability. In this context, Tesfaye, stands out desirable genotype due to its exceptional yield performance and stable characteristics, making it highly suitable for grain production. Genotypes

that are close to the ideal genotype, such as Fetan (G1), Utuba (G7), and Wehabit (G5), are also recognized for their favorable performance and stability, aligning closely with the traits of the optimal genotype. These genotypes are therefore considered strong candidates for cultivation.

In contrast, genotypes positioned further from the ideal center specifically Don Matteo (G2), Toltu (G9), and Alemtena (G3) are considered less desirable due to their low yield performance (**fig.4**). G8 (Mangudo) was the most stable but only modestly productive, according to the grain yield men values and stability figure. This classification is supported by data from the mean separation test referenced in fig 4, which corroborates these observations regarding yield stability and performance. Overall, the analysis highlights the value of selecting genotypes based on their proximity to the ideal genotype as depicted in the concentric circle model, of GGE biplot. This approach effectively illustrates the relationship between yield performance and genetic stability in durum wheat breeding programs. Supporting this finding, Mulugeta, Tesfaye [22] identified variety G-169 as the most ideal genotype for durum wheat grain production. G4 as the ideal genotype and E4 as the ideal environment. G1 and G2 also appear as desirable genotypes, while E1 and E2 emerge as desirable environments, suggesting they offer favorable conditions for good performance and stability (**fig. 4).** Additionally, Multivariate analyses of Ethiopian durum wheat revealed genotypes that are both high-yielding and stable across trials. The relative contributions of stability and grain yield for identifying desirable genotypes found in this study, using the ideal genotype procedure of the GGE biplot, were also similar to those reported by Adem Wado [66] genotype × location × planting pattern interaction and stability analysis of sorghum (Sorghum bicolor (L.) Moench) varieties in the drylands of Ethiopia.

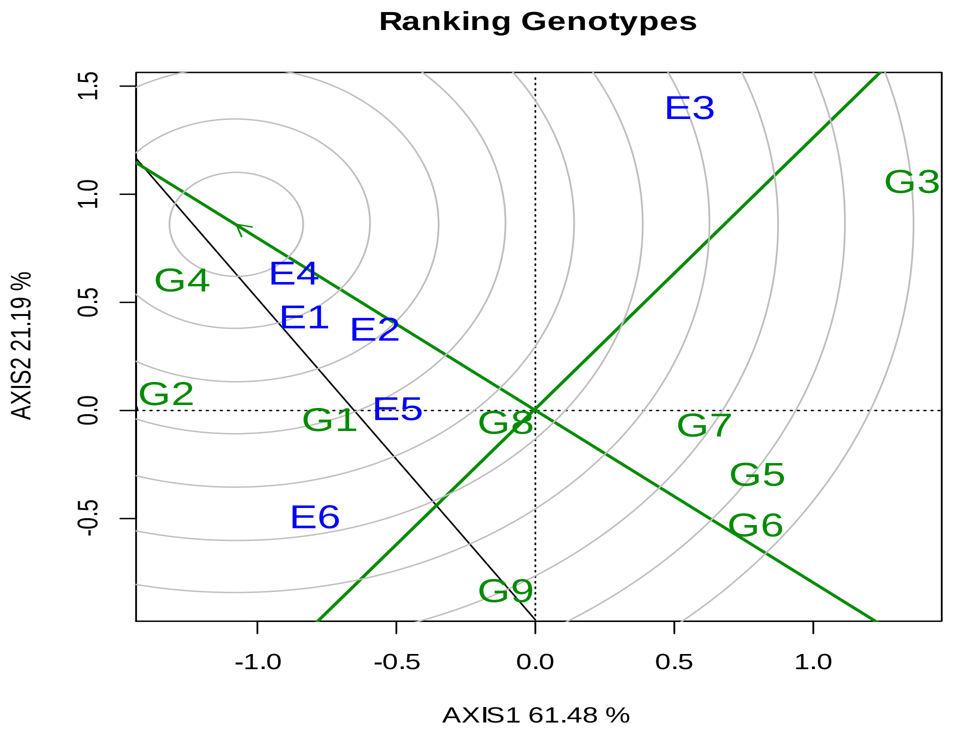

Figure 4. Ranking of genotypes based on yield performance and stability. Where: G1: Fetan, G2: Don Matteo, G3: Alemtena, G4: Tesfaye, G5: Wehabit, G6: Rigeat, G7: Utuba, G8:Mangudo, and G9: Toltu genotype (E1: Simada, E2: Bichena, E3: Dabat 2022 cropping season and E4: Simada, E5: Bichena, E6: Dabat in 2023 cropping season. The blue numbers connected to the polygon represent the genotypes, whereas the green numbers represent the locations/test environments.

3.5.4. Interrelationship among environment

In a GGE biplot, the correlation intensity between enviroment is represented by the cosine of the angle between any two environment vectors [60]. A correlation is considered positive if the angle is less than 90°, negative if it is greater than 90°, and nonexistent if it is around 90°. According to [67] and [55] there is a significant connection between the correlation coefficient of two environments and the angle between their vectors, which is utilized to categorize the test environments. Therefore, the top-yielding genotypes in one environment will perform best in other environments if two environments have a positive correlation. In the context of GGE biplot analysis,understanding the relationship between environments is crucial for interpreting genotype performance across different conditions. When two environments show a negative correlation, it suggests that genotypes performing well in one are likely to perform poorly in the other, and vice versa [55, 60]. In the present study, as shown in (**Fig. 5**), the environments Simada, and Bichena are positively correlated, as indicated by an angle their vectors less than 90°. On the other hand, the angle between Dabat and simada greater than 90°, indicating a negative correlationbetween these two environments.

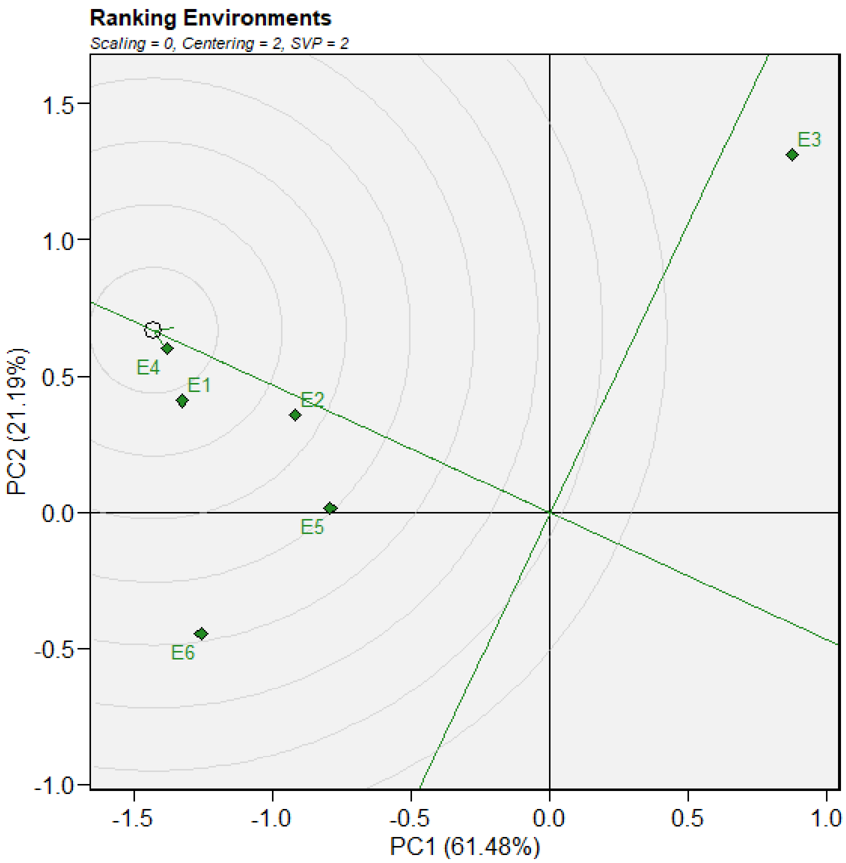

Figure 5. Ranking of environments relative to the ideal environments

Where: E1: Simada, E2: Bichena, E3: Dabat 2022 cropping season and E4: Simada, E5: Bichena, E6: Dabat in 2023 cropping season.

3.6. Stability of durum wheat genotypes

Genotypes' mean grain yield and stability performance are graphically represented using the average environment coordination (AEC) method (**Fig.6**). Combining grain yield with genotype stability performance can help identify the genotypes that yield the highest and most stable results [68]. The genotype with the highest mean performance and greatest stability across all test environments is considered ideal [69, 70]. According to the AEC view in the comparison biplot, a desirable genotype are those located close to the ideal genotype, which is typically positioned at the center of the concentric circles or at the tip of the arrow indicating the ideal point. In the AEC (Average Environment Coordination) system, the mean performance of genotypes is represented by an arrow along the AEC X-axis (PC1), which passes through the origin of the biplot and indicates the average environment axis, reflecting the overall

performance of genotypes across environments.This graphical representation is instrumental in visually distinguishing high-performing, stable genotypes from those with variable or suboptimal performance.

On the positive side of the AEC abscissa, genotypes located closer to the origin would have a higher mean grain yield. In contrast, genotypes farther away on the negative side would have a lower mean grain yield. Additionally, a genotype's projection becomes less stable the farther it is from the origin in absolute terms. This line indicates a higher mean yield across the environments. Hence, in the present study, G2 gave the highest mean yield, followed by G1, and G6. The performance of the remaining genotype groups (G3 and G5) was below the grand mean yield (**Fig. 6**). An ideal genotype is completely stable across a wide range of environments and has the highest average performance of any genotype [71].

Higher GEI interaction and lower stability are indicated by genotype that lie further from the origin along the AEC ordinate (y-axis), in either direction [72]. G4 (Tesfaye) and G2 (Don Matteo) showed high yield and stability. G8 (Mangudo) was the most stable but moderate in yield. Conversely, G3 (Alemtena) and G6 (Rigeat) showed instability and were adapted to specific environments. Although G3 (Alemtena) produced the highest mean yield in some environments, its large deviations along the AEC ordinate indicated low stability (**Fig.6**). Among environments, E2 (Simada 2022), E4 (Bichena 2023), and E1 (Bichena 2022) were located closer to the AEC abscissa, indicating representativeness, while E3 (Dabat 2022) showed the largest deviation, suggesting high discriminating ability but low representativeness.

An ideal genotype for a specific environment has the highest mean yield and responds best in that particular environment while being less stable in the other environments, thus necessitating recommendations for the specific environment [73]. Stability is reported to have lower heritability than mean performance [74, 75]. It is important to note that stability pertains to the genotype's relative performance and gains significance only when linked to the mean [76]. In summary, environmental factors (e.g., temperature, rainfall, soil quality) and genetic factors (e.g., drought resistance, nutrient efficiency) jointly influence yield-related parameters such as biomass, spike length, and the number of tillers. Wheat varieties with favorable genetic factors tend to perform better under various environmental conditions, resulting in improved overall

yield [11, 77, 78]. Genotype stability was further evaluated using different stability parameters. The figure clearly identifies G4 as the ideal genotype and E4 as the ideal environment. G1 and G2 also appear as desirable genotypes, while E1 and E2 emerge as desirable environments, suggesting they offer favorable conditions for good performance and stability.

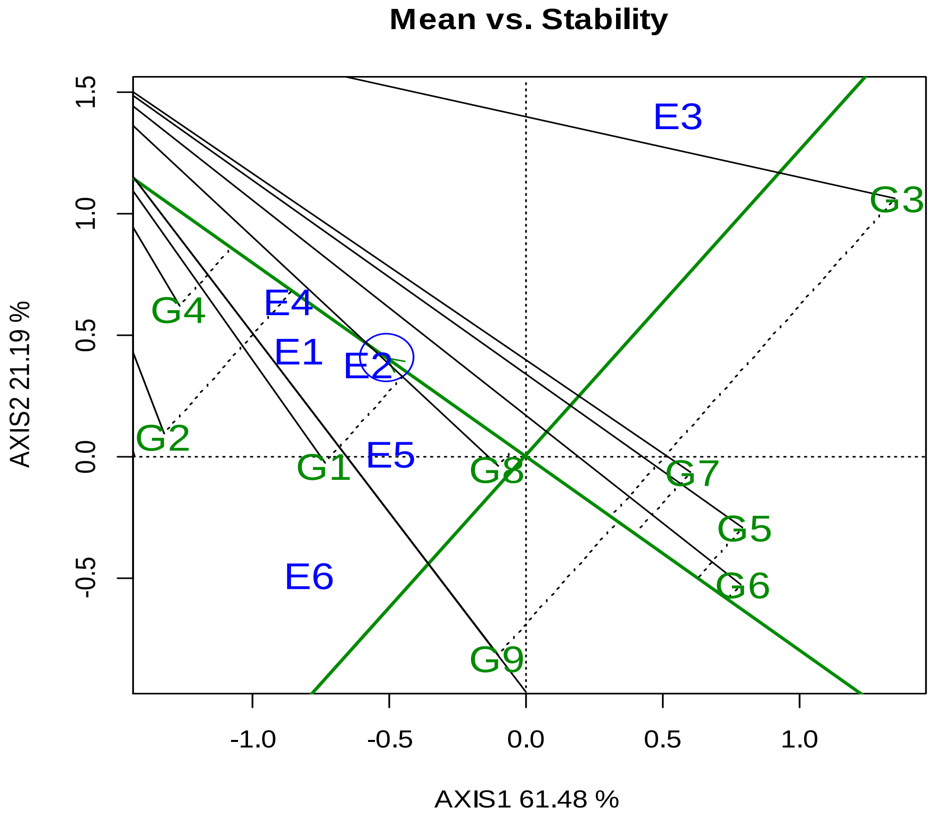

Figure 6.Which genotype was more stable across the tested environments? Where: G1: Fetan, G2: Don Matteo, G3: Alemtena, G4: Tesfaye, G5: Wehabit, G6: Rigeat, G7: Utuba, G8:Mangudo, and G9: Toltu genotype (E1: Simada, E2: Bichena, E3: Dabat 2022 cropping season and E4: Simada, E5: Bichena, E6: Dabat in 2023 cropping season. The blue numbers connected to the polygon represent the genotypes, whereas the green numbers represent the locations/test environments.

3.7. Stability parameter

3.7.1. The regression coefficient

The performance of a genotype in an environment is determined by its mean performance, environmental responsiveness as a linear function, and its deviation from regression. Eberhart and Russell suggested using a linear regression coefficient and the variance of the regression

deviations to evaluate crop responses to environmental changes. The average stability is indicated by a regression coefficient (bi) that approaches one and a deviation from regression (S2di) of zero. In this model, regression scores above one indicate varieties that are more sensitive to changes in the environment (below-average stability) and are specifically adaptable to high-yielding environments [79]. Conversely, regression coefficients below one enhance the specificity of adaptability to low-yielding environments by demonstrating greater resistance to environmental change (above-average stability).

The present results indicated that linear regression of the average grain yield of individual genotype on the average yield of all varieties in each environment produced regression coefficients (bi values) ranging from -0.012 to 1.73. This variation in regression coefficients reflects differing responses of the varieties to environmental changes (Table 7). A mean regression coefficient (bi) that is close to one, low deviation form regression, and grain yields higher than the grand mean indicate that the aforementioned varieties Fetan, Tesfaye and Utuba are well adapted to all environments, indicating broader adaptation across different environments. In contrast Toltu are poorly adapted to all environments. It is recommended that these varieties be cultivated under unfavorable conditions, as they demonstrate resilience to environmental variations. Similar finding have been reported by other researchers [80].

Table 7. The various models of stability used to partition the GEI interaction for grain yield in the test of durum wheat genotypes and their ranking

Genotypes	Mean	Rank	Bi	s^2di	σ^2	Wi	Pi	ASU	YSI	Rank	s1	s2	ASI	ASV
1	3.3	3	0.84	0.14	0.12	97.70	0.11	7	10	4	0.27	7.77	0.28	1.38
2	3.51	2	1.41	0.21	0.22	102.00	0.00	8	10	3	0.33	10.70	0.40	2.00
3	3.02	6	-0.012	0.62	0.74	80.30	0.80	9	15	6	0.20	8.67	0.76	3.78
4	3.69	1	0.98	0.04	0.03	114.00	0.00	5	6	1	0.07	1.47	0.18	0.87
5	2.75	9	0.35	0.00	0.03	84.70	0.79	2	11	9	0.40	4.97	0.11	0.54
6	2.78	8	1.32	0.06	0.05	83.30	0.66	3	11	8	0.33	4.57	0.11	0.56
7	2.95	7	0.73	0.04	0.03	90.00	0.56	4	11	7	0.13	3.50	0.17	0.87
8	3.12	4	1.43	0.14	0.20	91.30	0.27	1	5	2	0.27	8.80	0.10	0.48
9	3.07	5	1.73	0.22	0.27	87.40	0.45	6	11	5	0.00	14.60	0.27	1.33

S^1= Nassar and Huens's (1987) Mean of the absolute rank differences of a genotype over the n environments, **S^2**= Nassar and Huens's (1987) variance among the ranks over the k environments, **Wi**=Wriecke's ecovalence, **Pi** =Cultivar superiority, **σ^2** = Shukla's (1972) stability variance, **bi** =Eberhart and Russell (1966) stability value of regression coefficient and **S^2di**= Eberhart and Russell (1966) stability deviation value from regression, ASI= AMMI stability index, and ASV=AMMI stability value.

3.8. Limitation of the Study

While the findings provide valuable insights into the performance and stability of durum wheat genotypes, it is important to acknowledge certain limitations. The study is based on data collected over only two years and across six environments, which may not fully capture the extensive environmental variability affecting durum wheat production. Additionally, although the statistical methods used are robust, they carry inherent assumptions and limitations that could influence the interpretation of genotype-by-environment interactions.Therefore, recommendations for commercial cultivation should be made with caution. Conducting further multi-year and multi-location trials would enhance the reliability and generalizability of these results.

4. Conclusions and Recommendation

Analysis of GEI interaction in this study highlighted the importance of evaluating durum wheat genotypes across multiple environments to determine their yield stability and adaptability. The combined analysis of variance over the years showed significant difference among genotypes, environments, and their interaction, confrining that both genetic and environmental factors significantly influenced grain yield and other agronomic traits. Genotype Tesfaye, followed by Don Matteo, consistently exhibited superior phenotypic performance across all traits,with grain yields of 3.74 and 3.62 tha^{-1}, respectively, making them the top-yielding genotypes. ~~Grain yield across test environments varied substantially, ranging from 4.5 t ha^{-1} in Dabat to 2.29 t ha^{-1} in Simada.The first principal component (PC1) contributed significantly to the interaction, accounting for 69.76%, whereas the second principal component (PC2) explained an additional 21.71%, together accounting for 91.47% of the genotype-by-environment interaction.~~ AMMI and GGE biplot analyses effectively visualized the GEI patterns, grouping the six test environments and nine genotypes into two mega-environments and six genotype clusters. Simada and Bichena emerged as both highly discriminating and representative environments, making them ideal sites for evaluating genotype performance. Grain yield analysis showed that Tesfaye (G4) and Don Matteo (G2) consistently achieved the highest yields and broad adaptability, whereas Wehabit (G5) and Rigeat (G6) performed poorly, reflecting limited stability. ~~AMMI and GGE biplot analyses confirmed significant GEI, with environments explaining most of the variation. AMMI~~

~~identified Tesfaye and Don Matteo as high yielding and stable genotypes, while Alemtena (G3) and Rigeat (G6) showed instability and environment specific adaptation.~~ Based on these results, it is recommended to cultivate Tesfaye and Don Matteo in the Amhara region and other similar durum wheat-growing areas, given their high yield and moderate to high stability. Additionally, Simada and Bichena should be prioritized as core testing sites in future multi-environment trials due to their strong discriminative and representative capacities. Continued exploration of GEI is essential to refine varietal recommendations and ensure optimal wheat production in specific agro-ecological zones.

Conflict of interests

The authors have not declared any conflict of interest.

Authors' contribution statement

A.E: Wrote the original draft, data collected and analyzed, investigated, revised, and edited; **A. A** : Restructured, reviewed, collected the data, and analyzed; **Y.A**: Contributed to reviewing, data collection, and editing, **M.A:** Restructured, Contributed to reviewing, data collection and editing and **K.N:** Restructured, Contributed to reviewing, data collection and editing All authors read and approved the final version of the manuscript.

Ethics approval and consent to participate

Not applicable

Consent for publication

Not applicable

Availability of data and material

Datasets generated in this study are available from the corresponding author upon reasonable request.

Funding statement:

No found source

5. REFERENCES

[1] Tilahun WM, Bekeko Z, Silesh F. IMPORTANCE OF WHEAT STEM RUST DISEASE (Puccinia graminis f. sp. tritici) IN NORTH SHEWA, ETHIOPIA, AND RESISTANCE OF DURUM WHEAT LANDRACE ACCESSIONS [Triticum turgidum subsp. durum (Desf.) Husn.] TO THE PREVAILING RACES OF THE PATHOGEN: Haramaya University; 2022.
[2] Yang F, Zhang J, Liu Q, Liu H, Zhou Y, Yang W, et al. Improvement and re-evolution of tetraploid wheat for global environmental challenge and diversity consumption demand. International journal of molecular sciences. 2022;23:2206.
[3] Vavilov NI. The origin, variation, immunity and breeding of cultivated plants: LWW; 1951.
[4] Kebede M, Temam B, Getahun M, Tsegaye Y. Evaluation of durum wheat (Triticum turgidum) varieties against stem rust (Puccinia graminis f. sp. tritici) in selected districts of southern Ethiopia. 2020.
[5] Hodson D, Jaleta M, Tesfaye K, Yirga C, Beyene H, Kilian A, et al. Ethiopia's transforming wheat landscape: tracking variety use through DNA fingerprinting. Scientific reports. 2020;10:18532.
[6] Baranski M. The wide adaptation of green revolution wheat: Arizona State University; 2015.
[7] Tidiane Sall A, Chiari T, Legesse W, Seid-Ahmed K, Ortiz R, Van Ginkel M, et al. Durum wheat (Triticum durum Desf.): Origin, cultivation and potential expansion in Sub-Saharan Africa. Agronomy. 2019;9:263.
[8] Dibaba R. Wheat production, marketing and consumption in Ethiopia. Journal of Marketing and Consumer Research. 2019;55:10-9.
[9] Shiferaw B, Smale M, Braun H-J, Duveiller E, Reynolds M, Muricho G. Crops that feed the world 10. Past successes and future challenges to the role played by wheat in global food security. Food security. 2013;5:291-317.
[10] Hailu F. Farmers perception of pesticide use and genetic erosion of landraces of tetraploid wheat (Triticum spp.) in Ethiopia. Genetic resources and crop evolution. 2017;64:979-94.
[11] Eskezia A, HK, MA. Grain yield Performance Evaluation of Bread Wheat (Triticum aestivum L.) Varieties in

East Gojjam Zone. Advanced Journal of Interdisciplinary Studies. 2024;8(2):1663-76.
[12] Minot N, Warner J, Lemma S, Kasa L, Gashaw A, Rashid S. The wheat supply chain in Ethiopia: Patterns, trends, and policy options. Gates Open Res. 2019;3:174.
[13] Abdalla A, Stellmacher T, Becker M. Trends and prospects of change in wheat self-sufficiency in Egypt. Agriculture. 2022;13:7.
[14] Cappelli A, Cini E. Challenges and opportunities in wheat flour, pasta, bread, and bakery product production chains: A systematic review of innovations and improvement strategies to increase sustainability, productivity, and product quality. Sustainability. 2021;13:2608.
[15] ESS. Ethiopian Statistical Services). 2022. Report on Area and Production of Major Crops (private peasant holdings, Meher season). Volume I, Statistical Bulletin 593, Addis Ababa, Ethiopia. 2022.
[16] Anteneh A, Asrat D. Wheat production and marketing in Ethiopia: Review study. Cogent Food & Agriculture. 2020;6:1778893.
[17] Badebo A, Gelalcha S, Ammar K, Nachit M, Abdalla O. Overview of durum wheat research in Ethiopia: challenges and prospects. 2009.
[18] Tadesse W, Zegeye H, Debele T, Kassa D, Shiferaw W, Solomon T, et al. Wheat production and breeding in Ethiopia: retrospect and prospects. Crop Breeding, Genetics and Genomics. 2022;4.
[19] Zenebe M. Assessment and Management of Wheat Stem Rust (Puccinia graminis tiritici) Using Fungicides and Wheat Varietiesin Meket District, North Wollo, Ethiopia 2022.
[20] Mekuriaw A. Agroecology-specific varietal matching to achieve self-sufficiency in wheat production in Ethiopia. Heliyon. 2023;9.
[21] Yismaw D. GENOTYPE BY ENVIRONMENT INTERACTION DURUM WHEAT (Triticum turgidum GROWING AREAS OF GONDAR AND NORTH SHOA GRADUATE PROGRAM AND STABILITY ANALYSIS ssp.

durum) GENOTYPES IN ZONES OF AMHARA REGION, ETHIOPIA M. Sc. Thesis By Yismaw Degenet Mogess November Bahir Dar, Ethiopia OF WHEAT 2019 2019.
[22] Mulugeta B, Tesfaye K, Geleta M, Johansson E, Hailesilassie T, Hammenhag C, et al. Multivariate analyses of Ethiopian durum wheat revealed stable and high yielding genotypes. PloS One. 2022;17:e0273008.
[23] Tinker N, Yan W. Information systems for crop performance data. Canadian Journal of Plant Science. 2006;86:647-62.
[24] Yan W, Tinker NA, Bekele WA, Mitchell-Fetch J, Fregeau-Reid J. Theoretical unification and practical integration of conventional methods and genomic selection in plant breeding. Crop Breeding, Genetics and Genomics. 2019;1.
[25] Tesfaye A, Bogale A, Namara RE, Bacha D. The impact of small-scale irrigation on household food security: The case of Filtino and Godino irrigation schemes in Ethiopia. Irrigation and Drainage Systems. 2008;22:145-58.
[26] Kebede G, Worku W, Feyissa F, Jifar H. Genotype by environment interaction and stability analysis for selection of superior fodder yield performing oat (Avena sativa L.) genotypes using GGE biplot in Ethiopia. Ecological Genetics and Genomics. 2023;28:100192.
[27] Mwila C. Grain yield stability, genetic gain and path coefficient analyses in advanced soybean (Glycine max (L.) Merr.) lines 2017.
[28] Wodebo KY, Tolemariam T, Demeke S, Garedew W, Tesfaye T, Zeleke M, et al. AMMI and GGE biplot analyses for mega-environment identification and selection of some high-yielding oat (Avena sativa L.) genotypes for multiple environments. Plants. 2023;12:3064.
[29] El-Aty MSA, Abo-Youssef MI, Sorour FA, Salem M, Gomma MA, Ibrahim OM, et al. Performance and stability for grain yield and its components of some rice cultivars under various environments. Agronomy. 2024;14:2137.
[30] Mullualem D, Tsega A, Mengie T, Fentie D, Kassa Z, Fassil A, et al. Genotype-by-environment interaction and stability analysis of grain yield of bread wheat (Triticum aestivum L.) genotypes using AMMI and GGE biplot analyses. Heliyon. 2024;10.
[31] Evlice AK, Pehlivan A, Şanal T, Canay FÜ, Avcıoğlu R, Keskin Ş, et al. Assessment of durum wheat (Triticum durum Desf.) genotypes for grain yield and quality in breeding programmes under rainfed conditions. INDIAN JOURNAL OF GENETICS AND PLANT BREEDING. 2025;85:68-77.
[32] Tulu L, Wondimu A. Adaptability and yield stability of bread wheat (Triticum aestivum) varieties studied using GGE-biplot analysis in the highland environments of South-western Ethiopia. African Journal of Plant Science. 2019;13:153-62.
[33] Tekle HT, Tsehaye Y, Atsbeha G, Abera FA, Chiulele RM. Investigation of genotype x environment interaction for Hordeum vulgare L. ssp. vulgare recombinant inbred lines in multi-environments of Tigray, Ethiopia. Ecological Genetics and Genomics. 2024;31:100231.
[34] Kaloki P, Trethowan R, Tan DK. Effect of genotype× environment× management interactions on chickpea phenotypic stability. Crop and Pasture Science. 2019;70:453-62.
[35] Shukla G. Some statistical aspects of partitioning genotype-environmental components of variability. 1972.
[36] Finlay , Wilkinson. The analysis of adaptation in a plant-breeding programme. Australian journal of agricultural research. 1963;14:742-54.
[37] Carvalho RF, Silva TS, Pereira AKdS, Cavallini GS, Pereira DH. Theoretical and Experimental Study of the Stability of Thiamethoxam Under Different Environmental Conditions. Processes. 2024;12:2328.
[38] Fox J, Weisberg S. Using car and effects Functions in Other Functions. Published online. 2020;1.
[39] Dumble S, Frutos Bernal E, Galindo V. Package GGEBiplots. Version 01. 2022;3.

[40] Hilmarsson HS, Rio S, Sánchez JIy. Genotype by environment interaction analysis of agronomic spring barley traits in Iceland using AMMI, factorial regression model and linear mixed model. Agronomy. 2021;11:499.
[41] Francis T, Kannenberg L. Yield stability studies in short-season maize. I. A descriptive method for grouping genotypes. Canadian journal of plant science. 1978;58:1029-34.
[42] Purewal SS, Kaur P, Salar RK. Chickpea and Cowpea: Nutritional Profile, Processing, Health Prospects and Commercial Uses: CRC Press; 2023.
[43] Wricke G. Uber eine methode zur erfassung der okologischen streubreite in feldversucen. z Pflanzenzuchtung. 1962;47:92-6.
[44] Eberhart St, Russell W. Stability parameters for comparing varieties 1. Crop science. 1966;6:36-40.
[45] Olivoto T, Lúcio ADC. metan: An R package for multi-environment trial analysis. Methods in Ecology and Evolution. 2020;11:783-9.
[46] Delesa A, Dabi A, Alemu G, Geleta N, Solomon T, Zegeye H, et al. Stability and performance evaluation of advanced bread wheat (Triticum aestivum L.) genotypes in low to mid altitude areas of Ethiopia. International Journal of Bio-resource and Stress Management. 2023;14:19-32.
[47] Gebrie G, Abebe D. Adaptation and performance evaluation of bread wheat (Triticumaestivum L.) varieties in Guagusa-shikudad District of North western Ethiopia. Asian J Plant Sci Res. 2020;10:64-8.
[48] Birhanu A, Tahir Z, Tilahun M, Tilahun M, Hailu A. Performance Evaluation and Adaptation of Food Barley (Hordeum Vulgare L.) Varieties in the Highlands of North Gondar Ethiopia. American Journal of Agriculture and Forestry. 2020;8:252-7.
[49] Dabi A, Alemu G, Sime B, Geleta N, Delessa A, Solomon T, et al. Genotype× Environment interaction and stability analysis using GGE biplot for grain yield of bread wheat (Triticum aestivum) genotypes under low moisture stress areas of Ethiopia. International Journal of Bio-resource and Stress Management. 2023;14:1089-98.
[50] Rharrabti Y, del Moral LG, Villegas D, Royo C. Durum wheat quality in mediterranean environments: III. Stability and comparative methods in analysing G× E interaction. Field Crops Research. 2003;80:141-6.
[51] Assefa A, Girmay G, Alemayehu T, Lakew A. Performance evaluation and stability analysis of malt barley (Hordeum vulgare L.) varieties for yield and quality traits in Eastern Amhara, Ethiopia. CABI Agriculture and Bioscience. 2021;2:31.
[52] Alemu YA, Anley AM, Abebe TD. Genetic variability and association of traits in Ethiopian durum wheat (Triticum turgidium L. var. durum) landraces at Dabat Research Station, North Gondar. Cogent Food & Agriculture. 2020;6:1778604.
[53] Kassie MM, Abebe TD, Desta EA, Tadesse W. Multi-Environment Evaluation of Bread Wheat Genotypes for Yield Stability in Ethiopia Using AMMI and GGE-Biplot Analyses. Crop Breeding, Genetics and Genomics. 2025;7.
[54] Chimdesa O, Asefa K, Alemu S, Teshome G. Evaluating Agronomic Performance and Yield Stability of Improved Bread Wheat Varieties across Low Moisture Stress Areas of Guji Zone, Southern Oromia. International Journal of Research in Agriculture and Forestry. 2019;6:1-10.
[55] Yan W, Kang MS, Ma B, Woods S, Cornelius PL. GGE biplot vs. AMMI analysis of genotype-by-environment data. Crop science. 2007;47:643-53.
[56] Tadesse Z, Habtemariam Zegeye DA, Tafesse Solomon YS, Debele T. Identification of Stable Bread Wheat (Triticum Aestivum L.) Genotypes using AMMI Analysis in Ethiopia. Int J Res Agric For. 2018;5:6-14.
[57] Mohammadi R, Amri A. Analysis of genotype× environment interaction in rain-fed durum wheat of Iran using GGE-biplot and non-parametric methods. Canadian Journal of Plant Science. 2012;92:757-70.
[58] Pour-Aboughadareh A, Barati A, Koohkan SA, Jabari M, Marzoghian A, Gholipoor A, et al. Dissection of genotype-by-environment interaction and yield stability analysis in barley using AMMI model and stability statistics. Bulletin of the National Research Centre. 2022;46:19.

[59] Mohammadi M, Sharifi P, Karimizadeh R, ALT JAFARBY J, Khanzadeh H, Hosseinpour T, et al. Stability of grain yield of durum wheat genotypes by AMMI model. Agriculture & Forestry/Poljoprivreda i šumarstv. 2015;61.
[60] Yan W, Tinker NA. Biplot analysis of multi-environment trial data: Principles and applications. Canadian journal of plant science. 2006;86:623-45.
[61] Gauch Jr HG, Zobel RW. Identifying mega-environments and targeting genotypes. Crop science. 1997;37:311-26.
[62] Scavo A, Mauromicale G, Ierna A. Dissecting the genotype× environment interaction for potato tuber yield and components. Agronomy. 2022;13:101.
[63] Khare V, Shukla RS, Pandey S, Singh SK, Singh C. Exploring the genotype-environment interaction of bread wheat in ambient and high-temperature planting conditions: a rigorous investigation. Scientific reports. 2024;14:2402.
[64] Demelash H. Genotype by environment interaction, AMMI, GGE biplot, and mega environment analysis of elite Sorghum bicolor (L.) Moench genotypes in humid lowland areas of Ethiopia. Heliyon. 2024;10.
[65] Wardofa GA, Asnake D, Mohammed H. Gge biplot analysis of genotype by environment interaction and grain yield stability of bread wheat genotypes in central ethiopia. Journal of plant breeding and genetics. 2019;7:75-85.
[66] Adem Wado D. GENOTYPE X LOCATION X PLANTING PATTERN INTERACTION AND STABILITY ANALYSIS OF SORGHUM [Sorghum bicolor (L.) Moench] VARIETIES IN DRYLANDS OF ETHIOPIA: Haramaya university; 2020.
[67] Yan F, Polk DB. Probiotic bacterium prevents cytokine-induced apoptosis in intestinal epithelial cells. Journal of biological chemistry. 2002;277:50959-65.
[68] Mullualem D, Tsega A, Mengie T, Fentie D, Kassa Z, Fassil A, et al. Genotype-by-environment interaction and stability analysis of grain yield of bread wheat (Triticum aestivum L.) genotypes using AMMI and GGE biplot analyses. Heliyon. 2024;10:e32918.
[69] Vaezi B, Pour-Aboughadareh A, Mohammadi R, Mehraban A, Hossein-Pour T, Koohkan E, et al. Integrating different stability models to investigate genotype× environment interactions and identify stable and high-yielding barley genotypes. Euphytica. 2019;215:1-18.
[70] Wagaw K, Seyoum A, Tadesse T, Nega A, Gebreyohannes A, Bejiga T, et al. Distinguishing of stable genotypes and mega environment for grain yield performance of sorghum [Sorghum bicolor (L.) moench] genotypes using spatial analysis. American Journal of Plant Sciences. 2021;12:417-31.
[71] Bekana G. Genotype X Environment Interactions and Yield Stability of Tef (Eragrostis tef) Genotypes Grown in Central Parts of Ethiopia. American Journal of BioScience. 2023;11:104-10.
[72] Kaya Y, Akçura M, Taner S. GGE-biplot analysis of multi-environment yield trials in bread wheat. Turkish journal of agriculture and forestry. 2006;30:325-37.
[73] Abraha MT, Shimelis H, Solomon T, Hailu A. Genotype-by-environment interaction and selection of elite wheat genotypes under variable rainfall conditions in northern Ethiopia. Journal of Crop Improvement. 2019;33:797-813.
[74] Eskezia A, Kefale H, Asrat M. Genotype by environment interaction and yield stability analysis of bread wheat (Triticum aestivum L.) varieties in East Gojjam Zone, Northwest Ethiopia. Heliyon. 2025;11.
[75] Nadim MKA, Rahman M, Sikder MR, Adhikary S, Atiq NB, Alam MM, et al. Performance, adaptability, and multi-trait stability of rice varieties (Oryza sativa L.) in rainfed conditions. Agrosystems, Geosciences & Environment. 2025;8:e70038.
[76] Atsbeha G, Mekonnen T, Kebede M, Haileselassie T, Tesfaye K. Multivariate analyses, heritability and genotype environment interaction of bread wheat genotypes in Ethiopia. Ecological Genetics and Genomics. 2023;29:100209.

[77] Alemu G, Dabi A, Geleta N, Duga R, Solomon T, Zegaye H, et al. Genotype× environment interaction and selection of high yielding wheat genotypes for different wheat-growing areas of Ethiopia. Am J Biosci. 2021;9:63-71.
[78] Gebrewahid L, Mengistu DK, Tsehaye Y, Aberha A, Aberra DA. Variability among Ethiopian durum wheat genotypes grown under different climatic conditions of Tigray for some agronomic and grain-quality traits. Journal of Crop Improvement. 2021;35:184-203.
[79] Mansour E, Moustafa ES, El-Naggar NZ, Abdelsalam A, Igartua E. Grain yield stability of high-yielding barley genotypes under Egyptian conditions for enhancing resilience to climate change. Crop and Pasture Science. 2018;69:681-90.
[80] Delesa A, Alemu G, Geleta N, Dabi A, Zegeye H, Solomon T, et al. Stability and performance evaluation of advanced bread wheat (Triticum aestivum L.) genotypes in optimum areas of Ethiopia. International Journal of Bio-resource and Stress Management. 2022;13:69-80.

Genotype by Environment Interaction and Yield Stability Analysis of Durum Wheat (*Triticum turgidum* var. *durum*) Varieties Using AMMI and GGE biplot Analysis in Amahara Region, Northwest Ethiopia

Alemnesh Eskezia*[1,] Alamir Ayenew,[2] Yohannes Azene[3], Mulugeta Aytenew[1,] Kelemu Nakachew [1]

[1]Department of Plant Science, College of Agriculture and Natural Resources, Debre Markos University, P.O.Box 269. Ethiopia.

[2]Fogera National Rice Research and Training Center, Ethiopian Institute of Agricultural Research, Ethiopia.

[3]Gonder Agricultural Research Center, P.O.Box 1337, Gondar, Ethiopia

Corresponding author contact: alemnesh_eskezia@dmu.edu.et. Phone number: +251918314065

Abstract

Durum wheat is a staple crop for over one- third of the global population; however, its production in Ethiopia, particularly in the Amhara Region, faces major challenges due to limited adaptable varieties, poor management practices, and environmental constraints. Newly released wheat varieties often fail in unfamiliar environments, leading to yield losses. To address this issue, a study was conducted to evaluate the adaptability, performance, and yield stability of recently released high-performing durum wheat cultivars in the Amahara region. The field trials were conducted over two consecutive years (2022/2023 and 2023/2024) at three districts ; Simada, Bichena, and Dabat, using a Randomized Complete Block Design with three replications. Data were collected on main agronomic traits, including growth, phenological, and yield traits such as grain yield. SAS 9.4 and R software were used, showing significant differences in crop phenological stages, growth, and yield parameters across years and locations for tested traits. Additive Main Effect and Multiplicative Interaction(AMMI) and Genotype and Genotype × Environment (GGE) biplot analysis were used to study genotype by environmental interaction(GEI). Genotype, location, and year interactions significantly influenced all durum wheat genotypes. The combined analysis of variance for all tested traits including grain yield, showed highly significant effects ($p< 0.001$). Grain yield varied from 4.5 t ha^{-1}in Dabat to 2.29 t ha^{-1}in Simada. Among the genotypes, Tesfaye had the highest mean yield (3.74 t ha^{-1}), while Wehabit had the lowest (2.75 t ha^{-1}). AMMI analysis of result revealed significant interactions and differences between genotypes and environments, with genotype (16.6%),

environment (29.78%), and genotype x environment interaction (16.84%).The GGE biplot and AMMI analysis identified Tesfaye and Don metteo as stable genotypes, while Simada and Bichena as representative environments. The GGE biplot analysis grouped the six test environments and nine genotypes into two mega-environments and six genotype groups. This study identified that Tesfaye most productive and stable wheat variety in the study area and suggested farmers should adopt it to increase wheat yield per unit area of land.

Keywords; ASV, PCA, Stability, Wheat, YSI

INTRODUCTION

Durum wheat (*Triticum turgidum var. durum*) is an indigenous tetraploid wheat species (2n = 4x = 28) that has been cultivated in Ethiopia since ancient times [1]. The country is considered one of the centers of diversity for tetraploid wheat [2, 3]. Traditionally, this crop grown is in heavy black clay soils (vertisols) under rain-fed conditions at altitudes ranging from 1800 to 2800 meters above sea level [4]. The proportion of durum wheat in the total wheat-growing area has declined significantly due to the introduction of semi-dwarf, fertilizer-responsive bread wheat varieties, resulting in a shift where bread wheat now occupies the largest share of production [5, 6]. However, the area dedicated to durum wheat is expected to increase because of rising urban demand for pasta products and the expansion of irrigated wheat production in Ethiopia [7, 8].

Wheat is essential for Ethiopia's agricultural development and food security, as it is a significant source of protein and energy [9-11]. Although wheat is a staple for one-third of the global population, Ethiopia struggles to achieve self-sufficiency due to variable environments. Studying GEI helps identify stable, high-yielding varieties adapted to these diverse conditions [12, 13]. Wheat is crucial to the country's agricultural plans and sustainable development initiatives because of its nutritional value and versatility[14].

In Ethiopia, wheat covered approximately 1.87 million hectares, yielding approximately 5.8 million tons of grain during the 2021/22 cropping season [15]. In the Amhara region, wheat cultivation spanned 689,614.06 hectares, yielding 1.92 million tons with productivity of 2.8 t ha^{-1} [15]. Overall, wheat is a staple crop in Ethiopia and is vital for food security and economic

growth [16]. However, its productivity is low in many regions, including the Amhara Region. This is because of different yield-limiting factors such as diseases, pests, climate variability, overuse of local varieties, lack of high-yielding and stable varieties, and poor communication between farmers and researchers. Nowadays, Ethiopia's regional and national agricultural research institutes have released several durum wheat varieties [17, 18]. Conversely, many of these varieties have not undergone comprehensive evaluation across the country, particularly in the northwestern regions [19]. To fill this gap, it is essential to generate evidence on genotypic performance and adaptability under diverse environmental conditions. Such information is crucial for devising effective intervention strategies to enhance crop productivity and promote dietary self-sufficiency [20].

Durum wheat breeding programs in Ethiopia rely on elite lines from various sources, but their performance often varies across environments due to genotype-by-environment interaction (GEI)[21, 22]. To ensure stable and high-yielding varieties, multi-environment trials (METs) are essential for assessing performance and stability [23, 24]. GEI complicates selection decisions because genotypes may perform inconsistently across locations, exhibiting either non-crossover (differences in magnitude) or crossover (changes in rank) interactions [25]. Therefore, relying solely on mean yield is therefore inadequate, highlighting the need for robust GEI analysis to guide breeding and variety release [26, 27].

The AMMI model integrates ANOVA for additive effects and PCA for multiplicative interactions, enabling clear stability assessment through metrics such as the AMMI Stability Value. AMMI and GGE biplot were chosen due to strong GEI in the data. AMMI analyzes stability, while GGE helps visualize ideal genotypes and environments. Together, they offer a comprehensive view for genotype evaluation. Although these methods have been extensively applied in crops such as like maize, rice, and bread wheat, their use in durum wheat multi-environment trials remains limited in Ethiopia[28, 29]. Previous studies in Ethiopia and elsewhere have demonstrated the effectiveness of AMMI and GGE in identifying stable and high-yielding durum wheat varieties, such as the works of [18, 29, 30] and [31], which reported the identification of genotypes with broad adaptability and mega-environment delineation. Our study builds on these findings by applying both methods to durum wheat in the Amhara region under rainfed conditions, generating practical insights for stability, adaptability, and variety

recommendation across diverse agro-ecologies. This dual-method approach supports robust decision-making in durum wheat breeding and variety recommendation [32, 33].

Given the significant influence of environmental variability on genotype performance, stability assessment is crucial in plant breeding to identify genotypes with consistent yield across environments [34]. Stability measures, such as Shukla's stability variance, which quantifies each genotype's contribution to GEI with lower values indicating greater stability [35], and the regression-based method developed by [36],which assesses genotype sensitivity and adaptability, have been foundational in this field. Genotypic stability is commonly categorized into two forms: static stability, where genotype performance remains relatively constant across environments, and dynamic stability, where performance changes in a consistent and predictable manner in response to environmental variation [37]. These complementary concepts are critical for identifying genotypes that combine high productivity with reliable performance across diverse environmental conditions. Therefore, the current study was conducted to evaluate the adaptability, performance, and yield stability of recently released high-performing durum wheat cultivars in the Amahara Region, northwestern Ethiopia.

2. MATERIALS AND METHODS

2.1. Descriptions of the Study area

The study was conducted across three wheat-producing districts: Simada, Bichena, and Dabat, situated within the Aamara region, Ethiopia, with permission from the Amhara Regional Agricultural Research Institute (ARARI) and Debre Markos university (DMU) (Table 1). Specifically, it was conducted at the Farmers Training Centers (FTC) in each district during the main crop or rainy seasons between June and December 2022-2024 for two consecutive years. Soils in the tested location are Vertisols, with a pH of 5.5–7.0, moderate organic matter, and clayey texture. They contain moderate organic matter but face challenges such as phosphorus deficiency, erosion, and waterlogging in lowland areas. The locations were chosen purposively based on wheat production potential and soil type.

Table 1.Geographical description of the study area

Location	Code Year 2021/2022	Code Year 2022/2023	Alt. (masl)	Temp. min & max values (°C) Each year		RF. Ave.(mm)		Soil type	Geographic location	
				2022	2023	2022	2023		Lat	Lon
Simada	E1	E4	[illegible]00-2220	19-27	19-25	1012	1320	Vertisol	10 °22' N	37° 47' E
Bichena	E2	E5	2250	15.19	13-21	882	1256	Vertisol	10 38' N	10° 37' E
Dabat	E3	E6	2446	17-23	12-19	1320	1470	Vertisol	10°21` N	37° 43`E

Where: Alt: Altitude, Temp: temperature, and RF: Rainfall, Lat: latitude, Lon: longitude

Source:Districta dministrative office

2.2. Experimental material and design

Seeds of eight improved, high-yielding durum wheat varieties were obtained from the Kulumsa Agricultural Research Center, and one variety was collected from the Adet Agricultural Research Center in Amhara, Ethiopia (Table 2). All varieties were evaluated under rainfed conditions during the 2022 and 2023 main cropping seasons and were sown on well-prepared experimental plots. These nine varieties were tested across three environments (locations). Each experimental unit (plot) consisted of nine rows with 20 cm spacing between rows, resulting in a plot area of 3.6 m^2 (1.8 m × 2 m). The field was plowed three times before sowing, and planting rows were prepared using a hand-pulled row marker. Seeds were manually planted using the hand drilling technique. A spacing of 0.5 m was maintained between plots, and 1 m was left between blocks. Since the two years (2022 and 2023) were also considered as blocks, the six combinations of the field blocks and years were used as blocks in the multilocation trial model. For each plot, a standard fertilizer application of 100 kg/ha NPS and 150 kg/ha urea was applied, following recommendations for durum wheat. Half of the urea and the full amount of NPS were applied during planting, while the remaining half of the urea was applied at the mid-tillering stage. A seed rate of 150 kg/ha was used. Weeding was carried out manually across all experimental fields at the three locations to manage weeds. No herbicides or fungicides were applied for weed or disease control.

Table 2. Variety used in the study

S. N	Variety code	Variety name	Pedigree	Year of release	Center	Maturity period	Disease resistance levels	Yied on station	Area of daptation
1	G1	Fetan	CDSS02B00643 S-0Y-0M-1Y4M-04-Y-0B-2Y	2018	DZARC	95-110	Moderately resistance	46-50	500-2600
2	G2	Don Matteo	43IDSNMeh 2011-82	2018	CGS Italian	115-130	Moderately resistance	50-55	1500-2750
3	G3	Alemtena	43IDSNMeh 2011-130	2015	DZARC	95-110	Moderately resistance	44-50	500-2600
4	G4	Tesfaye	ARMENT//SRN-NIGRIS-4/3/CANED-9.1/4/TOSKA-26RASCON-37//SNITSN /5/PLAYERO	2017	DZARC	110-130	Moderately resistance	50-55	1600-2800
5	G5	Wehabit	Waha (plc/Ruff//Gta/Rtte)	2017	Mekelle University	NA	Moderately resistance	NA	NA
6	G6	Rigeat	Acc#208304	2017	Mekelle University	NA	Moderately resistance	NA	NA
7	G7	Utuba	(= Icajihan42) Omruf1/Stojocri2/3/1718/BeadWheat 24//Karimï¿½	2015	DZARC	105-118	Moderately resistance	53-57	1880-2700
8	G8	Mangudo	Mangudo/Mekuye/DZ 2013 meh DW F1 P#20/DZ 2014 meh DW F2 P#19-8	2012	SARC/ORARI	110-124	Moderately resistance	45-50	1880-2700
9	G9	Toltu	4/B/R9096#21001(980SN Patho)	2010	SARC/ORARI	NA	Moderately resistance	NA	NA

2.3. Data Collected

The data for the eleven traits were collected on a plant-and-plot basis. Data were obtained on a plot basis based on all plants in the central rows of each plot, leaving two border row from each side of the plots. The ten representative plants per plot were randomly selected from the central rows for recording data on plant basis. Agronomic traits such as crop phenology, growth, yield, and yield components were assessed during the study period. Crop phenology included maturity day and days to heading, whereas growth parameters, such as plant height, number of productive tillers, number of grains per spike, number of spikelets per spike, spike length, and yield-related traits, including biomass yield, thousand seed weight, number of seed per spikelet, grain yield, and harvest index, were recorded.

Days to heading (days): The number of days from sowing to when the tips of the spike first emerged from the main shoot in 50% of the plant population within a plot was recorded.

Days to 90% maturity (days): The number of days from sowing to physiological maturity was recorded. Physiological maturity was determined when 90% of the crop stands in a plot showed a light yellow (straw) color in their stems, leaves, and floral bracts.

Number of productive tillers (no): The number of productive tillers was determined at maturity by counting all spike-producing tillers of 10 plants per plot.

Number of spikelets per spike (no): The number of spikelets per spike was counted from ten representative spikes per plant, and the average was calculated.

Plant height (cm) was measured from the base of the main stem to the tip of the spike from an average of ten randomly selected plants in the central four rows in each plot.

Spike length (cm): This was recorded at the physiological maturity stage by measuring the middle rows of 10 randomly tagged plants from the base of the spike to the tip of the spike (excluding the awns), and the average length of the plant was calculated.

Biomass yield (ton ha^{-1}): Aboveground total biomass was recorded after sun-drying, attained constant weight, and then converted to t ha^{-1}.

Grain yield (ton ha^{-1}): The weight of air-dried seeds harvested from each plot was recorded. Then, the yield from the net plot area was converted into t ha^{-1}

Harvest index (%): The ratio of grain yield per plot to biological yield per plot is expressed as a percentage.

$$HI (\%) = \frac{\text{Grain Yield}}{\text{Biomass Yield}} \times 100$$

2.4. Data Analysis

Data were analyzed using R statistical software version 4.4.1 (R Core Team, 2021). Analysis of variance (ANOVA) was conducted on the multi-environment data using the aov function from the car package [38] to evaluate the significance of genotype-by-environment interactions. The GGE model function from the GGE Biplots package was applied to the genotype-by-environment data matrix [39]. Subsequently, the "Which-Won" function was used to generate the "which-won-where" view of the GGE biplot. The Env relationship function in the same package was utilized to plot environmental relationships, the Disc Rep function was used to assess discrimination ability and representativeness, and the Mean Stability function was applied to create the discrimination ability and representativeness view and stability plots.

AMMI analysis was carried out using the AMMI function in the agricolae package [40].Genotype stability was further assessed using additional stability parameters, including the coefficient of variation (CV) [41] Shukla's stability variance [35], AMMI Stability Value (ASV) [42], ecovalence (Ecoval) [43], and deviation from joint regression analysis (Sij)[44].These parameters were estimated using the metan package in R [45].

The ASV measures the stability of genotypes based on their interactions with the environment. Lower ASV values indicate more stable genotypes.

AMMI stability value was calculated as follows:

$$ASV = \sqrt{[(\text{SSIPCA1} / \text{SSIPCA2})\ (\text{IPCA1 score})]2 + (\text{IPCA2 score})2}$$

Where; SSIPCA1 and SSIPCA2 are the sums of the squares of the first and second principal component axes (IPCA), respectively. The IPCA 1 and IPCA2 scores are the interaction scores for the first and second IPCAs, respectively.

GGE biplot analysis is a graphical method that evaluates genotype performance and stability across environments by combining genotype and genotype-environment interaction effects, helping to identify the best and most adaptable genotypes.

$$Yij - \mu - \beta j = \sum_{k=1}^{n} \lambda k \xi ik \eta jk + \varepsilon ij$$

Where:

- Yij is the grain yield of genotype i in environment j,
- μ is the grand mean,
- βjis the main effect of environmentj,
- λk is the singular value for principal component k,
- ξik andηjk are the genotype and environment scores for PCk, respectively,
- εij is the residual.

3. Results and Discussion

3.1. Combined analysis of Variance (ANOVA)

The analysis of variance revealed highly significant differences ($P < 0.01$) among varieties for the traits considered in the experiment (Table 3). Locations also exhibited highly significant difference ($p<0.01$) for days to heading, tiller number, spike length, number of seeds per spikelets, plant height, thousand seed weight, biomass yield, grain yield, and harvest index. Additionally, varieties demonstrated highly significant variation ($P < 0.01$) among all evaluated traits. The year showed a highly significant effect ($p<0.01$) on plant height, spike length, biomass yield, grain yield, and thousand seed weight.

The interaction between variety and location (V x L) was highly significant ($P < 0.01$) for all traits tested in the durum wheat varieties. Similarly, the interaction between variety and year (V x Y) was highly significant ($p < 0.01$) for plant height, number of grains per spike, grain yield, and harvest index. The interaction between location and year (L x Y) was also highly significant ($p < 0.01$) for all tested traits except for the number of seeds per spikelet and biomass yield.

The three-way interaction effect of variety, location, and year (V x L x Y) was highly significant ($p < 0.01$) for days to heading, plant height, tiller number, spike length, number of seeds per spikelets, thousand seed weight, biomass yield, grain yield, and harvest index. The main effect of

location by year (LxY) had highly significant ($p < 0.01$) effect on days to heading, maturity day, plant height, and grain yield. However, non significant effect was observed for the number of seeds per spikelet and biomass yield. The interaction effect of location by year had a highly significant ($P < 0.01$) effect on days to heading, maturity day, plant height, and grain yield, however, non significant effect was observed for the number of seeds per spikelet and biomass yield. As shown in Table 3, the interaction effect of location and variety had a significant ($P < 0.05$) effect on all evaluated traits. The interaction effect of variety by year was highly significant ($P < 0.01$) for plant height, number of seeds per spikelet, biomass yield, grain yield, and spike length. Conversely, a non-significant effect was observed for maturity date and days to heading. Overall, the combined analysis revealed significant variations among the nine durum wheat varieties for most traits. These findings are consistent with previous studies by [46], which reported significant differences ($p < 0.05$) among durum wheat genotypes across all individual test locations over multiple years.

Days to heading, plant height, tiller number, biomass yield, grain yield, and harvest index were all highly significant ($p \leq 0.01$), as shown in Table 3. However, maturity day and the number of seeds per spike were not influenced by the interaction effects of year, variety, and location. In line with the present results, previous studies have reported highly significant differences ($p \leq 0.01$) [47]. [48] also identified significant genotype-by- location interactions, with highly significant differences ($p \leq 0.01$) among food barely varieties for days to maturity and grain yield. The observed significant GEI interaction indicate inconsistent performance across the tested environments and highlight the varying discriminative abilities of these environments. This suggests the possibility to identify high-yielding and stable genotypes. A particular crop variety may perform well in one environment but poorly in another due to variations in climate, soil conditions, or management practices.

The results of this study are consistent with previous research by [49], which reported significant difference ($P < 0.05$) among bread wheat genotypes for grain yield at all individual test locations in multi-environment trials.

Table 3. Mean squares of the two years (cropping season) combined ANOVA results of Eleven quantitative traits from three locations

**SOV**	Mean squares									
	**DH**	**DM**	**PH**	**TN**	**SL**	**NSPS**	**TSW**	**BY**	**GY**	**HI**
Genotypes (G)	345.28**	789.84**	585.36**	1.36**	11.475**	34.85**	226.28**	5.99**	2.07**	86.20**
Location (L)	6.968^{ns}	194.93^{ns}	41.61**	47.51**	43.41**	24.83**	234.07**	25.21**	4.319**	165.47**
Year (Y)	0.964^{ns}	89.92^{ns}	164.36**	0.03^{ns}	0.30^{ns}	2.45^{ns}	27.16**	3.43**	4.200**	1.05ns
V x L	42.69*	187.61*	222.18**	2.02**	11.54**	23.51**	120.06**	2.49**	0.511**	54.12**
V x Y	39.36^{ns}	95.88^{ns}	37.53**	0.59**	1.29**	7.30**	22.20**	0.42ns	0.98**	13.19ns
L x Y	289.05**	496.186*	173.19**	1.49**	2.57**	3.25^{ns}	95.82**	0.750ns	2.003**	59.46**
L x rep	33.73^{ns}	188.16^{ns}	6.47^{ns}	0.22^{ns}	1.36**	5.10^{ns}	12.34**	0.63ns	0.037ns	27.26ns
V x L x Y	53.39**	152.547^{ns}	33.32**	0.33**	1.35**	3.43^{ns}	18.86**	1.015**	0.859**	53.28**
Residuals	22.59	103.58	10.45	0.12	0.33	2.74	2.53	0.41	0.02	14.35

Where SOV: source of variation; **: highly significant at $P < 0.01$; *: significant at $P < 0.05$; ns: non-significant; DF: Degrees of freedom; DH: days to heading; DM: days to maturity; PH: plant height; TN: tiller number; SL: Spike length; NSPS: number of spikelets per spike; BY: biomass yield; NSS: number of seed per spike, TSW: thawed seed weight, GY: grain yield; and HI: harvest index.

3.2. Performance of the Genotype

The grain yield performance of the durum wheat varieties showed significant variability across the tested locations. Tesfaye (G4) recorded the highest grain yield 3.74 t ha^{-1}, indicating its superior adaptability and productivity under the test conditions. It was followed by Don Matteo (G2) which achieved a yield of 3.62 t ha^{-1}, also demonstating strong performance. In contrast, Wehabit (G5) and Rigeat (G6) exhibited the lowest grain yields, at 2.75 t ha^{-1} and 2.78 t ha^{-1}, respectively, suggesting their limited potential for high productivity in these environments.

The differences in grain yield among the genotypes can be attributed to variations in key yield components such as tiller number, spike length, and thousand seed weight. These findings underscore the importance of selecting high-yielding genotypes, such as Tesfaye (G4) to enhance durum wheat production in the study area. Our results are consistent with those of [50] and [51], who found that durum wheat genotypes varied significantly in terms of adaptation and grain yield across Ethiopian highlands. Tesfaye and Don Matteo, in particular were found to be promising genotypes that consistently performed well in a variety of environmental settings. Similar to the current study, [52] observed that cultivars like Wehabit showed reduced grain yield stability. While Wehabit and Rigeat's subpar performance highlights the necessity for location-specific recommendations, Tesfaye's improved performance across locales demonstrates its appropriateness for wider adaption.

The observed variability in grain yield reflects the influenced GEI interaction. High-yielding genotype, such as Tesfaye (G4) 3.74 t ha^{-1} demonstrate better adaptability and efficient resource utilization , whereas low-yielding genotype like Wehabit (G5) 2.75 t ha^{-1} show poor adaptability to the tested environments (Table). This result further supports the importance of evaluating durum wheat genotypes across multiple environments to identify stable and high-yielding varieties. Selecting adaptable varieties like Tesfaye (G4) will be vital to enhance durum wheat production and support food security goals in Ethiopia.

Table 4. Mean grain yield and yield-related traits of durum wheat genotypes tested at three locations for two years

Genotypes	DH	DM	PH	TN	SL	NSPS	TSW	BY	GY	HI
1	62.00^{b}	104.44^{bcd}	87.17^{cd}	3.81^{cd}	7.62^{d}	19.43^{a}	35.47^{c}	10.47^{b}	3.3^{c}	26.74^{e}
2	61.22^{b}	110.72^{ab}	98.01^{a}	4.01^{abc}	9.83^{a}	19.39^{a}	36.99^{b}	11.08^{a}	3.62^{b}	30.81^{abc}
3	53.44^{c}	93.33^{f}	88.29^{c}	3.45^{e}	7.69^{d}	16.36^{c}	34.46^{cd}	9.64^{c}	3.09^{d}	31.25^{ab}
4	70.72^{a}	113.39^{a}	94.23^{b}	4.18^{a}	9.06^{b}	19.36^{a}	41.23^{a}	10.94^{a}	3.74^{a}	33.17^{a}
5	62.11^{b}	106.13^{bc}	83.55^{e}	4.11^{ab}	8.47^{c}	19.16^{a}	31.44^{f}	9.40^{c}	2.75^{f}	28.37^{cde}
6	61.44^{b}	101.78^{cde}	78.53^{f}	3.9^{bc}	8.1^{c}	16.64^{bc}	31.12^{f}	9.64^{c}	2.78^{f}	27.77^{de}
7	63.39^{b}	95.23^{ef}	87.02^{cd}	3.55^{e}	7.47^{d}	16.42^{c}	29.5^{g}	10.08^{b}	2.95^{e}	28.76^{bcde}
8	62.89^{b}	102.61^{cd}	85.80^{d}	4.19^{a}	8.29^{c}	17.66^{b}	33.68^{de}	10.15^{b}	3.12^{d}	29.65^{bcd}
9	63.06^{b}	98.97^{def}	85.36^{de}	3.66^{de}	7.5^{d}	16.89^{bc}	32.63^{e}	10.18^{b}	3.15^{d}	26.63^{e}
MSE	22.59	103.58	10.45	0.12	0.33	2.74	2.53	0.41	0.02	14.35
Mean	62.25	102.96	87.55	3.87	8.22	17.92	34.06	10.18	3.17	29.24
CV	7.64	9.88	3.69	9.04	7.03	9.23	4.67	6.27	4.88	12.95
LSD	1.81	3.88	1.23	0.13	0.22	0.63	0.61	0.24	0.06	1.45

Where: MSE= Mean sequar of error LSD= List Significant Deference and CV = Cofficent of varation.

3.3. Genotype performance at each tested location.

The mean grain yield of the nine durum wheat genotypes across six environments (E1–E6) revealed notable GEI interaction, highlighting the differential performance of the genotypes under varying conditions (Table 5). Tesfaye (G4) consistently exhibited superior performance, achieving the highest mean grain yield (3.74 t ha^{-1}), driven by its exceptional adaptability in environments E1 (3.97 t ha^{-1}), E4 (3.97 t ha^{-1}), and E6 (4.5 t ha^{-1}). This indicates that Tesfaye is particularly well-suited to a range of environmental conditions. Don Matteo (G2) ranked second in overall performance with a grain yield of (3.62 t ha^{-1}), excelling in E2 (4.7 t ha^{-1}) and E4 (4.13 t $ha^{-1)}$, suggesting its suitability for high-yielding environments, especially in Simada and Bichena. In contrast, Wehabit (G5) and Rigeat (G6) recorded the lowest mean grain yield, at 2.75 t ha^{-1} and 2.78 t $ha^{-1,}$ respectively. Their consistently poor performance across most environments suggests limited adaptability or a higher sensitivity to environmental variations, making them less reliable for achieving high yields. Similarly, Alemtena (G3)**,** despite excelling in E3 (4.82 t ha^{-1}), had a lower overall mean yield (3.08 t ha^{-1}) due to inconsistent performance in other environments, indicating that its high yield potential is not consistent across all conditions. Previous studies in Ethiopia support these findings. For instance, Tesfaye and Don Matteo were identified as stable and high-yielding durum wheat varieties across several northern Ethiopian locations[22, 53]. Similarly, [54]reported that Tesfaye demonstrated broad adaptability and consistent yield performance in both highland and moisture-stressed areas. Conversely, [54] found that Wehabit exhibited poor stability across diverse environments, aligning with our results on its limited performance.

Environmental effects were evident, with Dabat 2023 (E6) producing the highest mean grain yield (3.767 t ha^{-1}), followed by Bichena 2022 (E1) and Bichena 2023 (E4), which recorded mean grain yielda of 3.256 t ha $^{-1}$and 3.191 t ha^{-1}, respectively. Conversely, Simada 2022 (E2) had the lowest mean yield (2.695 t ha^{-1}), likely due to less favorable growing conditions in that region. These results highlight the importance for evaluating genotypes across diverse environments to identify high-yielding and stable performers. Genotypes such as Tesfaye (G4) and Don Matteo (G2) should be prioritized for large-scale production in the Amhara region and similar agro-ecologies, given their demonstrated adaptability and productivity. These findings

contribute to the growing body of evidence supporting the use of multi-environment trials and stability analysis to guide durum wheat variety recommendations in Ethiopia.

Table 5. Mean grain yield (t ha^{-1}) of nine durum wheat genotypes across six environments (location and year combinations)

Genotypes	(E1)	(E4)	(E3)	(E6)	(E2)	(E5)	Mean
**1**	3.53b	3.66b	2.57d	3.67b	3.13a	3.2b	3.29
**2**	3.84a	4.13a	2.6d	4.7a	3.17a	3.26ab	3.62
3	2.74c	2.91c	4.82a	2.82c	2.41cd	2.8c	3.08
**4**	3.97a	3.97a	3.23b	4.5a	3.23a	3.53a	3.74
5	2.87c	2.79cd	2.75cd	2.83c	2.5bc	2.77c	2.75
6	2.7c	2.63d	2.92c	3.63b	2.29d	2.53c	2.78
7	2.85c	2.85cd	3.18b	3.41b	2.61b	2.8c	2.95
8	3.97a	2.91c	2.92c	3.63b	2.5bc	2.77c	3.12
9	2.85c	2.85cd	2.57d	4.7a	2.41cd	3.53a	3.15
MSE	0.03	0.023	0.015	0.031	0.007	0.03	
Mean	3.256	3.191	3.062	3.767	2.695	3.02	3.16
CV	5.314	4.773	4.035	4.642	3.125	5.62	
LSD	0.299	0.264	0.214	0.303	0.146	0.29	

Where: E1=Bichena 2022, E2=Simada 2022,E3=Dabat 2022=E4=Bichena 2023=E5=Simada 2023, E6=Dabat 2023, MSE= Mean sequar of error and CV = Cofficent of varation.

3.4. Additive Main Effects and Multiplicative Interaction (AMMI) Analysis

Additive Main effect and Multiplicative Interaction (AMMI) analysis of variance for grain yield of the nine durum wheat genotypes evaluated across six environments is presented in (Table 6). The AMMI analysis revealed genetic variation and potential for selecting stable genotypes. The results demonstrated that the variation among E, G, and GEI was highly significant ($P < 0.01$). Sum squares (SS) partitioning revealed that the environmental effect was the largest contributor to variation followed by the GEI interaction and genotype main effect. Since changes in G and G × E are ordinarily smaller, the environment often explains most of the differences in genotype performance [55].

Furthermore, the AMMI model's first two principal component axes (IPCAs) were found to be highly significant ($P < 0.01$) when used for GEI partitioning. The AMMI, which includes IPCA1 and IPCA2, is the best prediction model for cross-validating yield variation explained by the

genotype-environment interaction [56, 57]. Variations in the grain yield components from the AMMI indicated that genotype, environment, and genotype by environment interaction (GEI) all had significant ($P < 0.01$) effects, explaining variability in both environments and genotypes, and that there are possibilities for the selection of genotypes that are high-yielding, stable, and well-performing. G8 (Mangudo) was the most stable but only modestly productive, according to the AMMI1 biplot, while G4 (Tesfaye) and G2 (Don Matteo) combined high yield with good stability. Although they demonstrated strong interactions with particular surroundings, G3 (Alemtena) and G6 (Rigeat) were less stable. In contrast to E3 (Dabat 2022), which was highly interactive and showed significant environmental influence, E6 (Dabat 2023) was both stable and high yielding.

Grain yield was significantly impacted by environments, genotypes, and their interactions, according to AMMI analysis (P < 0.001). The sum of squares resulting from the GEI is further separated into five important Interaction Principal Components (PC-1 to PC-5) in consideration of the multiplicative component of AMMI (Table 6). The highest proportion of the total sum of squares was accounted for by the environment (45.96%), followed by the genotype by environment interaction (25.86%) and then genotype (25.2) (Table 6). According to Gauch (2006), this is consistent with the outcome anticipated from GEI analysis. The environment-related sum of squares' maximum magnitude indicates that the test conditions vary and have an impact on how the grain yield characteristic manifests itself. In line with findings from barley [58] and durum wheat grain yield [59] the GEI and the largest percentage of environmental variance are in accord. The higher sum of squares due to the genotype-by-environment interaction compared to the genotype effect indicates the presence of mega-environments (**Fig. 1**). Based on IPCA1 and IPCA2, Tesfaye, Fetan, and Don Matteo showed small interaction values with high yields, indicating wide adaptability and stability, while Alemtena had large IPCA scores, reflecting poor stability. Mangudo and Rigeat showed moderate adaptability. The findings reveal that the various genotypes reacted differently to the environmental conditions, highlighting the phenotypic diversity among the evaluated genotypes. This also implies the potential presence of distinct mega-environments, each with genotypes that exhibit varying performance levels.

Table 6. AMMI analysis of variance for grain yield of nine durum wheat genotypes

Source	DF	Sum square	Mean square	Proportion	Accumulated
ENV	5	29.78	3.36**	45.22%	NA
REP(ENV)	12	0.49	0.04**	0.74%	NA
GEN	9	16.6	2.08**	25.2%	NA
GEN: ENV	40	16.84	0.75**	25.86%	NA
PC1	12	20.72	1.72**	69.6	69.6
PC2	10	5.62	0.56**	18.9	88.5
PC2	8	2.11	0.26**	7.1	95.6
PC4	6	1.11	0.19**	3.7	99.3
PC5	4	0.22	0.06**	0.7	100
Residuals	96	2.15	0.02	NA	NA
Total	201	65.86	0.47	NA	NA

Genotype rank based on AMMI stability value

Number	Genotype	Mean yield	Rank	ASTAB	ASTAB	Shukula	bij	ASI	IPCA 1	IPCA 2	ASV	Rank
1	Fetan	3.295	3	3.89	5	1.23	8.36	2.76	-0.415	0.2910	1.38	7
2	Don Matteo	3.51	2	5.78	6	2.22	1.41	4.00	-0.609	0.2569	1.99	8
3	Alemtena	3.01	6	1.43	9	7.42	-1.65	7.56	0.161	0.1107	3.77	9
4	Tesfaye	3.69	1	1.41	1	2.49	1.20	1.74	-0.264	0.1547	8.73	5

5	Wehabit	2.75	9	2.93	3	3.37	3.46	1.07	0.1521	0.2138	5.38	2
6	Rigeat	2.78	8	3.64	4	5.04	1.31	1.11	0.1366	0.3365	5.57	3
7	Utuba	2.94	7	1.76	2	2.60	7.32	1.74	0.2651	-0.1175	8.69	4
8	Mangudo	3.11	4	7.53	7	1.50	1.42	9.67	0.1166	0.2995	4.83	1
9	Toltu	3.07	5	9.41	8	2.74	1.73	2.66	-0.3087	-0.8727	1.32	6

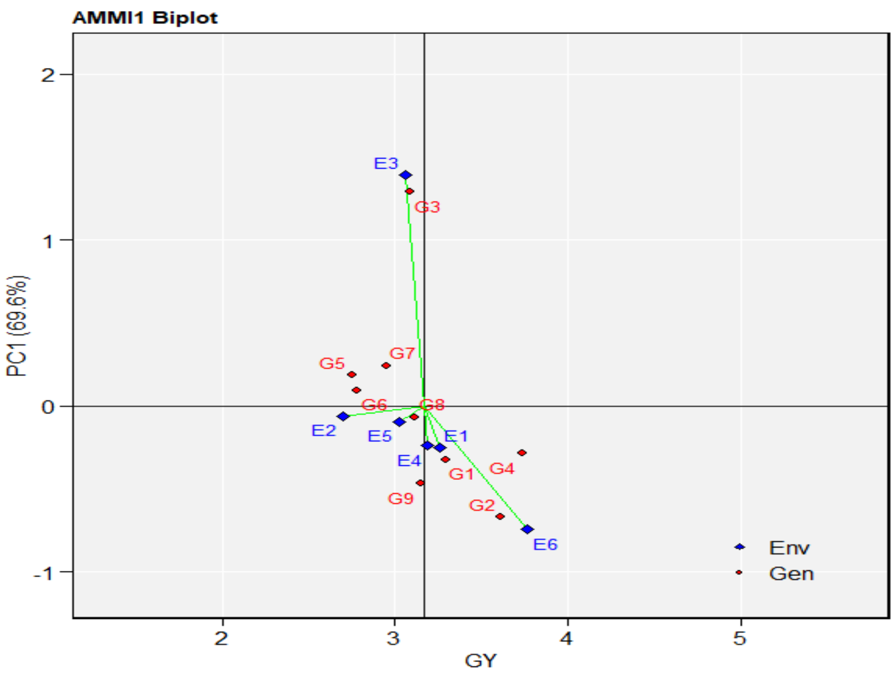

Figure 1. AMMIAnalysis model biplot for grain yield of durum wheat genotypes evaluated at six environments. Where: G1: Fetan, G2: Don Matteo, G3: Alemtena, G4: Tesfaye, G5: Wehabit, G6: Rigeat, G7: Utuba, G8:Mangudo, and G9: Toltu genotype (E1:

Simada, E2: Bichena, E3: Dabat 2022 cropping season and E4: Simada, E5: Bichena, E6: Dabat in 2023 cropping season. The red numbers connected to the polygon represent the genotypes, whereas the blue numbers represent the locations/test environments.

3.5. Stability Analysis and Mega-Environment Classification Using GGE Biplot.

3.5.1. GEI analysis for which-won-where patterns using GGE biplot

The GGE results were similar to those obtained from AMMI and combined ANOVA. The polygon view of the GGE biplot highlights the top-performing genotype in each environment, visually representing key concepts such as crossover interaction, mega-environment differentiation, and specific adaptation [60]. Mega environment analysis refers to dividing a crop-growing region into distinct target zones [61].

In this analysis, six vertex genotypes were identified: G3 (Alemtena), G4 (Tesfaye), G2 (Don Matteo), G5 (Wehabit), G6 (Rigeat), and G9 (Toltu). The biplot was divided into six sections by using six rays, with environments distributed across three of these sections while genotypes appeared in all. Each sector's vertex genotype exhibited the highest grain yield for the environments within that sector, indicating specific adaptation to particular mega-environments.

The GGE biplot analysis identified two distinct wheat-growing mega-environments.The first mega-environment comprised environments E1 (Bichena 2022), E2 (Simada 2022), E4 (Bichena 2023), E5 (Simada 2023), and E6 (Dabat 2023), with G2 identified as the winning genotype. The second mega-environment consisted solely of E3 (Dabat 2022), where G3 was the top-performing genotype. No mega-environments were associated with sectors where genotypes G5, G6, and G9 were vertex genotypes, indicating that these genotypes were not the best performers in any tested environment. G8, located near the origin, exhibited average performance with wide adaptability but moderat grain yield. The GGE biplot explained 82.67% of the total G×E variation (Axis 1: 61.48%, Axis 2: 21.19%). Previous studies by [62] have reported similar findings, suggesting that an ideal genotype should have high mean performance and stability. The genotypes that performed the best or worst in specific environments were the furthest from the biplot's origin (Bekana, 2023), which were more sensitive to environmental changes, and regarded as specifically adapted genotypes. The GGE biplot polygon view helps identify the most ideal test environments within each sector, which are crucial for selecting superior genotypes [63]. Therefore, G4 (Tesfaye) had the highest yield in Bichena and Simada 2022, G9

(Toltu) had the highest yield in Simada and Dabat, and G3 (Alemtena) had the highest yield in Dabat 2022. The most effective method for identifying superior genotypes and visualizing the patterns of interaction between genotypes and environments in data analysis is the polygon view of the GGE biplot, which helps to estimate the potential existence of various mega-environments [64, 65]. Vertex genotype G4 falls in the first and second quadrants, G9 falls in the third quadrant whereas G3 is located in the second and fourth quadrants. Therefore, the six testing environments were categorized into three mega environments, while the nine genotypes were divided into six genotypic groups (**Fig. 2**).

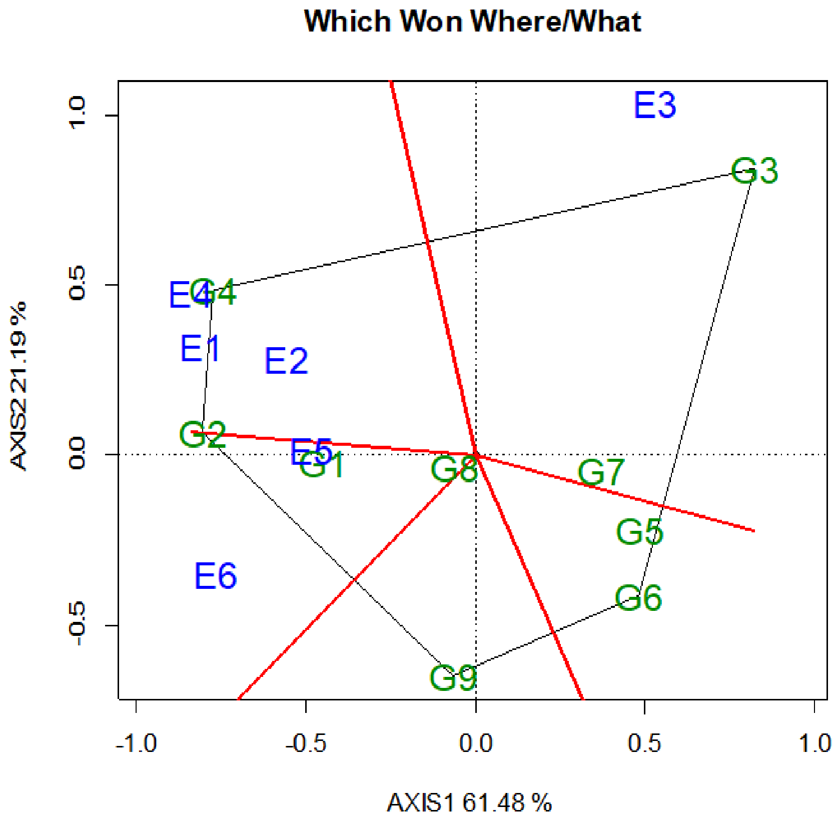

Figure 2 Which-won-where view of GGE biplot for grain yield of durum wheat genotype Where: G1: Fetan, G2: Don Matteo, G3: Alemtena, G4: Tesfaye, G5: Wehabit, G6: Rigeat, G7: Utuba, G8:Mangudo, and G9: Toltu genotype (E1: Simada, E2: Bichena, E3: Dabat 2022 cropping season and E4: Simada, E5: Bichena, E6: Dabat in 2023 cropping season. The blue numbers connected to the polygon represent the genotypes, whereas the green numbers represent the locations/test environments.

3.5.2. Evaluation of environments relative to the ideal environment

Through the representation of the center of the concentric circles in a polyg on perspective, the discrimination and representativeness of the GGE biplot often illustrate the ranking of an ideal test environment for a particular genotype or for all genotypes (**Fig. 3**). A point located on the average environment axis in the positive direction,with the longest vector from the biplot origin is considered the most representative and thus the optimal test environment in the GGE biplot [60]. The test environment that is both most representational of the target environment and most discriminating (informative) is deemed optimal. The ideal test environment is represented by the center of the concentric circles (**Fig**. 3). E5 and E2 performed the worst in selecting genotypes adapted to the entire region, whereas E1, E4, and E6 were closest to the ideal environment point and, therefore, best represented the Durum Wheat production environments.

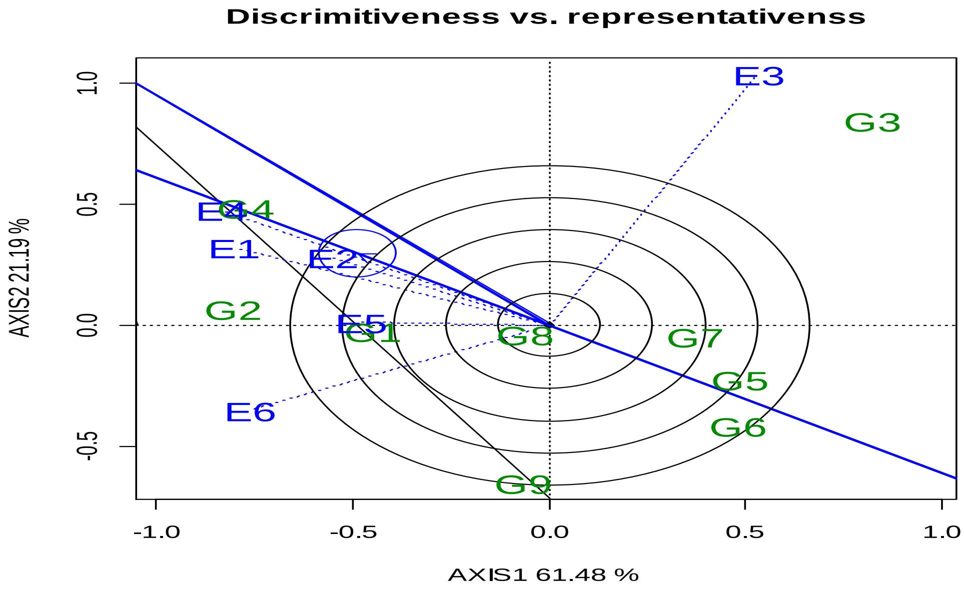

Figure 3. Discrimination vs. representativeness of genotypes. Where: E1: Simada, E2: Bichena, E3: Dabat 2022 cropping season and E4: Simada, E5: Bichena, E6: Dabat in 2023 cropping season.

3.5.3. Evaluation of genotypes relative to the ideal genotype

The ideal genotype for durum wheat production is positioned at the center of the concentric circles, in the GGE biplot symbolizing optimal traits characterized by high mean yield and stability. In this context, Tesfaye, stands out desirable genotype due to its exceptional yield performance and stable characteristics, making it highly suitable for grain production. Genotypes

that are close to the ideal genotype, such as Fetan (G1), Utuba (G7), and Wehabit (G5), are also recognized for their favorable performance and stability, aligning closely with the traits of the optimal genotype. These genotypes are therefore considered strong candidates for cultivation.

In contrast, genotypes positioned further from the ideal center specifically Don Matteo (G2), Toltu (G9), and Alemtena (G3) are considered less desirable due to their low yield performance **(fig.4)**. G8 (Mangudo) was the most stable but only modestly productive, according to the grain yield men values and stability figure. This classification is supported by data from the mean separation test referenced in fig 4, which corroborates these observations regarding yield stability and performance. Overall, the analysis highlights the value of selecting genotypes based on their proximity to the ideal genotype as depicted in the concentric circle model, of GGE biplot. This approach effectively illustrates the relationship between yield performance and genetic stability in durum wheat breeding programs. Supporting this finding, Mulugeta, Tesfaye [22] identified variety G-169 as the most ideal genotype for durum wheat grain production. G4 as the ideal genotype and E4 as the ideal environment. G1 and G2 also appear as desirable genotypes, while E1 and E2 emerge as desirable environments, suggesting they offer favorable conditions for good performance and stability (fig. 4). Additionally, Multivariate analyses of Ethiopian durum wheat revealed genotypes that are both high-yielding and stable across trials. The relative contributions of stability and grain yield for identifying desirable genotypes found in this study, using the ideal genotype procedure of the GGE biplot, were also similar to those reported by Adem Wado [66] genotype × location × planting pattern interaction and stability analysis of sorghum (Sorghum bicolor (L.) Moench) varieties in the drylands of Ethiopia.

Ranking Genotypes

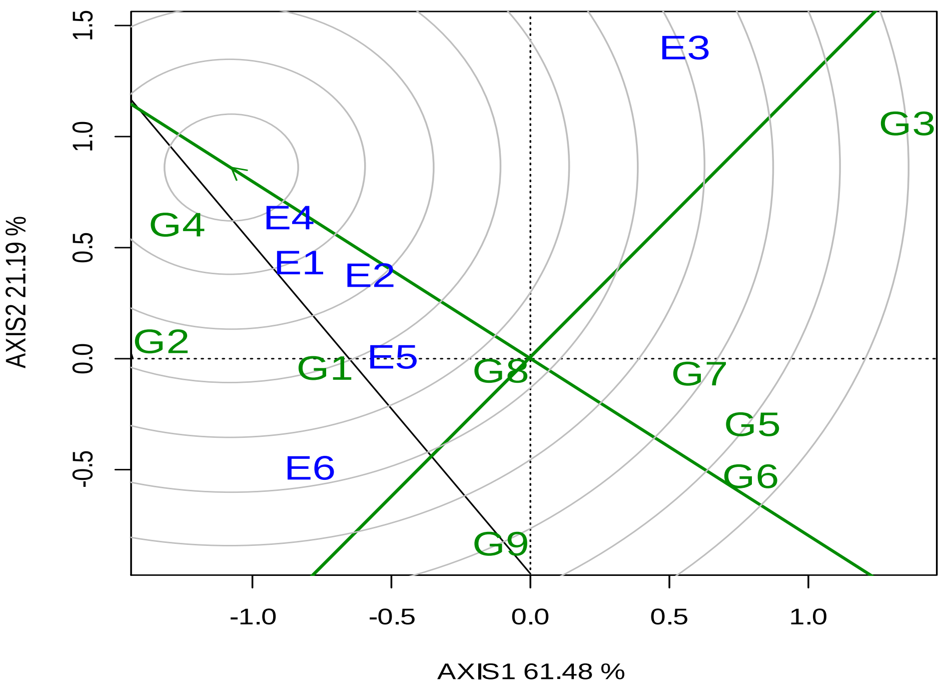

Figure 4. Ranking of genotypes based on yield performance and stability. Where: G1: Fetan, G2: Don Matteo, G3: Alemtena, G4: Tesfaye, G5: Wehabit, G6: Rigeat, G7: Utuba, G8:Mangudo, and G9: Toltu genotype (E1: Simada, E2: Bichena, E3: Dabat 2022 cropping season and E4: Simada, E5: Bichena, E6: Dabat in 2023 cropping season. The blue numbers connected to the polygon represent the genotypes, whereas the green numbers represent the locations/test environments.

3.5.4. Interrelationship among environment

In a GGE biplot, the correlation intensity between enviroment is represented by the cosine of the angle between any two environment vectors [60]. A correlation is considered positive if the angle is less than 90°, negative if it is greater than 90°, and nonexistent if it is around 90°. According to [67] and [55] there is a significant connection between the correlation coefficient of two environments and the angle between their vectors, which is utilized to categorize the test environments. Therefore, the top-yielding genotypes in one environment will perform best in other environments if two environments have a positive correlation. In the context of GGE biplot analysis,understanding the relationship between environments is crucial for interpreting genotype performance across different conditions. When two environments show a negative correlation, it suggests that genotypes performing well in one are likely to perform poorly in the other, and vice versa [55, 60]. In the present study, as shown in (**Fig. 5**), the environments Simada, and Bichena are positively correlated, as indicated by an angle their vectors less than 90°. On the other hand, the angle between Dabat and simada greater than 90°, indicating a negative correlationbetween these two environments.

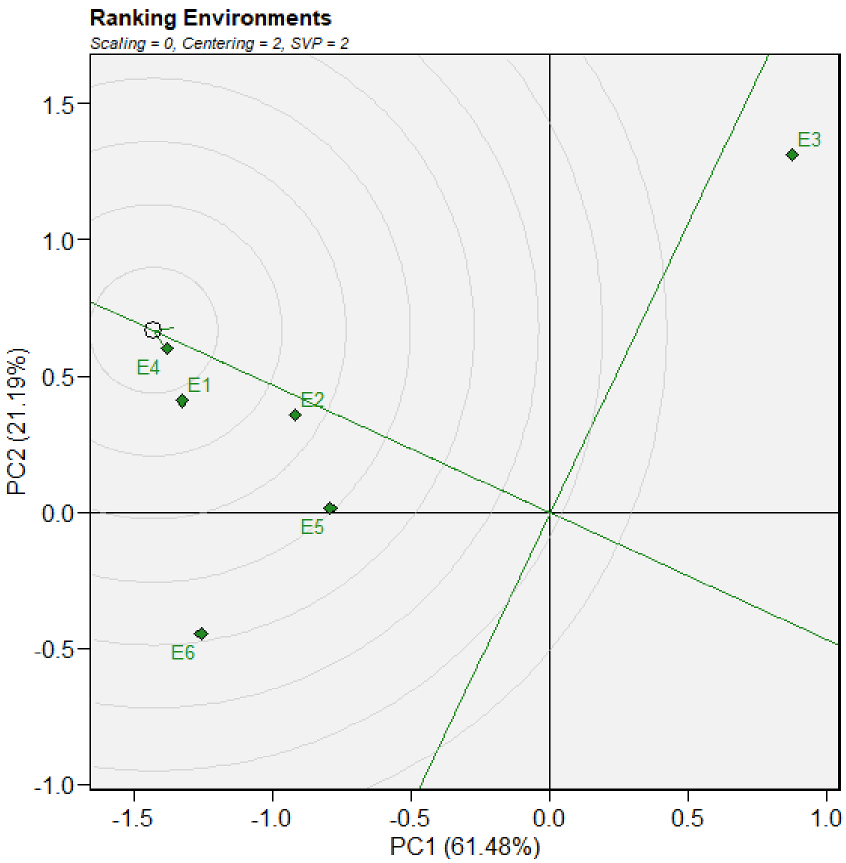

Figure 5. Ranking of environments relative to the ideal environments

Where: E1: Simada, E2: Bichena, E3: Dabat 2022 cropping season and E4: Simada, E5: Bichena, E6: Dabat in 2023 cropping season.

3.6. Stability of durum wheat genotypes

Genotypes' mean grain yield and stability performance are graphically represented using the average environment coordination (AEC) method (**Fig.6**). Combining grain yield with genotype stability performance can help identify the genotypes that yield the highest and most stable results [68]. The genotype with the highest mean performance and greatest stability across all test environments is considered ideal [69, 70]. According to the AEC view in the comparison biplot, a desirable genotype are those located close to the ideal genotype, which is typically positioned at the center of the concentric circles or at the tip of the arrow indicating the ideal point. In the AEC (Average Environment Coordination) system, the mean performance of genotypes is represented by an arrow along the AEC X-axis (PC1), which passes through the origin of the biplot and indicates the average environment axis, reflecting the overall

performance of genotypes across environments.This graphical representation is instrumental in visually distinguishing high-performing, stable genotypes from those with variable or suboptimal performance.

On the positive side of the AEC abscissa, genotypes located closer to the origin would have a higher mean grain yield. In contrast, genotypes farther away on the negative side would have a lower mean grain yield. Additionally, a genotype's projection becomes less stable the farther it is from the origin in absolute terms. This line indicates a higher mean yield across the environments. Hence, in the present study, G2 gave the highest mean yield, followed by G1, and G6. The performance of the remaining genotype groups (G3 and G5) was below the grand mean yield (**Fig. 6**). An ideal genotype is completely stable across a wide range of environments and has the highest average performance of any genotype [71].

Higher GEI interaction and lower stability are indicated by genotype that lie further from the origin along the AEC ordinate (y-axis), in either direction [72]. G4 (Tesfaye) and G2 (Don Matteo) showed high yield and stability. G8 (Mangudo) was the most stable but moderate in yield. Conversely, G3 (Alemtena) and G6 (Rigeat) showed instability and were adapted to specific environments. Although G3 (Alemtena) produced the highest mean yield in some environments, its large deviations along the AEC ordinate indicated low stability (**Fig.6**). Among environments, E2 (Simada 2022), E4 (Bichena 2023), and E1 (Bichena 2022) were located closer to the AEC abscissa, indicating representativeness, while E3 (Dabat 2022) showed the largest deviation, suggesting high discriminating ability but low representativeness.

An ideal genotype for a specific environment has the highest mean yield and responds best in that particular environment while being less stable in the other environments, thus necessitating recommendations for the specific environment [73]. Stability is reported to have lower heritability than mean performance [74, 75]. It is important to note that stability pertains to the genotype's relative performance and gains significance only when linked to the mean [76]. In summary, environmental factors (e.g., temperature, rainfall, soil quality) and genetic factors (e.g., drought resistance, nutrient efficiency) jointly influence yield-related parameters such as biomass, spike length, and the number of tillers. Wheat varieties with favorable genetic factors tend to perform better under various environmental conditions, resulting in improved overall

yield [11, 77, 78]. Genotype stability was further evaluated using different stability parameters. The figure clearly identifies G4 as the ideal genotype and E4 as the ideal environment. G1 and G2 also appear as desirable genotypes, while E1 and E2 emerge as desirable environments, suggesting they offer favorable conditions for good performance and stability.

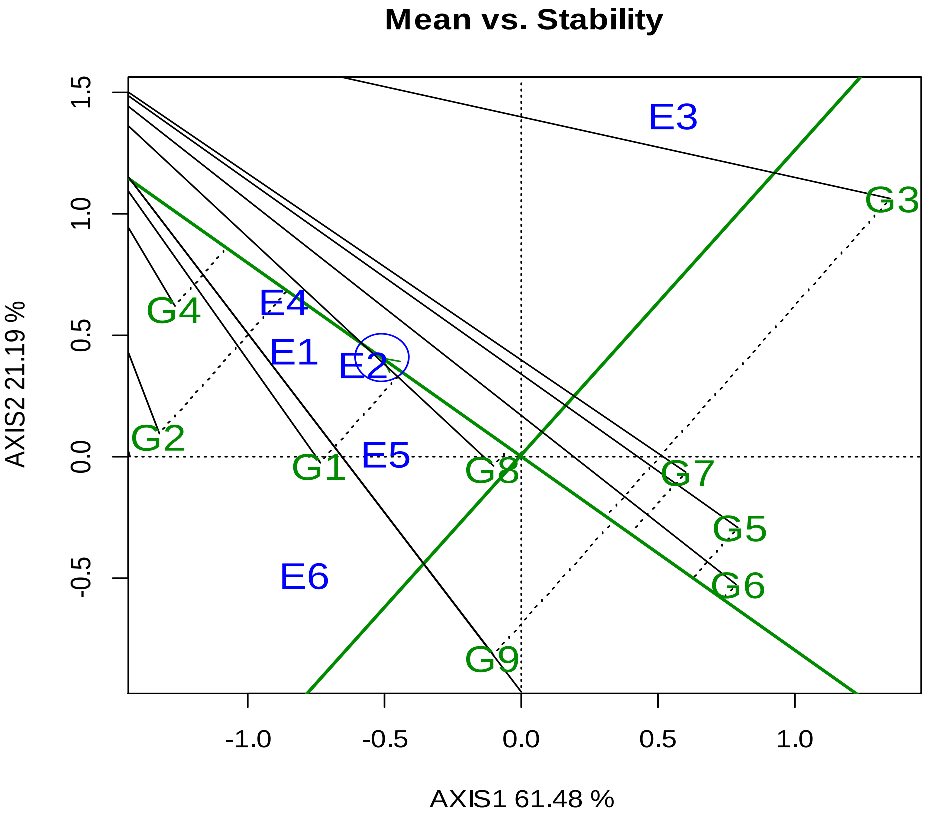

Figure 6.Which genotype was more stable across the tested environments? Where: G1: Fetan, G2: Don Matteo, G3: Alemtena, G4: Tesfaye, G5: Wehabit, G6: Rigeat, G7: Utuba, G8:Mangudo, and G9: Toltu genotype (E1: Simada, E2: Bichena, E3: Dabat 2022 cropping season and E4: Simada, E5: Bichena, E6: Dabat in 2023 cropping season. The blue numbers connected to the polygon represent the genotypes, whereas the green numbers represent the locations/test environments.

3.7. Stability parameter

3.7.1. The regression coefficient

The performance of a genotype in an environment is determined by its mean performance, environmental responsiveness as a linear function, and its deviation from regression. Eberhart and Russell suggested using a linear regression coefficient and the variance of the regression

deviations to evaluate crop responses to environmental changes. The average stability is indicated by a regression coefficient (bi) that approaches one and a deviation from regression (S2di) of zero. In this model, regression scores above one indicate varieties that are more sensitive to changes in the environment (below-average stability) and are specifically adaptable to high-yielding environments [79]. Conversely, regression coefficients below one enhance the specificity of adaptability to low-yielding environments by demonstrating greater resistance to environmental change (above-average stability).

The present results indicated that linear regression of the average grain yield of individual genotype on the average yield of all varieties in each environment produced regression coefficients (bi values) ranging from -0.012 to 1.73. This variation in regression coefficients reflects differing responses of the varieties to environmental changes (Table 7). A mean regression coefficient (bi) that is close to one, low deviation form regression, and grain yields higher than the grand mean indicate that the aforementioned varieties Fetan, Tesfaye and Utuba are well adapted to all environments, indicating broader adaptation across different environments. In contrast Toltu are poorly adapted to all environments. It is recommended that these varieties be cultivated under unfavorable conditions, as they demonstrate resilience to environmental variations. Similar finding have been reported by other researchers [80].

Table 7. The various models of stability used to partition the GEI interaction for grain yield in the test of durum wheat genotypes and their ranking

Genotypes	Mean	Rank	Bi	s^2di	σ^2	Wi	pi	ASU	YSI	Rank	s1	s2	ASI	ASV
1	3.3	3	0.84	0.14	0.12	97.70	0.11	7	10	4	0.27	7.77	0.28	1.38
2	3.51	2	1.41	0.21	0.22	102.00	0.00	8	10	3	0.33	10.70	0.40	2.00
3	3.02	6	-0.012	0.62	0.74	80.30	0.80	9	15	6	0.20	8.67	0.76	3.78
4	3.69	1	0.98	0.04	0.03	114.00	0.00	5	6	1	0.07	1.47	0.18	0.87
5	2.75	9	0.35	0.00	0.03	84.70	0.79	2	11	9	0.40	4.97	0.11	0.54
6	2.78	8	1.32	0.06	0.05	83.30	0.66	3	11	8	0.33	4.57	0.11	0.56
7	2.95	7	0.73	0.04	0.03	90.00	0.56	4	11	7	0.13	3.50	0.17	0.87
8	3.12	4	1.43	0.14	0.20	91.30	0.27	1	5	2	0.27	8.80	0.10	0.48
9	3.07	5	1.73	0.22	0.27	87.40	0.45	6	11	5	0.00	14.60	0.27	1.33

$\mathbf{S^1}$= Nassar and Huens's (1987) Mean of the absolute rank differences of a genotype over the n environments, $\mathbf{S^2}$= Nassar and Huens's (1987) variance among the ranks over the k environments, **Wi**=Wriecke's ecovalence, **Pi** =Cultivar superiority, $\boldsymbol{\sigma^2}$ = Shukla's (1972) stability variance, **bi** =Eberhart and Russell (1966) stability value of regression coefficient and $\mathbf{S^2}$**di**= Eberhart and Russell (1966) stability deviation value from regression, ASI= AMMI stability index, and ASV=AMMI stability value.

3.8. Limitation of the Study

While the findings provide valuable insights into the performance and stability of durum wheat genotypes, it is important to acknowledge certain limitations. The study is based on data collected over only two years and across six environments, which may not fully capture the extensive environmental variability affecting durum wheat production. Additionally, although the statistical methods used are robust, they carry inherent assumptions and limitations that could influence the interpretation of genotype-by-environment interactions.Therefore, recommendations for commercial cultivation should be made with caution. Conducting further multi-year and multi-location trials would enhance the reliability and generalizability of these results.

4. Conclusions and Recommendation

Analysis of GEI interaction in this study highlighted the importance of evaluating durum wheat genotypes across multiple environments to determine their yield stability and adaptability. The combined analysis of variance over the years showed significant difference among genotypes, environments, and their interaction, confrining that both genetic and environmental factors significantly influenced grain yield and other agronomic traits. Genotype Tesfaye, followed by Don Matteo, consistently exhibited superior phenotypic performance across all traits,with grain yields of 3.74 and 3.62 tha^{-1}, respectively, making them the top-yielding genotypes. Grain yield across test environments varied substantially, ranging from 4.5 t ha^{-1} in Dabat to 2.29 t ha^{-1} in Simada.The first principal component (PC1) contributed significantly to the interaction, accounting for 69.76%, whereas the second principal component (PC2) explained an additional 21.71%, together accounting for 91.47% of the genotype-by-environment interaction. AMMI and GGE biplot analyses effectively visualized the GEI patterns, grouping the six test environments and nine genotypes into two mega-environments and six genotype clusters. Simada and Bichena emerged as both highly discriminating and representative environments, making them ideal sites for evaluating genotype performance. Grain yield analysis showed that Tesfaye (G4) and Don Matteo (G2) consistently achieved the highest yields and broad adaptability, whereas Wehabit (G5) and Rigeat (G6) performed poorly, reflecting limited stability. AMMI and GGE biplot analyses confirmed significant GEI, with environments explaining most of the variation. AMMI

identified Tesfaye and Don Matteo as high-yielding and stable genotypes, while Alemtena (G3) and Rigeat (G6) showed instability and environment-specific adaptation. Based on these results, it is recommended to cultivate Tesfaye and Don Matteo in the Amhara region and other similar durum wheat-growing areas, given their high yield and moderate to high stability. Additionally, Simada and Bichena should be prioritized as core testing sites in future multi-environment trials due to their strong discriminative and representative capacities. Continued exploration of GEI is essential to refine varietal recommendations and ensure optimal wheat production in specific agro-ecological zones.

Conflict of interests

The authors have not declared any conflict of interest.

Authors' contribution statement

A.E: Wrote the original draft, data collected and analyzed, investigated, revised, and edited; **A. A** : Restructured, reviewed, collected the data, and analyzed; **Y.A**: Contributed to reviewing, data collection, and editing, **M.A:** Restructured, Contributed to reviewing, data collection and editing and **K.N:** Restructured, Contributed to reviewing, data collection and editing All authors read and approved the final version of the manuscript.

Ethics approval and consent to participate

Not applicable

Consent for publication

Not applicable

Availability of data and material

Datasets generated in this study are available from the corresponding author upon reasonable request.

Funding statement:

No found source

5. REFERENCES

[1] Tilahun WM, Bekeko Z, Silesh F. IMPORTANCE OF WHEAT STEM RUST DISEASE (Puccinia graminis f. sp. tritici) IN NORTH SHEWA, ETHIOPIA, AND RESISTANCE OF DURUM WHEAT LANDRACE ACCESSIONS [Triticum turgidum subsp. durum (Desf.) Husn.] TO THE PREVAILING RACES OF THE PATHOGEN: Haramaya University; 2022.
[2] Yang F, Zhang J, Liu Q, Liu H, Zhou Y, Yang W, et al. Improvement and re-evolution of tetraploid wheat for global environmental challenge and diversity consumption demand. International journal of molecular sciences. 2022;23:2206.
[3] Vavilov NI. The origin, variation, immunity and breeding of cultivated plants: LWW; 1951.
[4] Kebede M, Temam B, Getahun M, Tsegaye Y. Evaluation of durum wheat (Triticum turgidum) varieties against stem rust (Puccinia graminis f. sp. tritici) in selected districts of southern Ethiopia. 2020.
[5] Hodson D, Jaleta M, Tesfaye K, Yirga C, Beyene H, Kilian A, et al. Ethiopia's transforming wheat landscape: tracking variety use through DNA fingerprinting. Scientific reports. 2020;10:18532.
[6] Baranski M. The wide adaptation of green revolution wheat: Arizona State University; 2015.
[7] Tidiane Sall A, Chiari T, Legesse W, Seid-Ahmed K, Ortiz R, Van Ginkel M, et al. Durum wheat (Triticum durum Desf.): Origin, cultivation and potential expansion in Sub-Saharan Africa. Agronomy. 2019;9:263.
[8] Dibaba R. Wheat production, marketing and consumption in Ethiopia. Journal of Marketing and Consumer Research. 2019;55:10-9.
[9] Shiferaw B, Smale M, Braun H-J, Duveiller E, Reynolds M, Muricho G. Crops that feed the world 10. Past successes and future challenges to the role played by wheat in global food security. Food security. 2013;5:291-317.
[10] Hailu F. Farmers perception of pesticide use and genetic erosion of landraces of tetraploid wheat (Triticum spp.) in Ethiopia. Genetic resources and crop evolution. 2017;64:979-94.
[11] Eskezia A, HK, MA. Grain yield Performance Evaluation of Bread Wheat (Triticum aestivum L.) Varieties in

East Gojjam Zone. Advanced Journal of Interdisciplinary Studies. 2024;8(2):1663-76.
[12] Minot N, Warner J, Lemma S, Kasa L, Gashaw A, Rashid S. The wheat supply chain in Ethiopia: Patterns, trends, and policy options. Gates Open Res. 2019;3:174.
[13] Abdalla A, Stellmacher T, Becker M. Trends and prospects of change in wheat self-sufficiency in Egypt. Agriculture. 2022;13:7.
[14] Cappelli A, Cini E. Challenges and opportunities in wheat flour, pasta, bread, and bakery product production chains: A systematic review of innovations and improvement strategies to increase sustainability, productivity, and product quality. Sustainability. 2021;13:2608.
[15] ESS. Ethiopian Statistical Services). 2022. Report on Area and Production of Major Crops (private peasant holdings, Meher season). Volume I, Statistical Bulletin 593, Addis Ababa, Ethiopia. 2022.
[16] Anteneh A, Asrat D. Wheat production and marketing in Ethiopia: Review study. Cogent Food & Agriculture. 2020;6:1778893.
[17] Badebo A, Gelalcha S, Ammar K, Nachit M, Abdalla O. Overview of durum wheat research in Ethiopia: challenges and prospects. 2009.
[18] Tadesse W, Zegeye H, Debele T, Kassa D, Shiferaw W, Solomon T, et al. Wheat production and breeding in Ethiopia: retrospect and prospects. Crop Breeding, Genetics and Genomics. 2022;4.
[19] Zenebe M. Assessment and Management of Wheat Stem Rust (Puccinia graminis tiritici) Using Fungicides and Wheat Varietiesin Meket District, North Wollo, Ethiopia 2022.
[20] Mekuriaw A. Agroecology-specific varietal matching to achieve self-sufficiency in wheat production in Ethiopia. Heliyon. 2023;9.
[21] Yismaw D. GENOTYPE BY ENVIRONMENT INTERACTION DURUM WHEAT (Triticum turgidum GROWING AREAS OF GONDAR AND NORTH SHOA GRADUATE PROGRAM AND STABILITY ANALYSIS ssp.

durum) GENOTYPES IN ZONES OF AMHARA REGION, ETHIOPIA M. Sc. Thesis By Yismaw Degenet Mogess November Bahir Dar, Ethiopia OF WHEAT 2019 2019.
[22] Mulugeta B, Tesfaye K, Geleta M, Johansson E, Hailesilassie T, Hammenhag C, et al. Multivariate analyses of Ethiopian durum wheat revealed stable and high yielding genotypes. PloS One. 2022;17:e0273008.
[23] Tinker N, Yan W. Information systems for crop performance data. Canadian Journal of Plant Science. 2006;86:647-62.
[24] Yan W, Tinker NA, Bekele WA, Mitchell-Fetch J, Fregeau-Reid J. Theoretical unification and practical integration of conventional methods and genomic selection in plant breeding. Crop Breeding, Genetics and Genomics. 2019;1.
[25] Tesfaye A, Bogale A, Namara RE, Bacha D. The impact of small-scale irrigation on household food security: The case of Filtino and Godino irrigation schemes in Ethiopia. Irrigation and Drainage Systems. 2008;22:145-58.
[26] Kebede G, Worku W, Feyissa F, Jifar H. Genotype by environment interaction and stability analysis for selection of superior fodder yield performing oat (Avena sativa L.) genotypes using GGE biplot in Ethiopia. Ecological Genetics and Genomics. 2023;28:100192.
[27] Mwila C. Grain yield stability, genetic gain and path coefficient analyses in advanced soybean (Glycine max (L.) Merr.) lines 2017.
[28] Wodebo KY, Tolemariam T, Demeke S, Garedew W, Tesfaye T, Zeleke M, et al. AMMI and GGE biplot analyses for mega-environment identification and selection of some high-yielding oat (Avena sativa L.) genotypes for multiple environments. Plants. 2023;12:3064.
[29] El-Aty MSA, Abo-Youssef MI, Sorour FA, Salem M, Gomma MA, Ibrahim OM, et al. Performance and stability for grain yield and its components of some rice cultivars under various environments. Agronomy. 2024;14:2137.
[30] Mullualem D, Tsega A, Mengie T, Fentie D, Kassa Z, Fassil A, et al. Genotype-by-environment interaction and stability analysis of grain yield of bread wheat (Triticum aestivum L.) genotypes using AMMI and GGE biplot analyses. Heliyon. 2024;10.
[31] Evlice AK, Pehlivan A, Şanal T, Canay FÜ, Avcıoğlu R, Keskin Ş, et al. Assessment of durum wheat (Triticum durum Desf.) genotypes for grain yield and quality in breeding programmes under rainfed conditions. INDIAN JOURNAL OF GENETICS AND PLANT BREEDING. 2025;85:68-77.
[32] Tulu L, Wondimu A. Adaptability and yield stability of bread wheat (Triticum aestivum) varieties studied using GGE-biplot analysis in the highland environments of South-western Ethiopia. African Journal of Plant Science. 2019;13:153-62.
[33] Tekle HT, Tsehaye Y, Atsbeha G, Abera FA, Chiulele RM. Investigation of genotype x environment interaction for Hordeum vulgare L. ssp. vulgare recombinant inbred lines in multi-environments of Tigray, Ethiopia. Ecological Genetics and Genomics. 2024;31:100231.
[34] Kaloki P, Trethowan R, Tan DK. Effect of genotype× environment× management interactions on chickpea phenotypic stability. Crop and Pasture Science. 2019;70:453-62.
[35] Shukla G. Some statistical aspects of partitioning genotype-environmental components of variability. 1972.
[36] Finlay , Wilkinson. The analysis of adaptation in a plant-breeding programme. Australian journal of agricultural research. 1963;14:742-54.
[37] Carvalho RF, Silva TS, Pereira AKdS, Cavallini GS, Pereira DH. Theoretical and Experimental Study of the Stability of Thiamethoxam Under Different Environmental Conditions. Processes. 2024;12:2328.
[38] Fox J, Weisberg S. Using car and effects Functions in Other Functions. Published online. 2020;1.
[39] Dumble S, Frutos Bernal E, Galindo V. Package GGEBiplots. Version 01. 2022;3.

[40] Hilmarsson HS, Rio S, Sánchez JIy. Genotype by environment interaction analysis of agronomic spring barley traits in Iceland using AMMI, factorial regression model and linear mixed model. Agronomy. 2021;11:499.
[41] Francis T, Kannenberg L. Yield stability studies in short-season maize. I. A descriptive method for grouping genotypes. Canadian journal of plant science. 1978;58:1029-34.
[42] Purewal SS, Kaur P, Salar RK. Chickpea and Cowpea: Nutritional Profile, Processing, Health Prospects and Commercial Uses: CRC Press; 2023.
[43] Wricke G. Uber eine methode zur erfassung der okologischen streubreite in feldversucen. z Pflanzenzuchtung. 1962;47:92-6.
[44] Eberhart St, Russell W. Stability parameters for comparing varieties 1. Crop science. 1966;6:36-40.
[45] Olivoto T, Lúcio ADC. metan: An R package for multi-environment trial analysis. Methods in Ecology and Evolution. 2020;11:783-9.
[46] Delesa A, Dabi A, Alemu G, Geleta N, Solomon T, Zegeye H, et al. Stability and performance evaluation of advanced bread wheat (Triticum aestivum L.) genotypes in low to mid altitude areas of Ethiopia. International Journal of Bio-resource and Stress Management. 2023;14:19-32.
[47] Gebrie G, Abebe D. Adaptation and performance evaluation of bread wheat (Triticumaestivum L.) varieties in Guagusa-shikudad District of North western Ethiopia. Asian J Plant Sci Res. 2020;10:64-8.
[48] Birhanu A, Tahir Z, Tilahun M, Tilahun M, Hailu A. Performance Evaluation and Adaptation of Food Barley (Hordeum Vulgare L.) Varieties in the Highlands of North Gondar Ethiopia. American Journal of Agriculture and Forestry. 2020;8:252-7.
[49] Dabi A, Alemu G, Sime B, Geleta N, Delessa A, Solomon T, et al. Genotype× Environment interaction and stability analysis using GGE biplot for grain yield of bread wheat (Triticum aestivum) genotypes under low moisture stress areas of Ethiopia. International Journal of Bio-resource and Stress Management. 2023;14:1089-98.
[50] Rharrabti Y, del Moral LG, Villegas D, Royo C. Durum wheat quality in mediterranean environments: III. Stability and comparative methods in analysing G× E interaction. Field Crops Research. 2003;80:141-6.
[51] Assefa A, Girmay G, Alemayehu T, Lakew A. Performance evaluation and stability analysis of malt barley (Hordeum vulgare L.) varieties for yield and quality traits in Eastern Amhara, Ethiopia. CABI Agriculture and Bioscience. 2021;2:31.
[52] Alemu YA, Anley AM, Abebe TD. Genetic variability and association of traits in Ethiopian durum wheat (Triticum turgidium L. var. durum) landraces at Dabat Research Station, North Gondar. Cogent Food & Agriculture. 2020;6:1778604.
[53] Kassie MM, Abebe TD, Desta EA, Tadesse W. Multi-Environment Evaluation of Bread Wheat Genotypes for Yield Stability in Ethiopia Using AMMI and GGE-Biplot Analyses. Crop Breeding, Genetics and Genomics. 2025;7.
[54] Chimdesa O, Asefa K, Alemu S, Teshome G. Evaluating Agronomic Performance and Yield Stability of Improved Bread Wheat Varieties across Low Moisture Stress Areas of Guji Zone, Southern Oromia. International Journal of Research in Agriculture and Forestry. 2019;6:1-10.
[55] Yan W, Kang MS, Ma B, Woods S, Cornelius PL. GGE biplot vs. AMMI analysis of genotype-by-environment data. Crop science. 2007;47:643-53.
[56] Tadesse Z, Habtemariam Zegeye DA, Tafesse Solomon YS, Debele T. Identification of Stable Bread Wheat (Triticum Aestivum L.) Genotypes using AMMI Analysis in Ethiopia. Int J Res Agric For. 2018;5:6-14.
[57] Mohammadi R, Amri A. Analysis of genotype× environment interaction in rain-fed durum wheat of Iran using GGE-biplot and non-parametric methods. Canadian Journal of Plant Science. 2012;92:757-70.
[58] Pour-Aboughadareh A, Barati A, Koohkan SA, Jabari M, Marzoghian A, Gholipoor A, et al. Dissection of genotype-by-environment interaction and yield stability analysis in barley using AMMI model and stability statistics. Bulletin of the National Research Centre. 2022;46:19.

[59] Mohammadi M, Sharifi P, Karimizadeh R, ALT JAFARBY J, Khanzadeh H, Hosseinpour T, et al. Stability of grain yield of durum wheat genotypes by AMMI model. Agriculture & Forestry/Poljoprivreda i šumarstv. 2015;61.
[60] Yan W, Tinker NA. Biplot analysis of multi-environment trial data: Principles and applications. Canadian journal of plant science. 2006;86:623-45.
[61] Gauch Jr HG, Zobel RW. Identifying mega-environments and targeting genotypes. Crop science. 1997;37:311-26.
[62] Scavo A, Mauromicale G, Ierna A. Dissecting the genotype× environment interaction for potato tuber yield and components. Agronomy. 2022;13:101.
[63] Khare V, Shukla RS, Pandey S, Singh SK, Singh C. Exploring the genotype-environment interaction of bread wheat in ambient and high-temperature planting conditions: a rigorous investigation. Scientific reports. 2024;14:2402.
[64] Demelash H. Genotype by environment interaction, AMMI, GGE biplot, and mega environment analysis of elite Sorghum bicolor (L.) Moench genotypes in humid lowland areas of Ethiopia. Heliyon. 2024;10.
[65] Wardofa GA, Asnake D, Mohammed H. Gge biplot analysis of genotype by environment interaction and grain yield stability of bread wheat genotypes in central ethiopia. Journal of plant breeding and genetics. 2019;7:75-85.
[66] Adem Wado D. GENOTYPE X LOCATION X PLANTING PATTERN INTERACTION AND STABILITY ANALYSIS OF SORGHUM [Sorghum bicolor (L.) Moench] VARIETIES IN DRYLANDS OF ETHIOPIA: Haramaya university; 2020.
[67] Yan F, Polk DB. Probiotic bacterium prevents cytokine-induced apoptosis in intestinal epithelial cells. Journal of biological chemistry. 2002;277:50959-65.
[68] Mullualem D, Tsega A, Mengie T, Fentie D, Kassa Z, Fassil A, et al. Genotype-by-environment interaction and stability analysis of grain yield of bread wheat (Triticum aestivum L.) genotypes using AMMI and GGE biplot analyses. Heliyon. 2024;10:e32918.
[69] Vaezi B, Pour-Aboughadareh A, Mohammadi R, Mehraban A, Hossein-Pour T, Koohkan E, et al. Integrating different stability models to investigate genotype× environment interactions and identify stable and high-yielding barley genotypes. Euphytica. 2019;215:1-18.
[70] Wagaw K, Seyoum A, Tadesse T, Nega A, Gebreyohannes A, Bejiga T, et al. Distinguishing of stable genotypes and mega environment for grain yield performance of sorghum [Sorghum bicolor (L.) moench] genotypes using spatial analysis. American Journal of Plant Sciences. 2021;12:417-31.
[71] Bekana G. Genotype X Environment Interactions and Yield Stability of Tef (Eragrostis tef) Genotypes Grown in Central Parts of Ethiopia. American Journal of BioScience. 2023;11:104-10.
[72] Kaya Y, Akçura M, Taner S. GGE-biplot analysis of multi-environment yield trials in bread wheat. Turkish journal of agriculture and forestry. 2006;30:325-37.
[73] Abraha MT, Shimelis H, Solomon T, Hailu A. Genotype-by-environment interaction and selection of elite wheat genotypes under variable rainfall conditions in northern Ethiopia. Journal of Crop Improvement. 2019;33:797-813.
[74] Eskezia A, Kefale H, Asrat M. Genotype by environment interaction and yield stability analysis of bread wheat (Triticum aestivum L.) varieties in East Gojjam Zone, Northwest Ethiopia. Heliyon. 2025;11.
[75] Nadim MKA, Rahman M, Sikder MR, Adhikary S, Atiq NB, Alam MM, et al. Performance, adaptability, and multi-trait stability of rice varieties (Oryza sativa L.) in rainfed conditions. Agrosystems, Geosciences & Environment. 2025;8:e70038.
[76] Atsbeha G, Mekonnen T, Kebede M, Haileselassie T, Tesfaye K. Multivariate analyses, heritability and genotype environment interaction of bread wheat genotypes in Ethiopia. Ecological Genetics and Genomics. 2023;29:100209.

[77] Alemu G, Dabi A, Geleta N, Duga R, Solomon T, Zegaye H, et al. Genotype× environment interaction and selection of high yielding wheat genotypes for different wheat-growing areas of Ethiopia. Am J Biosci. 2021;9:63-71.
[78] Gebrewahid L, Mengistu DK, Tsehaye Y, Aberha A, Aberra DA. Variability among Ethiopian durum wheat genotypes grown under different climatic conditions of Tigray for some agronomic and grain-quality traits. Journal of Crop Improvement. 2021;35:184-203.
[79] Mansour E, Moustafa ES, El-Naggar NZ, Abdelsalam A, Igartua E. Grain yield stability of high-yielding barley genotypes under Egyptian conditions for enhancing resilience to climate change. Crop and Pasture Science. 2018;69:681-90.
[80] Delesa A, Alemu G, Geleta N, Dabi A, Zegeye H, Solomon T, et al. Stability and performance evaluation of advanced bread wheat (Triticum aestivum L.) genotypes in optimum areas of Ethiopia. International Journal of Bio-resource and Stress Management. 2022;13:69-80.

Manuscript. Number: PONE-D-27-30070

Journal: Plos one

Title: Genotype by Environment Interaction and Yield Stability Analysis of Durum Wheat (*Triticum turgidum* var. *durum*) Varieties Using AMMI and GGE biplot Analysis in Amahara Region, Northwest Ethiopia

Dear Editor and Reviewers

We would like to thank both the editor and the reviewers for your contribution to this manuscript by filtering out the technical and editorial errors in the scientific research writing that could increase the reliability and scientific exchange of the research output. We also admire your effort to see the details of our manuscript taking your golden time and we value all the comments. Further, we would be very glad to publish our manuscript in the Plos one journal if accepted. We believe that we have addressed all comments as suggested in the text and point-by-point response. Hence, kindly consider our revisions accordingly.

Response to the comments

Title section reviewer comment.

Title: needs minor modification and I suggest it to be;

"Genotype by Environment Interaction and Yield Stability Analysis of Durum Wheat (*Triticum turgidum* var. *durum*) Varieties Using AMMI and GGE biplot Analysis **in Amahara state or Region**, Northwest Ethiopia".

Response_ Thank you for your valuable comments. We have modified our manuscript title based on the reviewer's feedback and suggestions.

Genotype by Environment Interaction and Yield Stability Analysis of Durum Wheat (*Triticum turgidum* var. *durum*) Varieties Using AMMI and GGE biplot Analysis in Amahara Region, Northwest Ethiopia

Abstract section reviewer comment.

Abstract: this section needs major modification;

1. Abstract poorly composed though the study has good results.

Response_ We have updated the section by incorporating the main results into the abstract.

2. It should be written again because some data presented there has mistakes

   Response: We have rewritten and updated the entire abstract based on the study results.

3. Some important result information not presented

   Response_ We have updated it

4. There are lots of grammatical & typographical mistakes

   Response_ All grammatical and typographical errors have been corrected as per the reviewer's comment.

5. I tried to correct some of them using the MSWord track review method in the attached file

Response_ Thank you for your valuable comments. We have reviewed them and made the necessary corrections.

6. The AMMI analysis Anova output was reported as the result of combined anova result

   Response_ We have corrected the AMMI analysis ANOVA output to accurately present it separately from the combined ANOVA results.

<u>Introduction section reviewer comment</u>.

1. It has good information but presented poorly, it is highly disorganized, paragraphs should be reshuffled.
   Response_ the necessary corrections have been made, and the paragraphs have been reorganized to improve clarity and structure.
2. In text citations didn't follow the journal's guideline
   Response_ In-text citations have been revised in accordance with the journal's guidelines.
3. Lots of sentences not clearly written. Several of them need to be rewritten
   Response_ We have rewritten unclear sentences to improve clarity and readability

4. Some topics like AMMI & GGE described repeatedly about their use and assumptions in detail, but no previous literatures on durum wheat presented that show their practical applications
   Response_ We have revised the sections on AMMI and GGE to reduce repetition and included relevant previous literature on their applications in related crops.
5. There is no statement or justifications why the authors selected the AMMI & GGE biplot models
   Response_ We have added a clear justification for selecting the AMMI and GGE biplot models, highlighting their suitability for analyzing genotype by environment interactions in our study
6. Problem statement or the need to do this study not described sufficiently
   Response_ The problem statement has been strengthened to provide a more comprehensive justification for conducting this study.
7. Objectives of the research not stated clearly
   Response _ We have revised the objectives section to clearly and explicitly state the research goals

Materials & methods section reviewer comment.

1. the sub-title 2.1 should be modified; here you are giving the detail account of the study (experimental) area

Response _ we update it

2. Table 1.Geographical description of the study area; It would be more informative if you can incorporate air temperature, and rainfall data of each year for the specific locations for the specific years. Specific geographic coordinates (including altitude) of the trial sites & if possible soil physic-chemical properties.
   Response _ We have included air temperature and rainfall data for each year and the specific geographic coordinates, including altitude, of the trial sites. However, soil physicochemical properties were not available and thus could not be included
3. 2.2. Experimental material and design; first 4-5 line needs to be rewritten

   Response-We have rewritten the first 4–5 lines of section 2.2 to improve clarity and coherence
4. Table 3; Can you include their pedigree for each variety? You better include their maturity period, yielding ability, areas of adaptation (recommendation), level of disease resistance, etc.
   Response- We have included the pedigree, maturity period, yielding ability, recommended areas of adaptation, and disease resistance levels for each variety in Table 3 as suggested.
5. 2.3. Data Collected; you have not described how you collected the data. At least you can insert citations of a published material that explains how you collected the data.

So that readers get knowledge of how to do similar experiments using same or different materials.

Response- We have included a detailed description of the data collection methods along with relevant citations to guide readers on how to conduct similar experiments.

6. GGE biplot analysis- Which method or formula did you use? You need to show the formula you have used to compute the analysis

   Response- The manuscript now includes a detailed description of the GGE biplot analysis method along with the corresponding formula, as requested

<u>Results and discussions section reviewer comment</u>

- Same problem was observed in the in text citation as above

1. 3.2. Performance of genotypes - What about the performance of these durum wheat genotypes in previous studies by other researchers? Please cite previous works that either agree or contradict with your findings to make your discussion complete
   Response- Relevant literature discussing the performance of durum wheat genotypes in previous studies has been cited to contextualize and strengthen the discussion of our results.
2. What is this MSE? Is it the std of the mean values?
   Response-Its MSE (Mean Square Error) is not the standard deviation.
3. 3.3. Genotype performance at each tested location- this sub-topic also lacks supporting literatures for the findings presented similar to sub-topic 3.2
   Response- We have added relevant supporting literature to the sub-topic on genotype performance at each tested location.
4. 3.4. Additive Main Effects and Multiplicative Interaction (AMMI) Analysis- One table is missing from AMMI analysis; the significant IPCAs (usually 1&2) give vector values for each genotype that indicates how much each genotype contributes to the GEI. Absolute value of the IPCA score determines its stability. Low value close to zero indicates the genotype being stable. You didn't indicate which genotypes are stable using the AMMI analysis either using the table or the AMMI biplot 1 graph.
   Response - We have included the AMMI analysis results by adding Table 7, which presents YSI, ASV, IPCA1, and IPCA2 values. Based on these, we have identified and indicated the stable genotypes as per the reviewer's suggestion.
5. Please refer the following publication for better understanding of the results and interpretation of AMMI analysis; Kassie MM, Abebe TD, Desta EA, Tadesse W. Multi-Environment Evaluation of Bread Wheat Genotypes for Yield Stability in Ethiopia Using AMMI and GGE-Biplot Analyses. Crop Breed Genet Genom. 2025;7(2):e250004.

Response - Thank you for the helpful reference. We have reviewed the suggested publication by Kassie et al. (2025) and incorporated relevant insights to enhance the interpretation and discussion of the AMMI analysis results in our manuscript.

6. It appears that there is error in the table numbering, check it, see the comment in the manuscript file

   Response - We have carefully reviewed the manuscript and corrected the table numbering errors as pointed out.

7. On page 17, the values you presented from table 7 about the proportions of the sum of squares of the AMMI anova seems to be incorrect. In addition, another table that lists IPCA scores for each genotype & even environments is missing. The IPCA scores help in identifying the relative contribution of genotypes for the GEI determines which genotype is stable/unstable. Please read the paper Kassie, et al (2025) and some recently published papers in durum wheat by Liyew et al. (2025) too.

   Response -We have corrected the values from Table 7 regarding the sum of squares proportions in the AMMI ANOVA and added the IPCA scores for each genotype and environment as subcomponents within Table 7. This addition facilitates identification of genotype stability and contribution to GEI. We also reviewed and incorporated insights from Kassie et al. (2025) and Liyew et al. (2025) to strengthen our analysis.

8. Figure 3, the mega environment grouping not clearly displayed

   Response – We have updated Figure 3 to clearly display the mega-environment groupings, making them easier to identify

9. Figure 4, concentric circles not visible

   Response – We have enhanced Figure 4 to improve the visibility of the concentric circles as per your comment

10. Page 26, there is unfinished sentence; either correct it or delete it.

    Response – he unfinished sentence on page 26 has been corrected to ensure clarity and completeness

11. 3.7 stability parameter; here, you have produced a beautiful table (**the last table**) for the stability analysis, but it was not exploited fully. You explained only one of the stability models that favors high grain yield. Please use the most recent stability models too like ASV, YSI

    Response –Thank you for the valuable suggestion. We have expanded the stability analysis section to include and discuss additional recent stability models such as ASV and YSI, providing a more comprehensive interpretation of the results

12. References; Please use the journal's guideline for in text citations & style of referencing

    Response – We have carefully revised the in-text citations and reference list to fully comply with the journal's guidelines
