# Supplementary material for: Genotype by environment interaction and yield stability analysis of durum wheat (Triticum turgidum var. durum) varieties using AMMI and GGE biplot analysis in Amahara Region, Northwest Ethiopia
Source: PLoS One. 2026 Mar 13;21(3):e0342401. doi: 10.1371/journal.pone.0342401 (PMC12987450; doi:10.1371/journal.pone.0342401)
Supplement: Attachment — Submitted filename: Durum wheat manuscript-rv2.docx [file pone.0342401.s004.docx]

**Genotype by Environment Interaction and Yield Stability Analysis of Durum Wheat** (**Triticum turgidum var. durum**) **Varieties Using AMMI and GGE biplot Analysis in Amahara Region, Northwest Ethiopia**

Alemnesh Eskezia*^1,^ Alamir Ayenew,^2^ Yohannes Azene^3^, Mulugeta Ayitenew^1,^ Kelemu Nakachew ^1^

^1^Department of Plant Science, College of Agriculture and Natural Resources, Debre Markos University, P.O.Box 269. Ethiopia.

^2^Fogera National Rice Research and Training Center, Ethiopian Institute of Agricultural Research, Ethiopia.

^3^Gonder Agricultural Research Center, P.O.Box 1337, Gondar, Ethiopia

Corresponding author contact: [alemnesh_eskezia@dmu.edu.et](mailto:alemnesh_eskezia@dmu.edu.et). Phone number: +251918314065

**Abstract**

Durum wheat is an important crop for over a third of the global population; however, its production is challenging due to limited adaptable varieties, poor management practices, and environmental factors. New wheat varieties often fail in unfamiliar environments, leading to crop losses. To address this issue, a study was conducted to evaluate the adaptability, performance, and yield stability of recently released high-performing durum wheat cultivars in the Amahara region. The field trials were conducted over two years (2022/2023 and 2023/2024) in three districts ; Simada, Bichena, and Dabat using a Randomized Complete Block Design with three replications. Data was collected on main agronomic traits including days to heading, maturity, plant height, tiller number, spike length, spikelets per spike, biomass, and grain yield. The combined analysis of variance for grain yield showed highly significant effects (p< 0.001) due to genotype (16.6%), environment (29.78%), and genotype x environment interaction (16.84%). Grain yield varied from 4.5 t ha^-1^in Dabat to 2.29 t ha^-1^in Simada. Among the genotypes, Tesfaye had the highest mean yield (3.74 t ha^-1^), while Wehabit had the lowest (2.75 t ha^-1^). The Genotype and Genotype × Environment (GGE) biplot and Additive Main Effect and Multiplicative Interaction (AMMI) analysis identified Tesfaye, Fetan, and Rigeat as stable genotypes, while Simada and Dabat as representative environments The GGE biplot analysis grouped the six test environments and nine genotypes into three mega-environments and six genotype groups. . This study identified that cTesfaye most productive and stable wheat variety in the study area and suggested farmers should adopt it to increase wheat yield per unit area of land.

**Keywords**: AMMI, GGE, GIE, ASV, PCA, Stability, Wheat, YSI

# INTRODUCTION

Durum wheat (*Triticum turgidum var. durum*) is an Indigenous tetraploid (2n = 4x = 28) wheat species that has been cultivated in Ethiopia since ancient times (Tilahun, Bekeko, & Silesh, 2022). The country is considered one of the centers of diversity for tetraploid wheat (Vavilov, 1951; Yang *et al.*, 2022). Traditionally, this crop is produced in heavy black clay soils (vertisols) under rain-fed conditions at altitudes ranging from 1800 to 2800 meters above sea level (M. Kebede, Temam, Getahun, & Tsegaye, 2020). The proportion of durum wheat in the total wheat-growing area has declined significantly due to the introduction of semi-dwarf, fertilizer-responsive bread wheat varieties, resulting in a shift where bread wheat now occupies the largest share of production (Baranski, 2015; Hodson *et al.*, 2020). However, the area dedicated to durum wheat is expected to increase due to rising urban demand for pasta products and the expansion of irrigated wheat production in Ethiopia (Dibaba, 2019; Tidiane Sall *et al.*, 2019).

Wheat is essential for Ethiopia's agricultural development and food security because it is a significant source of protein and energy (Eskezia, 2024; Hailu, 2017; Shiferaw *et al.*, 2013). Although wheat is a staple crop for approximately one-third of the world's population, Ethiopia must expand its wheat production to achieve food self-sufficiency (Abdalla, Stellmacher, & Becker, 2022; Minot *et al.*, 2019). Wheat is crucial to the country's agricultural plans and sustainable development initiatives because of its nutritional value and flexibility (Cappelli & Cini, 2021).

Durum wheat breeding programs primarily utilize elite lines from various sources, which often exhibit differing performances across environments. This variability is largely attributed to genotype-by-environment interaction (GEI) (Mulugeta *et al.*, 2022; Yismaw, 2019). Therefore, varieties must be tested across multiple environments to assess their performance for key traits and determine their stability (Tinker & Yan, 2006; Yan, Tinker, Bekele, Mitchell-Fetch, & Fregeau-Reid, 2019). A stable genotype is defined as one that delivers a high yield with minimal performance fluctuations across diverse environments (Abtew, Lakew, Haussmann, & Schmid, 2015; Beres *et al.*, 2020; Paul *et al.*, 2016). However, breeders face significant challenges in making selection decisions due to the complexities of GEI. Thus, proper analysis of multi-environment trial data (METs) and leveraging genotypes that perform differently across environments are essential for advancing breeding programs (Hassani, Mahmoudi, Saremirad, & Taleghani, 2023).

Plant breeding programs conduct multi-environment trials to assess genotype performance under diverse environmental conditions and identify the best-performing candidates for release as varieties. **Genotype-by-environment interaction (GEI)** refers to the inconsistent or varying phenotypic performance of genotypes across different environments (Abebe Belete, 2019; Dlamini, 2016). In such cases, relying solely on the mean grain yield across environments may not provide a comprehensive or reliable measure of genotype performance (Yan, Kang, Ma, Woods, & Cornelius, 2007). GEI can be classified into two types: **non-crossover interaction**, where genotypes maintain their rank across environments with differences only in performance magnitude, and **crossover interaction,** where genotypes exhibit significant changes in rank between environments (Tesfaye, Bogale, Namara, & Bacha, 2008). The presence of GEI weakens the relationship between genotype and phenotype, leading to changes in relative rankings, stability inconsistencies, and reduced genetic progress in breeding programs (G. Kebede, Worku, Feyissa, & Jifar, 2023; Mwila, 2017).

Several statistical methods are available for analyzing multi-environment trials (METs), with Additive Main Effects and Multiplicative Interaction (AMMI) analysis being particularly effective for understanding genotype-by-environment interaction (GEI). AMMI combines analysis of variance and principal component analysis (PCA) to assess genotype stability through PCA scores and the AMMI Stability Value (ASV) (Achenef, 2022; Bocianowski et al., 2024). While AMMI’s additive component detects the significance of GEI, it does not pinpoint specific genotypes or environments contributing to the interaction. To address this limitation, GGE biplot analysis offers a comprehensive graphical tool for exploring METs data, visualizing genotype and environment performance, and ranking genotypes based on yield and stability (Hintsa and Fetien, 2013; Yan et al., 2007). Both AMMI and GGE biplot analyses are widely recognized for their effectiveness in evaluating GEI, facilitating the identification of high-yielding and stable varieties suited to diverse environmental conditions (Wodebo et al., 2023; El-Aty et al., 2024).

Several statistical methods are available for analyzing data from (METs), with Additive Main Effects and Multiplicative Interaction (AMMI) analysis being particularly effective for understanding genotype-by-environment interaction (GEI). AMMI combines analysis of variance and principal component analysis (PCA) to assess genotype stability through PCA scores and the AMMI Stability Value (ASV) (Achenef, 2022; Bocianowski, Nowosad, & Rejek, 2024). While AMMI’s additive component detects the significant of GEI, it does not pinpoint specific genotypes or environments contributing to the interaction. To address this limitation, the GGE biplot analysis offers a comprehensive graphical tool for fully exploring METs data, visualizing genotype and environment performance, and ranking genotypes based on yield and stability (Hintsa & Fetien, 2013; Yan *et al.*, 2007). Both AMMI and GGE analyses are widely recognized for their effectiveness in evaluating GEI, facilitating the identification of high-yielding and stable varieties suited to diverse environmental conditions (El-Aty *et al.*, 2024; Wodebo *et al.*, 2023). The main objectives of GGE biplots are to rank the genotypes, examine the environment, and graphically represent GEI interaction (Tekle, Tsehaye, Atsbeha, Abera, & Chiulele, 2024; Tulu & Wondimu, 2019).

Given the significant influence of environmental variability on genotype performance, stability assessment is crucial in plant breeding to identify genotypes with consistent yield across environments (Kaloki, Trethowan, & Tan, 2019). Stability measures, such as Shukla’s stability variance, which quantifies each genotype’s contribution to GEI with lower values indicating greater stability (Shukla, 1972), and the regression-based method of Finlay and Wilkinson (1963), which assesses genotype sensitivity and adaptability, have been foundational in this field. Genotypic stability is commonly categorized into two forms: static stability, where genotype performance remains relatively constant across environments, and dynamic stability, where performance changes in a consistent and predictable manner in response to environmental variation (Carvalho *et al*., 2024). These complementary concepts are critical for identifying genotypes that combine high productivity with reliable performance across diverse environmental conditions.

In Ethiopia, wheat covered approximately 1.87 million hectares, yielding approximately 5.8 million tons of grain during the 2021/22 cropping season (ESS, 2022). In the Amhara region, wheat cultivation spanned 689,614.06 hectares, yielding 1.92 million tons with productivity of 2.8 t ha⁻¹ (ESS, 2022). Overall, wheat is a staple crop in Ethiopia and is vital for food security and economic growth (Anteneh & Asrat, 2020).

However, its productivity is low in many regions, including the Amhara Region. This is because of different yield-limiting factors such as diseases, pests, climate variability, overuse of local varieties, lack of high-yielding and stable varieties, and poor communication between farmers and researchers. Nowadays, Ethiopia's regional and national agricultural research institutes have released several durum wheat varieties (Badebo, Gelalcha, Ammar, Nachit, & Abdalla, 2009; W. Tadesse *et al.*, 2022). Conversely, many of these varieties have not undergone comprehensive evaluation across the country, particularly in the northwestern regions (Zenebe, 2022). To fill this gap, it is essential to generate evidence on genotypic performance and adaptability under diverse environmental conditions. Such information is crucial for devising effective intervention strategies to enhance crop productivity and promote dietary self-sufficiency (Mekuriaw, 2023).Therefore, the current study evaluated and selected the best-adapted, high-yielding, and stable durum wheat varieties to improve crop yield across the Amahara Region, Northwestern Ethiopia.

**2. MATERIALS AND METHOD****S**

**2.1. features or descriptions of the Study area**

The study was conducted across three wheat-producing districts: Simada, Bichena, and Dabat, situated within the Aamara region, Ethiopia (Fig. 1 and Table 1). Specifically, it was conducted at the Farmers Training Centers (FTC) in each district during the main crop or rainy seasons between June and December 2022-2024 for two consecutive years. Soils in the tested location are Vertisols, with a pH of 5.5–7.0, moderate organic matter, and clayey texture. They contain moderate organic matter but face challenges such as phosphorus deficiency, erosion, and waterlogging in lowland areas. The locations were chosen purposively based on wheat production potential and soil type.

Table 1.Geographical description of the study area

| Location | Code  Year  2021/2022 | Code  Year  2022/2023 | Alt. (masl) | Temp. min & max values (°C) | RF.  Ave.(mm) | Soil type | Geographic location | |
| --- | --- | --- | --- | --- | --- | --- | --- | --- |
|  |  |  |  |  |  |  | Lat | Lon |
| Simada | E1 | E4 | 800-2220 | 18-27 | 1150 | Vertisol | 10 °22' N | 37° 47' E |
| Bichena | E2 | E5 | 2250 | 15-27 | 900-1200 | Vertisol | 10 38' N | 10° 37' E |
| Dabat | E3 | E6 | 2446 | 15-22 | 1380 | Vertisol | 10°21` N | 37° 43`E |

Where: Alt: Altitude, Temp: temperature, and RF: Rainfall, Lat: latitude, Lon: longitude

Source: District administrative office

Figure 1. Schematic map of the study area

**2.2. Experimental material and design**

Seeds of eight improved and high-yielding durum wheat varieties and one cheek varieties (Table 2), were obtained from the Kulmsa Agricultural Research Centers were evaluated under rain fed conditions in 2022 and 2023. and one variety was collected from the Adet Agricultural Research Center, Amhara, Ethiopia, and were sown on well-prepared plots. These nine varieties were tested across three environments (locations). Each experimental unit (plot) consisted of nine rows with 20 cm spacing between rows, resulting in a plot area of 3.6 m^2^ (1.8 m × 2 m). The field was plowed three times before sowing, and planting rows were prepared using a hand-pulled row marker. Seeds were manually planted using the hand drilling technique. A spacing of 0.5 m was maintained between plots, and 1 m was left between blocks. Since the two years (2022 and 2023) were also considered as blocks, the six combinations of the field blocks and years were used as blocks in the multilocation trial model. For each plot, a standard fertilizer application of 100 kg/ha NPS and 150 kg/ha urea was applied, following recommendations for durum wheat. Half of the urea and the full amount of NPS were applied during planting, while the remaining half of the urea was applied at the mid-tillering stage. A seed rate of 150 kg/ha was used. Weeding was carried out manually across all experimental fields at the three locations to manage weeds. No herbicides or fungicides were applied for weed or disease control.

Table 3. Variety used in the study

| **S. N** | **Variety code** | **Variety Name** | **Year of release** | **Center** |
| --- | --- | --- | --- | --- |
| 1 | G1 | Fetan | 2018 | DZARC |
| 2 | G2 | Don Matteo | 2018 | CGS Italian |
| 3 | G3 | Alemtena | 2015 | DZARC |
| 4 | G4 | Tesfaye | 2017 | DZARC |
| 5 | G5 | Wehabit | 2017 | Mekelle University |
| 6 | G6 | Rigeat | 2017 | Mekelle University |
| 7 | G7 | Utuba | 2015 | DZARC |
| 8 | G8 | Mangudo | 2012 | SARC/ORARI |
| 9 | G9 | Toltu | 2010 | SARC/ORARI |

**2.3. Data Collected**

The data for the eleven traits were collected on a plant-and-plot basis. Data were obtained on a plot basis based on all plants in the central rows of each plot, leaving two border row from each side of the plots. The ten representative plants per plot were randomly selected from the central rows for recording data on plant basis. Agronomic traits such as crop phenology, growth, yield, and yield components were assessed during the study period. Crop phenology included maturity day and days to heading, whereas growth parameters, such as plant height, number of productive tillers, number of grains per spike, number of spikelets per spike, spike length, and yield-related traits, including biomass yield, thousand seed weight, number of seed per spikelet, grain yield, and harvest index, were recorded.

**2.4. Data Analysis**

Data were analyzed using R statistical software version 4.4.1 (R Core Team, 2021). Analysis of variance (ANOVA) was conducted on the multi-environment data using the aov function from the **car** package (Fox & Weisberg, 2019) to evaluate the significance of genotype-by-environment interactions. The GGE model function from the **GGE Biplots** package was applied to the genotype-by-environment data matrix (Dumble, 2022). Subsequently, the "Which-Won" function was used to generate the "which-won-where" view of the GGE biplot. The **Env relationship** function in the same package was utilized to plot environmental relationships, the **Disc Rep** function was used to assess discrimination ability and representativeness, and the **Mean Stability** function was applied to create the discrimination ability and representativeness view and stability plots.

AMMI analysis was carried out using the AMMI function in the **agricolae** package (Mendiburu, 2017). Genotype stability was further assessed using additional stability parameters, including the coefficient of variation (CV) (Francis & Kannenberg, 1978), Shukla’s stability variance (Shukla, 1972), AMMI Stability Value (ASV) (Purchase et al., 2000), ecovalence (Ecoval) (Wricke, 1962), and deviation from joint regression analysis (Sij) (Eberhart & Russell, 1966). These parameters were estimated using the **metan** package in R (Olivoto & Lucio, 2020).

The ASV measures the stability of genotypes based on their interactions with the environment. Lower ASV values indicate more stable genotypes.

AMMI stability value was calculated as follows:

$$ASV= \surd[(SSIPCA1 / SSIPCA2) (IPCA1 score)]2 + (IPCA2 score)2$$

Where; SSIPCA1 and SSIPCA2 are the sums of the squares of the first and second principal component axes (IPCA), respectively. The IPCA 1 and IPCA2 scores are the interaction scores for the first and second IPCAs, respectively.

GGE biplot analysis is a graphical method that evaluates genotype performance and stability across environments by combining genotype and genotype-environment interaction effects, helping to identify the best and most adaptable genotypes.

**3. Results and Discussion**

**3.1. Combined analysis of Variance (ANOVA)**

The analysis of variance revealed highly significant differences (P < 0.01) among varieties for the traits considered in the experiment (Table 3). Locations also exhibited highly significant difference (p<0.01) for days to heading, tiller number, spike length, number of seeds per spikelets, plant height, thousand seed weight, biomass yield, grain yield, and harvest index. Additionally, varieties demonstrated highly significant variation (P < 0.01) across all evaluated traits. The year showed a highly significant effect (p<0.01) on plant height, spike length, biomass yield, grain yield, and thousand seed weight.

The interaction between variety and location (V x L) was highly significant (P < 0.01) for all traits tested in the durum wheat varieties. Similarly, the interaction between variety and year (V x Y) was highly significant (p < 0.01) for plant height, number of grains per spike, grain yield, and harvest index. The interaction between location and year (L x Y) was also highly significant (p < 0.01) for all tested traits except for the number of seeds per spikelet and biomass yield.

The three-way interaction effect of variety, location, and year (V x L x Y) was highly significant (p < 0.01) for days to heading, plant height, tiller number, spike length, number of seeds per spikelets, thousand seed weight, biomass yield, grain yield, and harvest index. The main effect of location by year (LxY) had highly significant (p < 0.01) effect on days to heading, maturity day, plant height, and grain yield. However, non significant effect was observed for the number of seeds per spikelet and biomass yield. The interaction effect of location by year had a highly significant (*P* < 0.01) effect on days to heading, maturity day, plant height, and grain yield, however, non significant effect was observed for the number of seeds per spikelet and biomass yield. As shown in Table 3, the interaction effect of location and variety had a significant (P < 0.05) effect on all evaluated traits. The interaction effect of variety by year was highly significant (P < 0.01) for plant height, number of seeds per spikelet, biomass yield, grain yield, and spike length. Conversely, a non-significant effect was observed for maturity date and days to heading.

Overall, the combined analysis revealed significant variations among the nine durum wheat varieties for most traits. These findings are consistent with previous studies by Delesa *et al.*, (2023), which reported significant differences (p < 0.05) among durum wheat genotypes across all individual test locations over multiple years.

Days to heading, plant height, tiller number, biomass yield, grain yield, and harvest index were all highly significant (p ≤ 0.01), as shown in Table 3. However, maturity day and the number of seeds per spike were not influenced by the interaction effects of year, variety, and location. In line with the present results, previous studies have reported highly significant differences (*p* ≤ 0.01) (Gebrie & Abebe, 2020). Birhanu, Tahir, Tilahun, Tilahun, & Hailu, (2020) also identified significant genotype-by- location interactions, with highly significant differences (p≤0.01) among food barely varieties for days to maturity and grain yield. The observed significant GEI interaction indicate inconsistent performance across the tested environments and highlight the varying discriminative abilities of these environments. This suggests the possibility to identify high-yielding and stable genotypes. A particular crop variety may perform well in one environment but poorly in another due to variations in climate, soil conditions, or management practices.

The results of this study are consistent with previous research by Dabi et al. (2024), which reported significant difference (P < 0.05) among bread wheat genotypes for grain yield at all individual test locations in multi-environment trials.

Table 4. Mean squares of the two years (cropping season) combined ANOVA results of Eleven quantitative traits from three locations

| **SOV** | Mean squares | | | | | | | | | |
| --- | --- | --- | --- | --- | --- | --- | --- | --- | --- | --- |
|  | **DH** | **DM** | **PH** | **TN** | **SL** | **NSPS** | **TSW** | **BY** | **GY** | **HI** |
| Genotypes (G) | 345.28** | 789.84** | 585.36** | 1.36** | 11.475** | 34.85** | 226.28** | 5.99** | 2.07** | 86.20** |
| Location (L) | 6.968^ns^ | 194.93^ns^ | 41.61** | 47.51** | 43.41** | 24.83** | 234.07** | 25.21** | 4.319** | 165.47** |
| Year (Y) | 0.964^ns^ | 89.92^ns^ | 164.36** | 0.03^ns^ | 0.30^ns^ | 2.45^ns^ | 27.16** | 3.43** | 4.200** | 1.05ns |
| V x L | 42.69* | 187.61* | 222.18** | 2.02** | 11.54** | 23.51** | 120.06** | 2.49** | 0.511** | 54.12** |
| V x Y | 39.36^ns^ | 95.88^ns^ | 37.53** | 0.59** | 1.29** | 7.30** | 22.20** | 0.42ns | 0.98** | 13.19ns |
| L x Y | 289.05** | 496.186* | 173.19** | 1.49** | 2.57** | 3.25^ns^ | 95.82** | 0.750ns | 2.003** | 59.46** |
| L x rep | 33.73^ns^ | 188.16^ns^ | 6.47^ns^ | 0.22^ns^ | 1.36** | 5.10^ns^ | 12.34** | 0.63ns | 0.037ns | 27.26ns |
| V x L x Y | 53.39** | 152.547^ns^ | 33.32** | 0.33** | 1.35** | 3.43^ns^ | 18.86** | 1.015** | 0.859** | 53.28** |
| Residuals | 22.59 | 103.58 | 10.45 | 0.12 | 0.33 | 2.74 | 2.53 | 0.41 | 0.02 | 14.35 |

Where SOV: source of variation; **: highly significant at *P* < 0.01; *: significant at *P* < 0.05; ns: non-significant; DF: Degrees of freedom; DH: days to heading; DM: days to maturity; PH: plant height; TN: tiller number; SL: Spike length; NSPS: number of spikelets per spike; BY: biomass yield; NSS: number of seed per spike, TSW: thawed seed weight, GY: grain yield; and HI: harvest index.

**3.2. Performance of the Genotype**

The grain yield performance of the durum wheat varieties showed significant variability across the tested locations. Tesfaye (G4) recorded the highest grain yield 3.74 t ha^-1^, indicating its superior adaptability and productivity under the test conditions. It was followed by Don Matteo (G2) which achieved a yield of 3.62 t ha^-1^, also demonstating strong performance. In contrast, Wehabit (G5) and Rigeat (G6) exhibited the lowest grain yields, at 2.75 t ha^-1^ and 2.78 t ha^-1^, respectively, suggesting their limited potential for high productivity in these environments.

The differences in grain yield among the genotypes can be attributed to variations in key yield components such as tiller number, spike length, and thousand seed weight. These findings underscore the importance of selecting high-yielding genotypes, such as Tesfaye (G4) to enhance durum wheat production in the study area.

The observed variability in grain yield reflects the influenced GEI interaction. High-yielding genotype, such as Tesfaye (G4) 3.74 t ha^-1^ demonstrate better adaptability and efficient resource utilization , whereas low-yielding genotype like Wehabit (G5) 2.75 t ha^-1^ show poor adaptability to the tested environments. This highlights the need to select, stable, high-performing genotype for sustainable wheat production.

Table 5. Mean grain yield and yield-related traits of durum wheat genotypes tested at three locations for two years

| Genotypes | DH | DM | PH | TN | SL | NSPS | TSW | BY | GY | HI |
| --- | --- | --- | --- | --- | --- | --- | --- | --- | --- | --- |
| 1 | 62.00^b^ | 104.44^bcd^ | 87.17^cd^ | 3.81^cd^ | 7.62^d^ | 19.43^a^ | 35.47^c^ | 10.47^b^ | 3.3^c^ | 26.74^e^ |
| 2 | 61.22^b^ | 110.72^ab^ | 98.01^a^ | 4.01^abc^ | 9.83^a^ | 19.39^a^ | 36.99^b^ | 11.08^a^ | 3.62^b^ | 30.81^abc^ |
| 3 | 53.44^c^ | 93.33^f^ | 88.29^c^ | 3.45^e^ | 7.69^d^ | 16.36^c^ | 34.46^cd^ | 9.64^c^ | 3.09^d^ | 31.25^ab^ |
| 4 | 70.72^a^ | 113.39^a^ | 94.23^b^ | 4.18^a^ | 9.06^b^ | 19.36^a^ | 41.23^a^ | 10.94^a^ | 3.74^a^ | 33.17^a^ |
| 5 | 62.11^b^ | 106.13^bc^ | 83.55^e^ | 4.11^ab^ | 8.47^c^ | 19.16^a^ | 31.44^f^ | 9.40^c^ | 2.75^f^ | 28.37^cde^ |
| 6 | 61.44^b^ | 101.78^cde^ | 78.53^f^ | 3.9^bc^ | 8.1^c^ | 16.64^bc^ | 31.12^f^ | 9.64^c^ | 2.78^f^ | 27.77^de^ |
| 7 | 63.39^b^ | 95.23^ef^ | 87.02^cd^ | 3.55^e^ | 7.47^d^ | 16.42^c^ | 29.5^g^ | 10.08^b^ | 2.95^e^ | 28.76^bcde^ |
| 8 | 62.89^b^ | 102.61^cd^ | 85.80^d^ | 4.19^a^ | 8.29^c^ | 17.66^b^ | 33.68^de^ | 10.15^b^ | 3.12^d^ | 29.65^bcd^ |
| 9 | 63.06^b^ | 98.97^def^ | 85.36^de^ | 3.66^de^ | 7.5^d^ | 16.89^bc^ | 32.63^e^ | 10.18^b^ | 3.15^d^ | 26.63^e^ |
| MSE | 22.59 | 103.58 | 10.45 | 0.12 | 0.33 | 2.74 | 2.53 | 0.41 | 0.02 | 14.35 |
| Mean | 62.25 | 102.96 | 87.55 | 3.87 | 8.22 | 17.92 | 34.06 | 10.18 | 3.17 | 29.24 |
| CV | 7.64 | 9.88 | 3.69 | 9.04 | 7.03 | 9.23 | 4.67 | 6.27 | 4.88 | 12.95 |
| LSD | 1.81 | 3.88 | 1.23 | 0.13 | 0.22 | 0.63 | 0.61 | 0.24 | 0.06 | 1.45 |

# 3.3. Genotype performance at each tested location.

The mean grain yield of the nine durum wheat genotypes across six environments (E1–E6) revealed notable GEI interaction, highlighting the differential performance of the genotypes under varying conditions (Table 5). **Tesfaye (G4)** consistently exhibited superior performance, achieving the highest mean grain yield (3.74 t ha^-1^), driven by its exceptional adaptability in environments E1 (3.97 t ha^-1^), E4 (3.97 t ha^-1^), and E6 (4.5 t ha^-1^). This indicates that Tesfaye is particularly well-suited to a range of environmental conditions. **Don Matteo (G2)** ranked second in overall performance with a grain yield of (3.62 t ha^-1^), excelling in E2 (4.7 t ha^-1^) and E4 (4.13 t ha^-1)^, suggesting its suitability for high-yielding environments, especially in Simada and Bichena. In contrast, **Wehabit (G5)** and **Rigeat (G6)** recorded the lowest mean grain yield, at 2.75 t ha^-1^ and 2.78 t ha^-1,^ respectively. Their consistently poor performance across most environments suggests limited adaptability or a higher sensitivity to environmental variations, making them less reliable for achieving high yields. Similarly, **Alemtena (G3),** despite excelling in E3 (4.82 t ha^-1^), had a lower overall mean yield (3.08 t ha^-1^) due to inconsistent performance in other environments, indicating that its high yield potential is not consistent across all conditions.

Environmental effects were evident, with **Dabat 2023 (E6) producing** the highest mean grain yield (3.767 t ha^-1^), followed by **Bichena 2022 (E1)** and **Bichena 2023 (E4), which recorded mean grain yielda of** 3.256 t ha ^-1^and 3.191 t ha^-1^, respectively. Conversely, **Simada 2022 (E2)** had the lowest mean yield (2.695 t ha^-1^), likely due to less favorable growing conditions in that region.

These results highlight the importance of selecting stable and high-yielding genotypes, such as **Tesfaye (G4)** and **Don Matteo (G2)**, for environments with diverse or challenging conditions. The variability in performance also underscores the critical role of GEI in influencing durum wheat yield and emphasizes the need for site-specific recommendations.

Table 6. Mean grain yield (t ha^-1^) of nine durum wheat genotypes across six environments (location and year combinations)

| Genotypes | (E1) | (E4) | (E3) | (E6) | (E2) | (E5) | Mean |
| --- | --- | --- | --- | --- | --- | --- | --- |
| **1** | 3.53b | 3.66b | 2.57d | 3.67b | 3.13a | 3.2b | 3.29 |
| **2** | 3.84a | 4.13a | 2.6d | 4.7a | 3.17a | 3.26ab | 3.62 |
| 3 | 2.74c | 2.91c | 4.82a | 2.82c | 2.41cd | 2.8c | 3.08 |
| **4** | 3.97a | 3.97a | 3.23b | 4.5a | 3.23a | 3.53a | 3.74 |
| 5 | 2.87c | 2.79cd | 2.75cd | 2.83c | 2.5bc | 2.77c | 2.75 |
| 6 | 2.7c | 2.63d | 2.92c | 3.63b | 2.29d | 2.53c | 2.78 |
| 7 | 2.85c | 2.85cd | 3.18b | 3.41b | 2.61b | 2.8c | 2.95 |
| 8 | 3.97a | 2.91c | 2.92c | 3.63b | 2.5bc | 2.77c | 3.12 |
| 9 | 2.85c | 2.85cd | 2.57d | 4.7a | 2.41cd | 3.53a | 3.15 |
| MSE | 0.03 | 0.023 | 0.015 | 0.031 | 0.007 | 0.03 |  |
| Mean | 3.256 | 3.191 | 3.062 | 3.767 | 2.695 | 3.02 | 3.16 |
| CV | 5.314 | 4.773 | 4.035 | 4.642 | 3.125 | 5.62 |  |
| LSD | 0.299 | 0.264 | 0.214 | 0.303 | 0.146 | 0.29 |  |

Where: E1=Bichena 2022, E2=Simada 2022,E3=Dabat 2022=E4=Bichena 2023=E5=Simada 2023, E6=Dabat 2023

**3.4. Additive Main Effects and Multiplicative Interaction (AMMI) Analysis**

Additive Main effect and Multiplicative Interaction (AMMI) analysis of variance for grain yield of the nine durum wheat genotypes evaluated across six environments is presented in (Table 6). The AMMI analysis revealed genetic variation and potential for selecting stable genotypes. The results demonstrated that the variation among E, G, and GEI was highly significant (*P* < 0.01). Sum squares (SS) partitioning revealed that the environmental effect was the largest contributor to variation followed by the GEI interaction and genotype main effect. Since changes in G and G × E are ordinarily smaller, the environment often explains most of the differences in genotype performance (Yan *et al.*, 2007).

Furthermore, the AMMI model's first two principal component axes (IPCAs) were found to be highly significant (*P* < 0.01) when used for GEI partitioning. The AMMI, which includes IPCA1 and IPCA2, is the best prediction model for cross-validating yield variation explained by the genotype-environment interaction (Mohammadi & Amri, 2012; Z. Tadesse, Habtemariam Zegeye, Tafesse Solomon, & Debele, 2018). Variations in the grain yield components from the AMMI indicated that genotype, environment, and genotype by environment interaction (GEI) all had significant (*P* < 0.01) effects, explaining variability in both environments and genotypes, and that there are possibilities for the selection of genotypes that are high-yielding, stable, and well-performing.

Grain yield was significantly impacted by environments, genotypes, and their interactions, according to AMMI analysis (P < 0.001). The sum of squares resulting from the GEI is further separated into five important Interaction Principal Components (PC-1 to PC-5) in consideration of the multiplicative component of AMMI (Table 6). The highest proportion of the total sum of squares was accounted for by the environment (29.78%), followed by the genotype by environment interaction (16.84%) and then genotype (16.6) (Table 6). According to Gauch (2006), this is consistent with the outcome anticipated from GEI analysis. The environment-related sum of squares' maximum magnitude indicates that the test conditions vary and have an impact on how the grain yield characteristic manifests itself. In line with findings from barley (Pour-Aboughadareh *et al.* 2022) and durum wheat grain yield (Mohammadi *et al*. 2015), the GEI and the largest percentage of environmental variance are in accord. The higher sum of squares due to the genotype-by-environment interaction compared to the genotype effect indicates the presence of mega-environments (**Fig. 2**). The findings reveal that the various genotypes reacted differently to the environmental conditions, highlighting the phenotypic diversity among the evaluated genotypes. This also implies the potential presence of distinct mega-environments, each with genotypes that exhibit varying performance levels.

Table 7. AMMI analysis of variance for grain yield of nine durum wheat genotypes

| Source | DF | Sum square | Mean square | Proportion | Accumulated |
| --- | --- | --- | --- | --- | --- |
| ENV | 5 | 29.78 | 3.36** | 45.22% | NA |
| REP(ENV) | 12 | 0.49 | 0.04** | 0.74% | NA |
| GEN | 9 | 16.6 | 2.08** | 25.2% | NA |
| GEN: ENV | 40 | 16.84 | 0.75** | 25.86% | NA |
| PC1 | 12 | 20.72 | 1.72** | 69.6 | 69.6 |
| PC2 | 10 | 5.62 | 0.56** | 18.9 | 88.5 |
| PC2 | 8 | 2.11 | 0.26** | 7.1 | 95.6 |
| PC4 | 6 | 1.11 | 0.19** | 3.7 | 99.3 |
| PC5 | 4 | 0.22 | 0.06** | 0.7 | 100 |
| Residuals | 96 | 2.15 | 0.02 | NA | NA |
| Total | 201 | 95.66 | 0.47 | NA | NA |

Figure 2. AMMI Analysis model biplot for grain yield of durum wheat genotypes evaluated at six environments. Where: G1: Fetan, G2: Don Matteo, G3: Alemtena, G4: Tesfaye, G5: Wehabit, G6: Rigeat, G7: Utuba, G8:Mangudo, and G9: Toltu genotype (E1: Simada, E2: Bichena, E3: Dabat 2022 cropping season and E4: Simada, E5: Bichena, E6: Dabat in 2023 cropping season. The blue numbers connected to the polygon represent the genotypes, whereas the green numbers represent the locations/test environments.

**3.5. Stability Analysis and Mega-Environment Classification Using GGE Biplot.**

**3.5.1. GEI analysis for which-won-where patterns using GGE biplot**

The GGE results were similar to those obtained from AMMI and combined ANOVA. The polygon view of the GGE biplot highlights the top-performing genotype in each environment, visually representing key concepts such as crossover interaction, mega-environment differentiation, and specific adaptation (Yan and Tinker, 2005). Mega environment analysis refers to dividing a crop-growing region into distinct target zones (Gauch and Zobel, 1997).

In this analysis, six vertex genotypes were identified: G3 (Alemtena), G4 (Tesfaye), G2 (Don Matteo), G5 ( Wehabit), G6 ( Rigeat), and G9 (Toltu). The biplot was divided into six sections by using six rays, with environments distributed across three of these sections while genotypes appeared in all. Each sector's vertex genotype exhibited the highest grain yield for the environments within that sector, indicating specific adaptation to particular mega-environments.

The GGE biplot analysis identified three distinct wheat-growing mega-environments. The first mega-environments comprised environments E1 (Bichena 2022 ), E2 (Simada 2022 ), and E4 (Bichena 2023), with G4 (Tesfaye ) identified as the winning genotype. The second mega environment E5(Simada 2023), and E6 (Dabat 2023 ),where G9 (Toltu) was the top performimg genotype., Conversely, E3 (Dabat 2022) belonged to the third mega-environment with G3 (Alemtena) as its vertex genotype. Notably, no mega-environments were associated with sectors where genotypes G6 (Rigeat), G7(Utuba ), and G8 (Mangudo) were vertex genotypes, indicating their unsuitability for any of the tested environments. Previous studies by (Scavo, Mauromicale, & Ierna, 2022) have reported similar findings, suggesting that an ideal genotype should have high mean performance and stability. The genotypes that performed the best or worst in specific environments were the furthest from the biplot's origin ( Bekana, 2023), which were more sensitive to environmental changes, and regarded as specifically adapted genotypes. Select the best environments within their respective sectors in the GGE-biplot's polygon view (Khare, Shukla, Pandey, Singh, & Singh, 2024). Therefore, G4 (Tesfaye) had the highest yield in Bichena and Simada 2022, G9 (Toltu) had the highest yield in Simada and Dabat, and G3(Alemtena) had the highest yield in Dabat 2022. The most effective method for identifying superior genotypes and visualizing the patterns of interaction between genotypes and environments in data analysis is the polygon view of the GGE biplot, which helps to estimate the potential existence of various mega-environments (Demelash, 2024; Wardofa, Asnake, & Mohammed, 2019). Vertex genotype G4 falls in the first and second quadrants, G9 falls in the third quadrant whereas G3 is located in the second and fourth quadrants. Therefore, the six testing environments were categorized into three mega environments, while the nine genotypes were divided into six genotypic groups (**Fig. 3**).

Figure 3 Which-won-where view of GGE biplot for grain yield of durum wheat genotype

Where: G1: Fetan, G2: Don Matteo, G3: Alemtena, G4: Tesfaye, G5: Wehabit, G6: Rigeat, G7: Utuba, G8:Mangudo, and G9: Toltu genotype (E1: Simada, E2: Bichena, E3: Dabat 2022 cropping season and E4: Simada, E5: Bichena, E6: Dabat in 2023 cropping season. The blue numbers connected to the polygon represent the genotypes, whereas the green numbers represent the locations/test environments.

**3.5.2. Evaluation of environments relative to the ideal environment**

Through the representation of the center of the concentric circles in a polygon perspective, the discrimination and representativeness of the GGE biplot often illustrate the ranking of an ideal test environment for a particular genotype or for all genotypes (Fig. 4). A point located on the average environment axis in the positive direction,with the longest vector from the biplot origin is considered the most representative and thus the optimal test environment in the GGE biplot (Yan and Tinker, 2006). The test environment that is both most representational of the target environment and most discriminating (informative) is deemed optimal. The ideal test environment is represented by the center of the concentric circles (Fig. 4). E5 and E2 performed the worst in selecting genotypes adapted to the entire region, whereas E1, E4, and E6 were closest to the ideal environment point and, therefore, best represented the Durum Wheat production environments.

Figure 4. Discrimination vs. representativeness of genotypes. Where: E1: Simada, E2: Bichena, E3: Dabat 2022 cropping season and E4: Simada, E5: Bichena, E6: Dabat in 2023 cropping season.

**3.5.3. Evaluation of genotypes relative to the ideal genotype**

The ideal genotype for durum wheat production is positioned at the center of the concentric circles, in the GGE biplot symbolizing optimal traits characterized by high mean yield and stability. In this context, Mangudo (G8) stands out desirable genotype due to its exceptional yield performance and stable characteristics, making it highly suitable for grain production. Genotypes that are close to the ideal genotype, such as Fetan (G1), Utuba (G7), and Wehabit (G5), are also recognized for their favorable performance and stability, aligning closely with the traits of the optimal genotype. These genotypes are therefore considered strong candidates for cultivation.

In contrast, genotypes positioned further from the ideal center specifically Don Matteo (G2), Toltu (G9), and Alemtena (G3) are considered less desirable due to their low yield performance **(fig.5)**. This classification is supported by data from the mean separation test referenced in fig 5, which corroborates these observations regarding yield stability and performance. Overall, the analysis highlights the value of selecting genotypes based on their proximity to the ideal genotype as depicted in the concentric circle model, of GGE biplot. This approach effectively illustrates the relationship between yield performance and genetic stability in durum wheat breeding programs. Supporting this finding, Mulugeta *et al.* (2022) identified variety G-169 as the most ideal genotype for durum wheat grain production. Additionally, Multivariate analyses of Ethiopian durum wheat revealed genotypes that are both high-yielding and stable across trials. The relative contributions of stability and grain yield for identifying desirable genotypes found in this study, using the ideal genotype procedure of the GGE biplot, were also similar to those reported by Adem Wado (2020) genotype × location × planting pattern interaction and stability analysis of sorghum (Sorghum bicolor (L.) Moench) varieties in the drylands of Ethiopia.

Figure 5.Ranking of genotypes based on yield performance and stability. Where: G1: Fetan, G2: Don Matteo, G3: Alemtena, G4: Tesfaye, G5: Wehabit, G6: Rigeat, G7: Utuba, G8:Mangudo, and G9: Toltu genotype (E1: Simada, E2: Bichena, E3: Dabat 2022 cropping season and E4: Simada, E5: Bichena, E6: Dabat in 2023 cropping season. The blue numbers connected to the polygon represent the genotypes, whereas the green numbers represent the locations/test environments.

**3.5.4. Interrelationship among environment**

In a GGE biplot, the correlation intensity between enviroment is represented by the cosine of the angle between any two environment vectors (Yan and Kang, 2003). A correlation is considered positive if the angle is less than 90°, negative if it is greater than 90°, and nonexistent if it is around 90°. According to Yan (2002) and Yan and Kang (2003), there is a significant connection between the correlation coefficient of two environments and the angle between their vectors, which is utilized to categorize the test environments. Therefore, the top-yielding genotypes in one environment will perform best in other environments if two environments have a positive correlation. In the context of GGE biplot analysis,understanding the relationship between environments is crucial for interpreting genotype performance across different conditions. When two environments show a negative correlation, it suggests that genotypes performing well in one are likely to perform poorly in the other, and vice versa (Yan *et al.*, 2007; Yan & Tinker, 2006). In the present study, as shown in (**Fig 6**), the environments Simada, and Bichena are positively correlated, as indicated by an angle their vectors less than 90°. On the other hand, the angle between Dabat and simada greater than 90°, indicating a negative correlationbetween these two environments.

Figure 6.Ranking of environments relative to the ideal environments

Where: G1: Fetan, G2: Don Matteo, G3: Alemtena, G4: Tesfaye, G5: Wehabit, G6: Rigeat, G7: Utuba, G8:Mangudo, and G9: Toltu genotype (E1: Simada, E2: Bichena, E3: Dabat 2022 cropping season and E4: Simada, E5: Bichena, E6: Dabat in 2023 cropping season. The blue numbers connected to the polygon represent the genotypes, whereas the green numbers represent the locations/test environments.

**3.6. Stability of durum wheat genotypes**

Genotypes' mean grain yield and stability performance are graphically represented using the average environment coordination (AEC) method (**Fig.7**). Combining grain yield with genotype stability performance can help identify the genotypes that yield the highest and most stable results (Mullualem *et al.*, 2024). The genotype with the highest mean performance and greatest stability across all test environments is considered ideal (Vaezi *et al.*, 2019; Wagaw *et al.*, 2021). According to the AEC view in the comparison biplot, a desirable genotype are those located close to the ideal genotype, which is typically positioned at the center of the concentric circles or at the tip of the arrow indicating the ideal point. In the ACE system, the mean performance of genotypes is represented by an arrow along the AEC X-axis (PC1), which passes through intersects the origin of the biplot and indicates the Average Environmental. This graphical representation is instrumental in visually distinguishing high-performing, stable genotypes from those with variable or suboptimal performance.

On the positive side of the AEC abscissa, genotypes located closer to the origin would have a higher mean grain yield. In contrast, genotypes farther away on the negative side would have a lower mean grain yield. Additionally, a genotype's projection becomes less stable the farther it is from the origin in absolute terms. This line indicates a higher mean yield across the environments. Hence, in the present study, G2 gave the highest mean yield, followed by G1, and G6. The performance of the remaining genotype groups (G3 and G5) was below the grand mean yield (**Fig. 7**). An ideal genotype is completely stable across a wide range of environments and has the highest average performance of any genotype (Bekana, 2023).

Higher GEI interaction and lower stability are indicated by genotype that lie further from the origin along the AEC ordinate (y-axis), in either direction (Kaya, Akçura, & Taner, 2006). Based on this criterion, G6, G4, and G1 which have the shortest vector from the AEC axis, were identified as the most stable genotypes. In contrast, G2 and G3 with the longest vector from AEC axis, were classified as unstable genotypes.However, G2, followed by G1, scored higher in grain yield, whereas G5 and G3 recorded the lowest grain yield across all environments (**Fig.7**).

An ideal genotype for a specific environment has the highest mean yield and responds best in that particular environment while being less stable in the other environments, thus necessitating recommendations for the specific environment (Abraha, Shimelis, Solomon, & Hailu, 2019). Stability is reported to have lower heritability than mean performance. It is important to note that stability pertains to the genotype's relative performance and gains significance only when linked to the mean (Atsbeha, Mekonnen, Kebede, Haileselassie, & Tesfaye, 2023). In summary, environmental factors (e.g., temperature, rainfall, soil quality) and genetic factors (e.g., drought resistance, nutrient efficiency) jointly influence yield-related parameters such as biomass, spike length, and the number of tillers. Wheat varieties with favorable genetic factors tend to perform better under various environmental conditions, resulting in improved overall yield (Alemu *et al.*, 2021; Eskezia *et al.*, 2024; Gebrewahid, Mengistu, Tsehaye, Aberha, & Aberra, 2021). Genotype stability was further evaluated using different stability parameters.

Figure 7.Which genotype was more stable across the tested environments? Where: G1: Fetan, G2: Don Matteo, G3: Alemtena, G4: Tesfaye, G5: Wehabit, G6: Rigeat, G7: Utuba, G8:Mangudo, and G9: Toltu genotype (E1: Simada, E2: Bichena, E3: Dabat 2022 cropping season and E4: Simada, E5: Bichena, E6: Dabat in 2023 cropping season. The blue numbers connected to the polygon represent the genotypes, whereas the green numbers represent the locations/test environments.

**3.7. Stability parameter**

**3.7.1. The regression coefficient**

The performance of a genotype in an environment is determined by its mean performance, environmental responsiveness as a linear function, and its deviation from regression. Eberhart and Russell suggested using a linear regression coefficient and the variance of the regression deviations to evaluate crop responses to environmental changes. The average stability is indicated by a regression coefficient (bi) that approaches one and a deviation from regression (S2di) of zero. In this model, regression scores above one indicate varieties that are more sensitive to changes in the environment (below-average stability) and are specifically adaptable to high-yielding environments (Mansour, Moustafa, El-Naggar, Abdelsalam, & Igartua, 2018). Conversely, regression coefficients below one enhance the specificity of adaptability to low-yielding environments by demonstrating greater resistance to environmental change (above-average stability).

The present results indicated that linear regression of the average grain yield of individual genotype on the average yield of all varieties in each environment produced regression coefficients (bi values) ranging from -0.012 to 1.73. This variation in regression coefficients reflects differing responses of the varieties to environmental changes (Table 7). A mean regression coefficient (bi) that is close to one, low deviation form regression, and grain yields higher than the grand mean indicate that the aforementioned varieties Fetan, Tesfaye and Utuba are well adapted to all environments, indicating broader adaptation across different environments. In contrast Toltu and Mangudo are poorly adapted to all environments. It is recommended that these varieties be cultivated under unfavorable conditions, as they demonstrate resilience to environmental variations. Similar finding have been reported by other researchers (Delesa *et al.*, 2022).

Table 8. The various models of stability used to partition the GEI interaction for grain yield in the test of durum wheat genotypes and their ranking

| Genotypes | Mean | Bi | s^2^di | σ^2^ | wi | pi | s1 | s2 | ASI | ASV |
| --- | --- | --- | --- | --- | --- | --- | --- | --- | --- | --- |
| 1 | 3.3 | 0.84 | 0.14 | 0.12 | 97.70 | 0.11 | 0.27 | 7.77 | 0.28 | 1.38 |
| 2 | 3.51 | 1.41 | 0.21 | 0.22 | 102.00 | 0.00 | 0.33 | 10.70 | 0.40 | 2.00 |
| 3 | 3.02 | -0.012 | 0.62 | 0.74 | 80.30 | 0.80 | 0.20 | 8.67 | 0.76 | 3.78 |
| 4 | 3.69 | 0.98 | 0.04 | 0.03 | 114.00 | 0.00 | 0.07 | 1.47 | 0.18 | 0.87 |
| 5 | 2.75 | 0.35 | 0.00 | 0.03 | 84.70 | 0.79 | 0.40 | 4.97 | 0.11 | 0.54 |
| 6 | 2.78 | 1.32 | 0.06 | 0.05 | 83.30 | 0.66 | 0.33 | 4.57 | 0.11 | 0.56 |
| 7 | 2.95 | 0.73 | 0.04 | 0.03 | 90.00 | 0.56 | 0.13 | 3.50 | 0.17 | 0.87 |
| 8 | 3.12 | 1.43 | 0.14 | 0.20 | 91.30 | 0.27 | 0.27 | 8.80 | 0.10 | 0.48 |
| 9 | 3.07 | 1.73 | 0.22 | 0.27 | 87.40 | 0.45 | 0.00 | 14.60 | 0.27 | 1.33 |

**S^1^**= Nassar and Huens’s (1987) Mean of the absolute rank differences of a genotype over the n environments, **S^2^**= Nassar and Huens’s (1987) variance among the ranks over the k environments, **Wi**=Wriecke’s ecovalence, **Pi** =Cultivar superiority, **σ^2^** = Shukla's (1972) stability variance, **bi** =Eberhart and Russell (1966) stability value of regression coefficient and **S^2^di**= Eberhart and Russell (1966) stability deviation value from regression, ASI= AMMI stability index, and ASV=AMMI stability value.

**3.8. Limitation of the Study**

While the findings provide valuable insights into the performance and stability of durum wheat genotypes, it is important to acknowledge certain limitations. The study is based on data collected over only two years and across six environments, which may not fully capture the extensive environmental variability affecting durum wheat production. Additionally, although the statistical methods used are robust, they carry inherent assumptions and limitations that could influence the interpretation of genotype-by-environment interactions. Therefore, recommendations for commercial cultivation should be made with caution. Conducting further multi-year and multi-location trials would enhance the reliability and generalizability of these results.

**4. Conclusions and Recommendation**

Analysis of GEI interaction in this study highlighted the importance of evaluating durum wheat genotypes across multiple environments to determine their yield stability and adaptability. The combined analysis of variance over the years showed significant difference among genotypes, environments, and their interaction, confrining that both genetic and environmental factors significantly influenced grain yield and other agronomic traits. Genotype Tesfaye, followed by Don Matteo, consistently exhibited superior phenotypic performance across all traits,with grain yields of 3.74 and 3.62 tha^−1^, respectively, making them the top-yielding genotypes. Grain yield across test environments varied substantially, ranging from 4.5 t ha⁻¹ in Dabat to 2.29 t ha⁻¹ in Simada.The first principal component (PC1) contributed significantly to the interaction, accounting for 69.76%, whereas the second principal component (PC2) explained an additional 21.71%, together accounting for 91.47% of the genotype-by-environment interaction. AMMI and GGE biplot analyses effectively visualized the GEI patterns, grouping the six test environments and nine genotypes into three mega-environments and six genotype clusters. Simada and Dabat emerged as both highly discriminating and representative environments, making them ideal sites for evaluating genotype performance. Tesfaye*,* Fetan*, and* Rigeat were identified as the most stable and high-yielding genotypes, while Wehabit recorded the lowest mean yield (2.75 t ha⁻¹).

Tesfaye showed specific adaptability to Bichena and Simada, Toltu to Simada and Dabat, and Alemtena to Dabat. Based on these results, it is recommended to cultivate Tesfaye and Don Matteo in the Amhara region and other similar durum wheat-growing areas, given their high yield and moderate to high stability. Additionally, Simada and Dabat should be prioritized as core testing sites in future multi-environment trials due to their strong discriminative and representative capacities. Continued exploration of GEI is essential to refine varietal recommendations and ensure optimal wheat production in specific agro-ecological zones.

**Conflict of interests**

The authors have not declared any conflict of interest.

**Authors’ contribution statement**

**A.E:** Wrote the original draft, data collected and analyzed, investigated, revised, and edited; **A. A** : Restructured, reviewed, collected the data, and analyzed; **Y.A**: Contributed to reviewing, data collection, and editing, **M.A:** Restructured, Contributed to reviewing, data collection and editing and **K.N:** Restructured, Contributed to reviewing, data collection and editing All authors read and approved the final version of the manuscript.

**Ethics approval and consent to participate**

Not applicable

**Consent for publication**

Not applicable

**Availability of data and material**

Datasets generated in this study are available from the corresponding author upon reasonable request.

**Funding statement:**

No found source

**5. REFERENCES**

Abdalla, Ahmed, Stellmacher, Till, & Becker, Mathias. (2022). Trends and prospects of change in wheat self-sufficiency in Egypt. *Agriculture, 13*(1), 7.

Abebe Belete, Desta. (2019). *GENOTYPE X ENVIRONMENT INTERACTION AND STABILITY ANALYSIS IN UPLAND RICE (Oryza sativa L.) VARIETIES IN ETHIOPIA.* Haramaya university.

Abraha, Mizan Tesfay, Shimelis, Hussien, Solomon, Teka, & Hailu, Azeb. (2019). Genotype-by-environment interaction and selection of elite wheat genotypes under variable rainfall conditions in northern Ethiopia. *Journal of Crop Improvement, 33*(6), 797-813.

Abtew, Wosene G, Lakew, Berhane, Haussmann, Bettina IG, & Schmid, Karl J. (2015). Ethiopian barley landraces show higher yield stability and comparable yield to improved varieties in multi-environment field trials. *Journal of plant breeding and crop science, 7*(8), 275-291.

Achenef, Gebeyaw. (2022). Advancement of Analytical Models quantifying G× E Interactions and Stability analysis in Multi-Environment Trial.

Adem Wado, Dima. (2020). *GENOTYPE X LOCATION X PLANTING PATTERN INTERACTION AND STABILITY ANALYSIS OF SORGHUM [Sorghum bicolor (L.) Moench] VARIETIES IN DRYLANDS OF ETHIOPIA.* Haramaya university.

Alemu, Gadisa, Dabi, Alemu, Geleta, Nagesh, Duga, Ruth, Solomon, Tafesse, Zegaye, Habtemariam, . . . Asefa, Bayisa. (2021). Genotype× environment interaction and selection of high yielding wheat genotypes for different wheat-growing areas of Ethiopia. *Am. J. Biosci, 9*, 63-71.

Anteneh, Adugnaw, & Asrat, Dagninet. (2020). Wheat production and marketing in Ethiopia: Review study. *Cogent Food & Agriculture, 6*(1), 1778893.

Atsbeha, Genet, Mekonnen, Tilahun, Kebede, Mulugeta, Haileselassie, Teklehaimanot, & Tesfaye, Kassahun. (2023). Multivariate analyses, heritability and genotype environment interaction of bread wheat genotypes in Ethiopia. *Ecological Genetics and Genomics, 29*, 100209.

Badebo, Ayele, Gelalcha, Solomon, Ammar, K, Nachit, MM, & Abdalla, O. (2009). Overview of durum wheat research in Ethiopia: challenges and prospects.

Baranski, Marci. (2015). *The wide adaptation of green revolution wheat*: Arizona State University.

Bekana, Getahun. (2023). Genotype X Environment Interactions and Yield Stability of Tef (Eragrostis tef) Genotypes Grown in Central Parts of Ethiopia. *American Journal of BioScience, 11*(4), 104-110.

Beres, Brian L, Rahmani, Elham, Clarke, John M, Grassini, Patricio, Pozniak, Curtis J, Geddes, Charles M, . . . Ransom, Joel K. (2020). A systematic review of durum wheat: Enhancing production systems by exploring genotype, environment, and management (G× E× M) synergies. *Frontiers in Plant Science, 11*, 568657.

Birhanu, Asaye, Tahir, Zeynu, Tilahun, Manye, Tilahun, Mellie, & Hailu, Abebe. (2020). Performance Evaluation and Adaptation of Food Barley (Hordeum Vulgare L.) Varieties in the Highlands of North Gondar Ethiopia. *American Journal of Agriculture and Forestry, 8*(6), 252-257.

Bocianowski, Jan, Nowosad, Kamila, & Rejek, Dariusz. (2024). Genotype-environment interaction for grain yield in maize (Zea mays L.) using the additive main effects and multiplicative interaction (AMMI) model. *Journal of Applied Genetics*, 1-12.

Cappelli, Alessio, & Cini, Enrico. (2021). Challenges and opportunities in wheat flour, pasta, bread, and bakery product production chains: A systematic review of innovations and improvement strategies to increase sustainability, productivity, and product quality. *Sustainability, 13*(5), 2608.

Delesa, Abebe, Alemu, Gadisa, Geleta, Negash, Dabi, Alemu, Zegeye, Habtemariyam, Solomon, Tafesse, . . . Asefa, Bayisa. (2022). Stability and performance evaluation of advanced bread wheat (Triticum aestivum L.) genotypes in optimum areas of Ethiopia. *International Journal of Bio-resource and Stress Management, 13*(Jan, 1), 69-80.

Demelash, Habtamu. (2024). Genotype by environment interaction, AMMI, GGE biplot, and mega environment analysis of elite Sorghum bicolor (L.) Moench genotypes in humid lowland areas of Ethiopia. *Heliyon, 10*(5).

Dibaba, Regasa. (2019). Wheat production, marketing and consumption in Ethiopia. *Journal of Marketing and Consumer Research, 55*, 10-19.

Dlamini, Njabulo Eugene. (2016). *Genotype by environment interaction and resource optimization in sugarcane variety evaluation in Swaziland.* University of the Free State.

El-Aty, Mohamed S Abd, Abo-Youssef, Mahmoud I, Sorour, Fouad A, Salem, Mahmoud, Gomma, Mohamed A, Ibrahim, Omar M, . . . El-Tahan, Amira M. (2024). Performance and stability for grain yield and its components of some rice cultivars under various environments. *Agronomy, 14*(9), 2137.

Eskezia, Alemnesh, , Habtamu Kefale, & , Mekonen Asrat. (2024). Grain yield Performance Evaluation of Bread Wheat (Triticum aestivum L.) Varieties in

East Gojjam Zone. *Advanced Journal of Interdisciplinary Studies, 8(2)*, 1663-1676.

ESS. (2022). Ethiopian Statistical Services). 2022. Report on Area and Production of Major Crops (private peasant holdings, Meher season). Volume I, Statistical Bulletin 593, Addis Ababa, Ethiopia.

Gebrewahid, Lijalem, Mengistu, Dejene K, Tsehaye, Yemane, Aberha, Addis, & Aberra, Dereje A. (2021). Variability among Ethiopian durum wheat genotypes grown under different climatic conditions of Tigray for some agronomic and grain-quality traits. *Journal of Crop Improvement, 35*(2), 184-203.

Gebrie, Gedifew, & Abebe, Desta. (2020). Adaptation and performance evaluation of bread wheat (Triticumaestivum L.) varieties in Guagusa-shikudad District of North western Ethiopia. *Asian J Plant Sci Res, 10*(6), 64-68.

Hailu, Faris. (2017). Farmers perception of pesticide use and genetic erosion of landraces of tetraploid wheat (Triticum spp.) in Ethiopia. *Genetic resources and crop evolution, 64*, 979-994.

Hassani, Mahdi, Mahmoudi, Seyed Bagher, Saremirad, Ali, & Taleghani, Dariush. (2023). Genotype by environment and genotype by yield* trait interactions in sugar beet: analyzing yield stability and determining key traits association. *Scientific Reports, 13*(1), 23111.

Hintsa, G Hagos, & Fetien, A. (2013). AMMI and GGE biplot analysis of bread wheat genotypes in the Northern part of Ethiopia. *Journal of Plant Breeding and Genetics, 1*, 12-18.

Hodson, DP, Jaleta, Moti, Tesfaye, Kindie, Yirga, Chilot, Beyene, Habtamu, Kilian, A, . . . Daba, T. (2020). Ethiopia’s transforming wheat landscape: tracking variety use through DNA fingerprinting. *Scientific Reports, 10*(1), 18532.

Kaloki, Peter, Trethowan, Richard, & Tan, Daniel KY. (2019). Effect of genotype× environment× management interactions on chickpea phenotypic stability. *Crop and Pasture Science, 70*(5), 453-462.

Kaya, Yüksel, Akçura, Mevlüt, & Taner, Seyfi. (2006). GGE-biplot analysis of multi-environment yield trials in bread wheat. *Turkish journal of agriculture and forestry, 30*(5), 325-337.

Kebede, Gezahagn, Worku, Walelign, Feyissa, Fekede, & Jifar, Habte. (2023). Genotype by environment interaction and stability analysis for selection of superior fodder yield performing oat (Avena sativa L.) genotypes using GGE biplot in Ethiopia. *Ecological Genetics and Genomics, 28*, 100192.

Kebede, Metiku, Temam, Bilal, Getahun, Muluken, & Tsegaye, Yisahake. (2020). Evaluation of durum wheat (Triticum turgidum) varieties against stem rust (Puccinia graminis f. sp. tritici) in selected districts of southern Ethiopia.

Khare, Vikrant, Shukla, Rama Shankar, Pandey, Suneeta, Singh, Sanjay Kumar, & Singh, Charan. (2024). Exploring the genotype-environment interaction of bread wheat in ambient and high-temperature planting conditions: a rigorous investigation. *Scientific Reports, 14*(1), 2402.

Mansour, Elsayed, Moustafa, Ehab SA, El-Naggar, Nehal ZA, Abdelsalam, Asmaa, & Igartua, Ernesto. (2018). Grain yield stability of high-yielding barley genotypes under Egyptian conditions for enhancing resilience to climate change. *Crop and Pasture Science, 69*(7), 681-690.

Mekuriaw, Abate. (2023). Agroecology-specific varietal matching to achieve self-sufficiency in wheat production in Ethiopia. *Heliyon, 9*(3).

Minot, Nicholas, Warner, James, Lemma, Solomon, Kasa, Leulsegged, Gashaw, Abate, & Rashid, Shahidur. (2019). The wheat supply chain in Ethiopia: Patterns, trends, and policy options. *Gates Open Res, 3*(174), 174.

Mohammadi, Reza, & Amri, Ahmed. (2012). Analysis of genotype× environment interaction in rain-fed durum wheat of Iran using GGE-biplot and non-parametric methods. *Canadian Journal of Plant Science, 92*(4), 757-770.

Mullualem, D., Tsega, A., Mengie, T., Fentie, D., Kassa, Z., Fassil, A., . . . Astatkie, T. (2024). Genotype-by-environment interaction and stability analysis of grain yield of bread wheat (Triticum aestivum L.) genotypes using AMMI and GGE biplot analyses. *Heliyon, 10*(12), e32918. doi: 10.1016/j.heliyon.2024.e32918

Mulugeta, Behailu, Tesfaye, Kassahun, Geleta, Mulatu, Johansson, Eva, Hailesilassie, Teklehaimanot, Hammenhag, Cecilia, . . . Ortiz, Rodomiro. (2022). Multivariate analyses of Ethiopian durum wheat revealed stable and high yielding genotypes. *PloS One, 17*(8), e0273008.

Mwila, Chibanda. (2017). *Grain yield stability, genetic gain and path coefficient analyses in advanced soybean (Glycine max (L.) Merr.) lines.*

Paul, Keryn I, Roxburgh, Stephen H, Chave, Jerome, England, Jacqueline R, Zerihun, Ayalsew, Specht, Alison, . . . Adams, Mark A. (2016). Testing the generality of above‐ground biomass allometry across plant functional types at the continent scale. *Global change biology, 22*(6), 2106-2124.

Scavo, Aurelio, Mauromicale, Giovanni, & Ierna, Anita. (2022). Dissecting the genotype× environment interaction for potato tuber yield and components. *Agronomy, 13*(1), 101.

Shiferaw, Bekele, Smale, Melinda, Braun, Hans-Joachim, Duveiller, Etienne, Reynolds, Mathew, & Muricho, Geoffrey. (2013). Crops that feed the world 10. Past successes and future challenges to the role played by wheat in global food security. *Food security, 5*, 291-317.

Tadesse, Wuletaw, Zegeye, Habte, Debele, Tolesa, Kassa, Daniel, Shiferaw, Wondwosen, Solomon, Tafesse, . . . Assefa, Solomon. (2022). Wheat production and breeding in Ethiopia: retrospect and prospects. *Crop Breeding, Genetics and Genomics, 4*(3).

Tadesse, Zerihun, Habtemariam Zegeye, Dawit Asnake, Tafesse Solomon, Yewubdar Shewaye, & Debele, Tolessa. (2018). Identification of Stable Bread Wheat (Triticum Aestivum L.) Genotypes using AMMI Analysis in Ethiopia. *Int. J. Res. Agric. For, 5*, 6-14.

Tekle, Hailekiros Tadesse, Tsehaye, Yemane, Atsbeha, Genet, Abera, Fetien Abay, & Chiulele, Rogério Marcos. (2024). Investigation of genotype x environment interaction for Hordeum vulgare L. ssp. vulgare recombinant inbred lines in multi-environments of Tigray, Ethiopia. *Ecological Genetics and Genomics, 31*, 100231.

Tesfaye, Abonesh, Bogale, Ayalneh, Namara, Regassa E, & Bacha, Dereje. (2008). The impact of small-scale irrigation on household food security: The case of Filtino and Godino irrigation schemes in Ethiopia. *Irrigation and Drainage Systems, 22*, 145-158.

Tidiane Sall, Amadou, Chiari, Tiberio, Legesse, Wasihun, Seid-Ahmed, Kemal, Ortiz, Rodomiro, Van Ginkel, Maarten, & Bassi, Filippo Maria. (2019). Durum wheat (Triticum durum Desf.): Origin, cultivation and potential expansion in Sub-Saharan Africa. *Agronomy, 9*(5), 263.

Tilahun, Webanchi Melkie, Bekeko, Zelalem, & Silesh, Fitsum. (2022). *IMPORTANCE OF WHEAT STEM RUST DISEASE (Puccinia graminis f. sp. tritici) IN NORTH SHEWA, ETHIOPIA, AND RESISTANCE OF DURUM WHEAT LANDRACE ACCESSIONS [Triticum turgidum subsp. durum (Desf.) Husn.] TO THE PREVAILING RACES OF THE PATHOGEN.* Haramaya University.

Tinker, NA, & Yan, W. (2006). Information systems for crop performance data. *Canadian journal of plant science, 86*(3), 647-662.

Tulu, Leta, & Wondimu, Addishiwot. (2019). Adaptability and yield stability of bread wheat (Triticum aestivum) varieties studied using GGE-biplot analysis in the highland environments of South-western Ethiopia. *African Journal of Plant Science, 13*(6), 153-162.

Vaezi, Behrouz, Pour-Aboughadareh, Alireza, Mohammadi, Rahmatolah, Mehraban, Asghar, Hossein-Pour, Tahmasb, Koohkan, Ehsan, . . . Siddique, Kadambot HM. (2019). Integrating different stability models to investigate genotype× environment interactions and identify stable and high-yielding barley genotypes. *Euphytica, 215*, 1-18.

Vavilov, Nikolaĭ Ivanovich. (1951). *The origin, variation, immunity and breeding of cultivated plants* (Vol. 72): LWW.

Wagaw, Kidanemaryam, Seyoum, Amare, Tadesse, Taye, Nega, Amare, Gebreyohannes, Adane, Bejiga, Tamirat, . . . Bogale, Meron. (2021). Distinguishing of stable genotypes and mega environment for grain yield performance of sorghum [Sorghum bicolor (L.) moench] genotypes using spatial analysis. *American Journal of Plant Sciences, 12*(3), 417-431.

Wardofa, Gadisa A, Asnake, Dawit, & Mohammed, Hussein. (2019). Gge biplot analysis of genotype by environment interaction and grain yield stability of bread wheat genotypes in central ethiopia. *Journal of plant breeding and genetics, 7*(2), 75-85.

Wodebo, Kibreab Yosefe, Tolemariam, Taye, Demeke, Solomon, Garedew, Weyessa, Tesfaye, Tessema, Zeleke, Muluken, . . . Sharma, Mamta. (2023). AMMI and GGE biplot analyses for mega-environment identification and selection of some high-yielding oat (Avena sativa L.) genotypes for multiple environments. *Plants, 12*(17), 3064.

Yan, Weikai, Kang, Manjit S, Ma, Baoluo, Woods, Sheila, & Cornelius, Paul L. (2007). GGE biplot vs. AMMI analysis of genotype‐by‐environment data. *Crop science, 47*(2), 643-653.

Yan, Weikai, & Tinker, Nicholas A. (2006). Biplot analysis of multi-environment trial data: Principles and applications. *Canadian journal of plant science, 86*(3), 623-645.

Yan, Weikai, Tinker, Nicholas A, Bekele, Wubishet A, Mitchell-Fetch, Jennifer, & Fregeau-Reid, Judith. (2019). Theoretical unification and practical integration of conventional methods and genomic selection in plant breeding. *Crop Breeding, Genetics and Genomics, 1*(2).

Yang, Fan, Zhang, Jingjuan, Liu, Qier, Liu, Hang, Zhou, Yonghong, Yang, Wuyun, & Ma, Wujun. (2022). Improvement and re-evolution of tetraploid wheat for global environmental challenge and diversity consumption demand. *International journal of molecular sciences, 23*(4), 2206.

Yismaw, Degenet. (2019). *GENOTYPE BY ENVIRONMENT INTERACTION DURUM WHEAT (Triticum turgidum GROWING AREAS OF GONDAR AND NORTH SHOA GRADUATE PROGRAM AND STABILITY ANALYSIS ssp. durum) GENOTYPES IN ZONES OF AMHARA REGION, ETHIOPIA M. Sc. Thesis By Yismaw Degenet Mogess November Bahir Dar, Ethiopia OF WHEAT 2019.*

Zenebe, Mulugeta. (2022). *Assessment and Management of Wheat Stem Rust (Puccinia graminis tiritici) Using Fungicides and Wheat Varietiesin Meket District, North Wollo, Ethiopia.*
