# Supplementary material for: Genotype by environment interaction and yield stability analysis of durum wheat (Triticum turgidum var. durum) varieties using AMMI and GGE biplot analysis in Amahara Region, Northwest Ethiopia
Source: PLoS One. 2026 Mar 13;21(3):e0342401. doi: 10.1371/journal.pone.0342401 (PMC12987450; doi:10.1371/journal.pone.0342401)
Supplement: Attachment — Submitted filename: Review report for the durum wheat manuscript PONE.docx [file pone.0342401.s005.docx]

**Review report for the durum wheat manuscript PONE-D-25-30070**

I have gone through the manuscript and found it relevant. The authors have a very good data, and tried to report their findings fairly. However, it cannot be published without undergoing major revision since it has lots of errors, missing information and technical mistakes.

**Title**: needs minor modification and I suggest it to be;

“Genotype by Environment Interaction and Yield Stability Analysis of **Durum Wheat** (Triticum turgidum var. durum) Varieties Using AMMI and GGE biplot Analysis **in Amahara state or Region**, Northwest Ethiopia”.

**Abstract**: this section needs major modification;

Abstract poorly composed though the study has good results.

- It should be written again because some data presented there has mistakes
- Some important result information not presented
- There are lots of grammatical & typographical mistakes
- I tried to correct some of them using the MSWord track review method in the attached file
- The AMMI analysis anova output was reported as the result of combined anova result

**Introduction**

- It has good information but presented poorly, it is highly disorganized, paragraphs should be reshuffled.
- In text citations didn’t follow the journal’s guideline
- Lots of sentences not clearly written. Several of them need to be rewritten
- Some topics like AMMI & GGE described repeatedly about their use and assumptions in detail, but no previous literatures on durum wheat presented that show their practical applications
- There is no statement or justifications why the authors selected the AMMI & GGE biplot models
- Problem statement or the need to do this study not described sufficiently
- Objectives of the research not stated clearly

**Materials & methods**

- The sub-title 2.1 should be modified; here you are giving the detail account of the study (experimental) area
- Table 1.Geographical description of the study area; It would be more informative if you can incorporate air temperature, and rainfall data of each year for the specific locations for the specific years. Specific geographic coordinates (including altitude) of the trial sites & if possible soil physic-chemical properties.
- 2.2. Experimental material and design; first 4-5 line needs to be rewritten
- Table 3; Can you include their pedigree for each variety? You better include their maturity period, yielding ability, areas of adaptation (recommendation), level of disease resistance, etc.
- 2.3. Data Collected; you have not described how you collected the data. At least you can insert citations of a published material that explains how you collected the data. So that readers get knowledge of how to do similar experiments using same or different materials.
- GGE biplot analysis- Which method or formula did you use? You need to show the formula you have used to compute the analysis

**Results and discussions**

- Same problem was observed in the in text citation as above
- 3.2. Performance of genotypes - What about the performance of these durum wheat genotypes in previous studies by other researchers? Please cite previous works that either agree or contradict with your findings to make your discussion complete
- What is this MSE? Is it the std of the mean values?
- 3.3. Genotype performance at each tested location- this sub-topic also lacks supporting literatures for the findings presented similar to sub-topic 3.2
- 3.4. Additive Main Effects and Multiplicative Interaction (AMMI) Analysis- One table is missing from AMMI analysis; the significant IPCAs (usually 1&2) give vector values for each genotype that indicates how much each genotype contributes to the GEI. Absolute value of the IPCA score determines its stability. Low value close to zero indicates the genotype being stable. You didn’t indicate which genotypes are stable using the AMMI analysis either using the table or the AMMI biplot 1 graph.
- Please refer the following publication for better understanding of the results and interpretation of AMMI analysis; Kassie MM, Abebe TD, Desta EA, Tadesse W. Multi-Environment Evaluation of Bread Wheat Genotypes for Yield Stability in Ethiopia Using AMMI and GGE-Biplot Analyses. Crop Breed Genet Genom. 2025;7(2):e250004.
- It appears that there is error in the table numbering, check it, see the comment in the manuscript file
- On page 17, the values you presented from table 7 about the proportions of the sum of squares of the AMMI anova seems to be incorrect. In addition, another table that lists IPCA scores for each genotype & even environments is missing. The IPCA scores help in identifying the relative contribution of genotypes for the GEI determines which genotype is stable/unstable. Please read the paper Kassie, et al (2025) and some recently published papers in durum wheat by Liyew et al. (2025) too.
- Figure 3, the mega environment grouping not clearly displayed
- Figure 4, concentric circles not visible
- Page 26, there is unfinished sentence; either correct it or delete it.
- 3.7 stability parameter; here, you have produced a beautiful table (**the last table**) for the stability analysis, but it was not exploited fully. You explained only one of the stability models that favours high grain yield. Please use the most recent stability models too like ASU, YSI
- 5. References; Please use the journal’s guideline for in text citations & style of referencing
