# Supplementary material for: Genotype by environment interaction and yield stability analysis of durum wheat (Triticum turgidum var. durum) varieties using AMMI and GGE biplot analysis in Amahara Region, Northwest Ethiopia
Source: PLoS One. 2026 Mar 13;21(3):e0342401. doi: 10.1371/journal.pone.0342401 (PMC12987450; doi:10.1371/journal.pone.0342401)
Supplement: Attachment — Submitted filename: Review report for the durum wheat manuscript PONE send.docx [file pone.0342401.s006.docx]

**Manuscript. Number: PONE-D-27-30070**

**Journal:** Plos one

**Title:** Genotype by Environment Interaction and Yield Stability Analysis of Durum Wheat (*Triticum turgidum* var. *durum*) Varieties Using AMMI and GGE biplot Analysis in Amahara Region, Northwest Ethiopia

**Dear Editor and Reviewers**

We would like to thank both the editor and the reviewers for your contribution to this manuscript by filtering out the technical and editorial errors in the scientific research writing that could increase the reliability and scientific exchange of the research output. We also admire your effort to see the details of our manuscript taking your golden time and we value all the comments. Further, we would be very glad to publish our manuscript in the Plos one journal if accepted. We believe that we have addressed all comments as suggested in the text and point-by-point response. Hence, kindly consider our revisions accordingly.

**Response to the comments**

**Title section reviewer comment**.

**Title**: needs minor modification and I suggest it to be;

“Genotype by Environment Interaction and Yield Stability Analysis of **Durum Wheat** (Triticum turgidum var. durum) Varieties Using AMMI and GGE biplot Analysis **in Amahara state or Region**, Northwest Ethiopia”.

**Response**_ Thank you for your valuable comments. We have modified our manuscript title based on the reviewer’s feedback and suggestions.

**Genotype by Environment Interaction and Yield Stability Analysis of Durum Wheat** (***Triticum turgidum* var. *durum***) **Varieties Using AMMI and GGE biplot Analysis in Amahara Region, Northwest Ethiopia**

**Abstract section reviewer comment**.

**Abstract**: this section needs major modification;

1. Abstract poorly composed though the study has good results.

**Response**_ We have updated the section by incorporating the main results into the abstract.

2. It should be written again because some data presented there has mistakes

**Response:** We have rewritten and updated the entire abstract based on the study results.

3. Some important result information not presented

**Response**_ We have updated it

4. There are lots of grammatical & typographical mistakes

**Response**_ All grammatical and typographical errors have been corrected as per the reviewer’s comment.

5. I tried to correct some of them using the MSWord track review method in the attached file

**Response**_Thank you for your valuable comments. We have reviewed them and made the necessary corrections.

6. The AMMI analysis Anova output was reported as the result of combined anova result

**Response**_ We have corrected the AMMI analysis ANOVA output to accurately present it separately from the combined ANOVA results.

**Introduction section reviewer comment**.

1. It has good information but presented poorly, it is highly disorganized, paragraphs should be reshuffled.

**Response**_ the necessary corrections have been made, and the paragraphs have been reorganized to improve clarity and structure.

2. In text citations didn’t follow the journal’s guideline

**Response**_ In-text citations have been revised in accordance with the journal’s guidelines.

3. Lots of sentences not clearly written. Several of them need to be rewritten

**Response**_ We have rewritten unclear sentences to improve clarity and readability

4. Some topics like AMMI & GGE described repeatedly about their use and assumptions in detail, but no previous literatures on durum wheat presented that show their practical applications

**Response**_ We have revised the sections on AMMI and GGE to reduce repetition and included relevant previous literature on their applications in related crops.

5. There is no statement or justifications why the authors selected the AMMI & GGE biplot models

**Response**_ We have added a clear justification for selecting the AMMI and GGE biplot models, highlighting their suitability for analyzing genotype by environment interactions in our study

6. Problem statement or the need to do this study not described sufficiently

**Response**_ The problem statement has been strengthened to provide a more comprehensive justification for conducting this study.

7. Objectives of the research not stated clearly

**Response** _We have revised the objectives section to clearly and explicitly state the research goals

**Materials & methods section reviewer comment**.

1. the sub-title 2.1 should be modified; here you are giving the detail account of the study (experimental) area

**Response** _ we update it

2. Table 1.Geographical description of the study area; It would be more informative if you can incorporate air temperature, and rainfall data of each year for the specific locations for the specific years. Specific geographic coordinates (including altitude) of the trial sites & if possible soil physic-chemical properties.

**Response** _ We have included air temperature and rainfall data for each year and the specific geographic coordinates, including altitude, of the trial sites. However, soil physicochemical properties were not available and thus could not be included

3. 2.2. Experimental material and design; first 4-5 line needs to be rewritten

**Response-**We have rewritten the first 4–5 lines of section 2.2 to improve clarity and coherence

4. Table 3; Can you include their pedigree for each variety? You better include their maturity period, yielding ability, areas of adaptation (recommendation), level of disease resistance, etc.

**Response-** We have included the pedigree, maturity period, yielding ability, recommended areas of adaptation, and disease resistance levels for each variety in Table 3 as suggested.

5. 2.3. Data Collected; you have not described how you collected the data. At least you can insert citations of a published material that explains how you collected the data. So that readers get knowledge of how to do similar experiments using same or different materials.

**Response-** We have included a detailed description of the data collection methods along with relevant citations to guide readers on how to conduct similar experiments.

6. GGE biplot analysis- Which method or formula did you use? You need to show the formula you have used to compute the analysis

**Response-** The manuscript now includes a detailed description of the GGE biplot analysis method along with the corresponding formula, as requested

**Results and discussions section reviewer comment**

- Same problem was observed in the in text citation as above

1. 3.2. Performance of genotypes - What about the performance of these durum wheat genotypes in previous studies by other researchers? Please cite previous works that either agree or contradict with your findings to make your discussion complete

**Response-** Relevant literature discussing the performance of durum wheat genotypes in previous studies has been cited to contextualize and strengthen the discussion of our results.

2. What is this MSE? Is it the std of the mean values?

**Response-**Its MSE (Mean Square Error) is not the standard deviation.

3. 3.3. Genotype performance at each tested location- this sub-topic also lacks supporting literatures for the findings presented similar to sub-topic 3.2

**Response-** We have added relevant supporting literature to the sub-topic on genotype performance at each tested location.

4. 3.4. Additive Main Effects and Multiplicative Interaction (AMMI) Analysis- One table is missing from AMMI analysis; the significant IPCAs (usually 1&2) give vector values for each genotype that indicates how much each genotype contributes to the GEI. Absolute value of the IPCA score determines its stability. Low value close to zero indicates the genotype being stable. You didn’t indicate which genotypes are stable using the AMMI analysis either using the table or the AMMI biplot 1 graph.

**Response -** We have included the AMMI analysis results by adding Table 7, which presents YSI, ASV, IPCA1, and IPCA2 values. Based on these, we have identified and indicated the stable genotypes as per the reviewer’s suggestion.

5. Please refer the following publication for better understanding of the results and interpretation of AMMI analysis; Kassie MM, Abebe TD, Desta EA, Tadesse W. Multi-Environment Evaluation of Bread Wheat Genotypes for Yield Stability in Ethiopia Using AMMI and GGE-Biplot Analyses. Crop Breed Genet Genom. 2025;7(2):e250004.

**Response -** Thank you for the helpful reference. We have reviewed the suggested publication by Kassie et al. (2025) and incorporated relevant insights to enhance the interpretation and discussion of the AMMI analysis results in our manuscript.

6. It appears that there is error in the table numbering, check it, see the comment in the manuscript file

**Response -** We have carefully reviewed the manuscript and corrected the table numbering errors as pointed out.

7. On page 17, the values you presented from table 7 about the proportions of the sum of squares of the AMMI anova seems to be incorrect. In addition, another table that lists IPCA scores for each genotype & even environments is missing. The IPCA scores help in identifying the relative contribution of genotypes for the GEI determines which genotype is stable/unstable. Please read the paper Kassie, et al (2025) and some recently published papers in durum wheat by Liyew et al. (2025) too.

**Response -**We have corrected the values from Table 7 regarding the sum of squares proportions in the AMMI ANOVA and added the IPCA scores for each genotype and environment as subcomponents within Table 7. This addition facilitates identification of genotype stability and contribution to GEI. We also reviewed and incorporated insights from Kassie et al. (2025) and Liyew et al. (2025) to strengthen our analysis.

8. Figure 3, the mega environment grouping not clearly displayed

**Response –** We have updated Figure 3 to clearly display the mega-environment groupings, making them easier to identify

9. Figure 4, concentric circles not visible

**Response –** We have enhanced Figure 4 to improve the visibility of the concentric circles as per your comment

10. Page 26, there is unfinished sentence; either correct it or delete it.

**Response –** he unfinished sentence on page 26 has been corrected to ensure clarity and completeness

11. 3.7 stability parameter; here, you have produced a beautiful table (**the last table**) for the stability analysis, but it was not exploited fully. You explained only one of the stability models that favors high grain yield. Please use the most recent stability models too like ASV, YSI

**Response –**Thank you for the valuable suggestion. We have expanded the stability analysis section to include and discuss additional recent stability models such as ASV and YSI, providing a more comprehensive interpretation of the results

12. References; Please use the journal’s guideline for in text citations & style of referencing

**Response –** We have carefully revised the in-text citations and reference list to fully comply with the journal’s guidelines
