# Supplementary material for: Genotype by environment interaction and yield stability analysis of durum wheat (Triticum turgidum var. durum) varieties using AMMI and GGE biplot analysis in Amahara Region, Northwest Ethiopia
Source: PLoS One. 2026 Mar 13;21(3):e0342401. doi: 10.1371/journal.pone.0342401 (PMC12987450; doi:10.1371/journal.pone.0342401)
Supplement: Attachment — Submitted filename: PONE Response R2.docx [file pone.0342401.s008.docx]

**Manuscript. Number: PONE-D-27-30070	R2**

**Journal:** **PLOS ONE**

**Title: Genotype by Environment Interaction and Yield Stability Analysis of Durum Wheat** (***Triticum turgidum* var. *durum***) **Varieties Using AMMI and GGE biplot Analysis in Amahara Region, Northwest Ethiopia**

**Dear Editor and Reviewers**

We would like to thank both the editor and the reviewers for your contribution to this manuscript by filtering out the technical and editorial errors in the scientific research writing that could increase the reliability and scientific exchange of the research output. We also admire your effort to see the details of our manuscript taking your golden time and we value all the comments. Further, we would be very glad to publish our manuscript in the Plos one journal if accepted. We believe that we have addressed all comments as suggested in the text and point-by-point response. Hence, kindly consider our revisions accordingly.

**Response to the General comment**

1. The manuscript requires thorough English language editing to improve grammar, clarity, and readability.

**Response-** We have carefully edited and reviewed the entire document to improve its English language quality and grammar throughout.

2. We have carefully addressed each reviewer’s comment point by point throughout the main document. All corrections and revisions have been made according to the reviewers’ suggestions and recommendations, and the changes are highlighted in color for easy reference.
3. Please review your reference list to ensure that it is complete and correct. If you have cited papers that have been retracted, please include the rationale for doing so in the manuscript text, or remove these references and replace them with relevant current references. Any changes to the reference list should be mentioned in the rebuttal letter that accompanies your revised manuscript. If you need to cite a retracted article, indicate the article’s retracted status in the References list and also include a citation and full reference for the retraction notice.

**Response -** Thank you for this valuable comment. We have carefully reviewed all references cited in the manuscript. Retracted or outdate articles have been removed and replaced with recent and relevant publications. Additionally, all remaining references have been verified for accuracy, completeness, and citation consistency. The updated reference list now includes only valid and current sources that directly support the findings of our study.

Based on the reviewer’s comment, here are the specific actions you must take:

1. Review all cited references — check that each one is relevant to your study and supports the statements in your manuscript.
2. Check for retracted papers — if any of your cited articles have been retracted, either:
   - Remove them and replace them with current, valid references, or
   - If you must cite them (rarely), clearly state that they are retracted and explain why they are still relevant.
3. Verify accuracy — ensure all reference details (authors, year, title, journal, volume, pages, DOI) are correct and properly formatted.
4. Add missing references — include any relevant studies suggested by reviewers if they strengthen your work.
5. Mention these updates in your rebuttal letter — briefly note that the reference list has been reviewed, corrected, and updated as per the reviewer’s suggestion.

**Response-** Thank you for the valuable feedback. We have carefully reviewed and updated the reference list as per the reviewer’s suggestions. All cited works have been checked for relevance, accuracy, and retraction status.
